# Activated protein C promotes hematopoietic stem and progenitor cell quiescence and engraftment

**DOI:** 10.1038/s44321-026-00529-y

**Published:** 2026-10-09

**Authors:** Simon J Pöllmann, Marcel G E Rommel, Leal Oburoglu, Tanja Hirch, Fabiola Valdivia-Francia, Kah Mun Siow, Timon Menzi, Julia Ackva, Franziska Schenk, Matthias J Neeracher, Ataman Sendoel, Janine Reichenbach, E Camilla Forsberg, Ute Modlich

**Affiliations:** 1 Division of Gene and Cell Therapy, Institute for Regenerative Medicine (IREM), University of Zurich, 8952 Schlieren, Switzerland; 2 Institute for the Biology of Stem Cells, University of California, Santa Cruz, CA 95064 USA; 3 Biomolecular Engineering, University of California, Santa Cruz, CA 95064 USA; 4 Translational Stem Cell Biology, Institute for Regenerative Medicine (IREM), University of Zurich, 8952 Schlieren, Switzerland; 5 Department of Somatic Gene Therapy, University Children’s Hospital Zurich, 8008 Zurich, Switzerland; 6 Center for Applied Biotechnology and Molecular Medicine (CABMM), University of Zurich, 8057 Zurich, Switzerland

**Keywords:** Haematology, Stem Cells & Regenerative Medicine

## Abstract

Hematopoietic stem cells (HSCs) are the definitive source of all blood cells and replenish them for life. The endothelial protein C receptor (EPCR) which signals via its coreceptor protease-activated receptor 1 (PAR1) is expressed on a subpopulation of highly quiescent HSCs with high engraftment potential after transplantation. We demonstrate that EPCR/PAR1 on human CD34^+^ hematopoietic stem and progenitor cells (HSPCs) can be activated by both thrombin (THR) and activated protein C (aPC), inducing distinct signaling. PAR1 activation by THR in HSPCs led to Ca^2+^ flux, phosphorylation of CREB and ERK1/2 and increased glycolysis. In contrast, aPC activated CREB and AKT, but not ERK1/2, Ca^2+^ flux or metabolic changes. Functionally, stimulation of human CD34^+^ cells with aPC decreased differentiation and enhanced clonogenic potential. aPC counteracted the enforced proliferation of HSPCs by interleukin 1α in vitro. Short stimulation of CD34^+^ cells with aPC enhanced their short-term serial engraftment in immunodeficient mice. Our findings identify aPC/PAR1 signaling as a key target supporting CD34^+^ HSPC maintenance, stress resistance and regeneration, while THR/PAR1 signaling has opposing effects.

## Synopsis

The G protein-coupled receptor PAR1 is highly expressed on human CD34^+^ HSPCs and is a co-receptor of the HSC marker EPCR. PAR1 can be activated by aPC or THR, resulting in biased signaling with opposing effects on HSPC fate.

- THR/PAR1 signaling induces Ca^2+^ mobilization and CREB and ERK1/2 activation and promotes metabolic activation and differentiation of HSPCs in vitro.
- aPC/PAR1 signaling activates CREB and AKT and maintains HSPC quiescence in vitro.
- Pharmacological modulation of THR/PAR1 signaling with Parmodulin 2 rescues the THR-induced phenotype of HSPCs.
- aPC improves human hematopoietic engraftment in NBSGW mice, enabling serial short-term multilineage reconstitution, whereas THR impairs engraftment.

## Abstract (web summary)

The G-protein-coupled receptor PAR1 is highly expressed on human CD34^+^ HSPCs and is a co-receptor of the HSC marker EPCR. PAR1 can be activated by aPC or THR, resulting in biased signaling with opposing effects on HSPC fate.

## Graphical abstract

**Figure:**
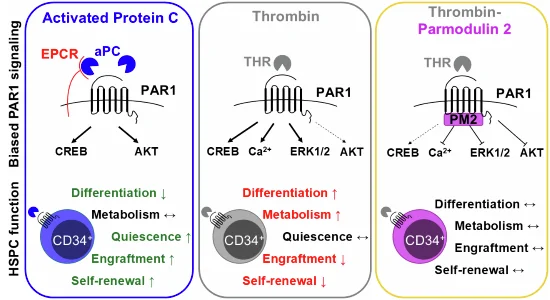

The paper explained **Problem**

Hematopoietic stem cells (HSCs) are at the top of the hematopoietic hierarchy. They produce all mature blood cells throughout life. Clinically, HSC transplantations can be used as a curative approach for many blood cell disorders. In some cases, such as in gene therapy, HSCs are cultivated ex vivo before transplantation. However, low engraftment of HSCs after transplantation endangers therapy success. New strategies are needed to preserve HSC quiescence and engraftment potential to overcome low engraftment of HSCs. The endothelial protein C receptor (EPCR) is expressed on a subset of human HSCs, which are highly quiescent and have high engraftment potential. EPCR signals via its coreceptor protease-activated receptor 1 (PAR1), however this signaling in human HSCs remained underexplored.

**Results**

We report that PAR1 is expressed on most human HSCs from mobilized peripheral blood and bone marrow, whereas EPCR expression on uncultivated cells is very low. The PAR1 agonists activated protein C (aPC) and thrombin (THR) can both activate PAR1 on human CD34^+^ cells in vitro. aPC induced CREB and AKT activation and maintained HSPC quiescence as evidenced by reduced cell divisions in vitro and transcriptional downregulation of DNA replication genes. aPC stimulation of CD34^+^ cells led to enhanced multilineage humanization and short-term engraftment of immature CD34^+^CD38^-^ cells in primary and secondary transplanted NBSGW mice compared to controls. THR, on the other hand, caused phosphorylation of ERK1/2 and CREB, as well as mobilization of Ca^2+^ in the cytosol. THR caused aberrant metabolic activation, driving glycolysis in CD34^+^ cells and did not alter cell cycling. After 1 h stimulation with THR, the differentiation of CD34^+^ cells in vitro was increased, and secondary short-term engraftment in transplanted NBSGW mice failed.

**Impact**

This work highlights the underappreciated role of aPC/PAR1 signaling in human CD34^+^ HSPC, which can be used to maintain HSPC function in vitro and to improve the engraftment potential of human HSPCs in the NBSGW mouse model. Our findings could be used in the future to improve the efficacy of HSC transplantations, especially if it involves ex vivo culture, benefitting patients.

## Introduction

Hematopoietic stem cells (HSCs) are multipotent and capable of replenishing all blood cells. They are therefore crucial to maintain a functional blood and immune system. HSCs possess extensive self-renewal capacity and reside mostly in a quiescent state. Their stem cell potential is maintained by cellular and cytokine support, as well as low oxygen levels in the bone marrow (BM) niche (Ugarte and Forsberg, 2013; Spencer et al, 2014). Clinically, HSCs are used to treat many blood disorders, either in HSC transplantations or in HSC-directed gene therapies. Although often life-saving, these therapies can be challenging since ex vivo gene therapies require cultivation and gene modification procedures, potentially reducing HSC function and compromising therapy success (Williams et al, 2025).

The endothelial protein C receptor (EPCR) is expressed on a subpopulation of adult and fetal, murine and human HSCs (Balazs et al, 2006; Kent et al, 2009; Iwasaki et al, 2010; Subramaniam et al, 2019; Lin and Trumpp, 2023) with high quiescence, even during inflammatory conditions (Rabe et al, 2020; Rommel et al, 2022; Munz et al, 2023; Bisht et al, 2026). Lentiviral re-expression of EPCR in MPL-deficient murine HSCs reestablished quiescence and cured aplastic anemia (Kohlscheen et al, 2019). In contrast to murine Lin^-^Sca-1^+^c-kit^+^CD34^-^CD48^-^CD150^+^ HSC from the mouse BM with up to 90% EPCR expression, the levels in human BM CD34^+^CD38^-^CD90^+^ HSC are reported to be below 1% (Subramaniam et al, 2019; Anjos-Afonso et al, 2022) or in CD34^+^CD38^-/low^CD45RA^-^ below 0.03% (Anjos-Afonso et al, 2022). However, also in human hematopoiesis, EPCR expression marks HSCs with high long-term multilineage repopulation potential: EPCR^+^CD34^+^CD38^-^CD45RA^-^ HSCs have an exceptionally high repopulating stem cell frequency of 1/3 cells in xenograft mice (Anjos-Afonso et al, 2022). Importantly, EPCR expression on human CD34^+^ cells increases with cultivation using UM171 and also marks in vitro expanded HSC with long-term repopulation potential (Becker et al, 2023; Fares et al, 2017; Che et al, 2022).

Yet, the mechanism of EPCR signaling in maintaining HSC quiescence remains underexplored. EPCR can bind activated protein C (aPC) (Fukudome & Esmon, 1994), which activates the EPCR-coreceptor protease-activated receptor 1 (PAR1) by cleavage at Arg46 (Riewald et al, 2002). In endothelial cells, this G protein-coupled receptor then conveys cytoprotective and anti-inflammatory cues (Molinar-Inglis et al, 2021). In contrast, thrombin (THR) activates PAR1 via cleavage at Arg41, leading to opposing and pro-inflammatory signals (Molino et al, 1997). THR/PAR1 signaling can be modulated pharmacologically by parmodulins, which bind to the intracellular face of PAR1 and potentially bias THR/PAR1 signaling towards cytoprotective signaling (de Ceunynck et al, 2018; Künze and Isermann, 2023). PAR1 expression in HSCs has been reported (Gur-Cohen et al, 2015; Steidl et al, 2002).

Gur-Cohen et al investigated PAR1 signaling in murine HSCs, showing that both aPC and THR can act on murine HSCs: aPC limits NO production, lowers CDC42 activation, increases affinity towards the HSC homing factor CXCL-12 and overall promotes BM retention of HSCs (Gur-Cohen et al, 2015). However, multiple aspects of PAR1 activation, like Ca^2+^ signaling (Brass et al, 1994), metabolic activation (Aibibula et al, 2018) and changes in gene expression (Mac et al, 2025) described in other cell types remain unexplored in HSCs and progenitor cells, especially human CD34^+^ hematopoietic stem and progenitor cells (HSPCs). Given our knowledge of aPC and THR signaling in endothelial cells, platelets and murine HSCs, we hypothesized that PAR1 activation in human CD34^+^ HSPCs could be supportive or detrimental to HSPC function, depending on the activator. In our study, we systematically investigated aPC- and THR-induced PAR1 signaling in human CD34^+^ HSPCs and whether PAR1 activation supports human hematopoietic engraftment, which may be exploited clinically.

## Results

### Differential EPCR and PAR1 expression on human CD34^+^ subpopulations

**Figure 1:**
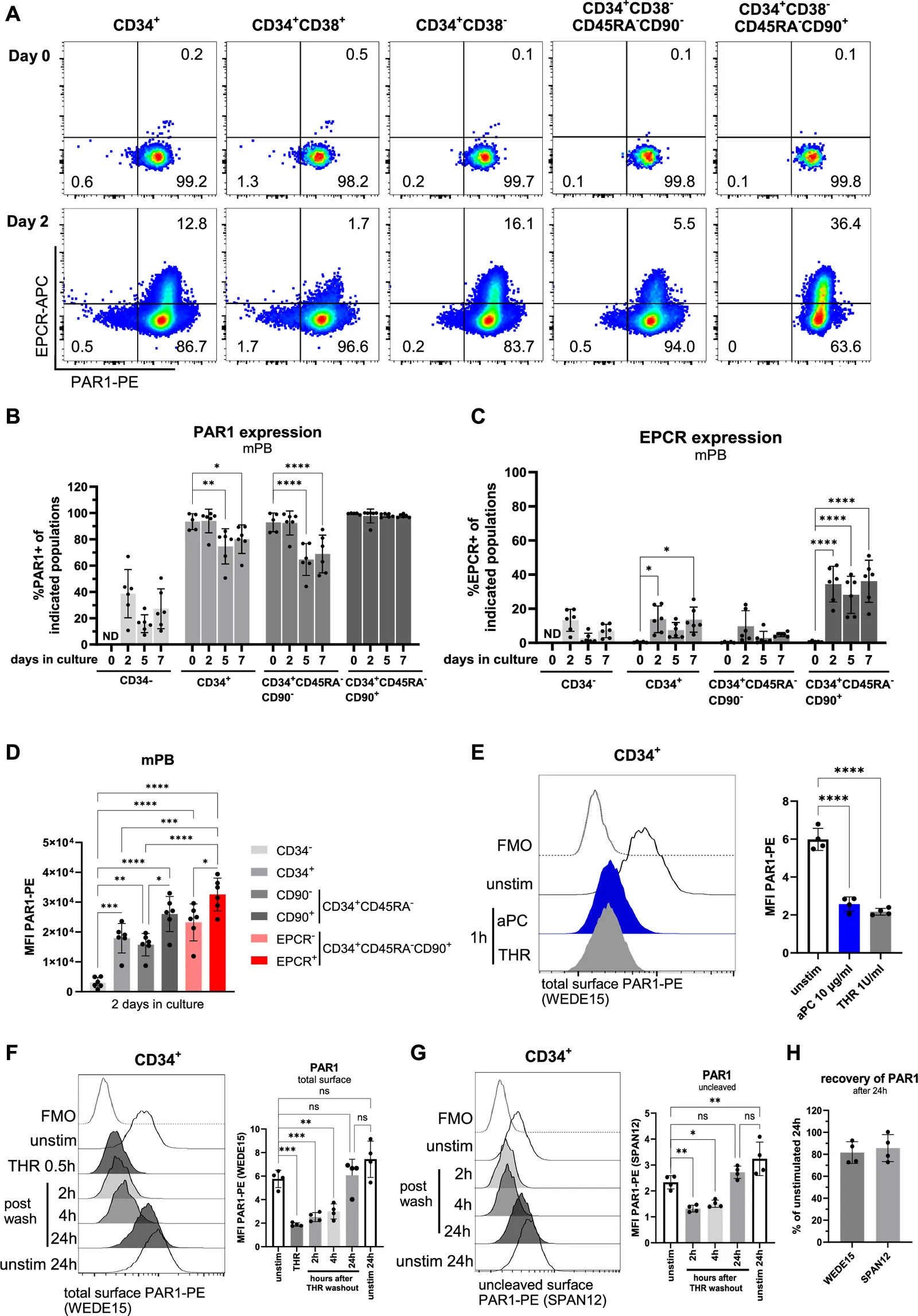
PAR1 and EPCR Expression on human mobilized CD34^+^ subpopulations. (**A**) Representative flow cytometry (FC) plots of PAR1 and EPCR expression on subsets of CD34^+^ cells from human mobilized peripheral blood (mPB), freshly thawed and 2 days in culture. (**B**) PAR1 expression during cultivation of mPB samples on indicated populations, day 0 *n* = 5 mPB donors, day 2, 5, and 7 *n* = 6 donors; ND: no CD34-negative cells detected. (**C**) EPCR expression during cultivation in mPB samples on indicated populations, day 0 *n* = 5 donors, day 2, 5, and 7 *n* = 6 donors. (**D**) Median fluorescence intensity (MFI) of PAR1-PE (clone WEDE15) on indicated populations of mPB samples two days in culture, *n* = 6 donors. (**E**) CD34^+^ cells were cultured for 2 days and subsequently stimulated with aPC (10 µg/ml) or THR (1 U/ml) for 1 h. PAR1 cell surface expression was assessed by FC, *n* = 4 individual mPB donors. (**F**) Two days cultured CD34^+^ cells were stimulated with THR for 0.5 h, washed and recovery of PAR1 cell surface expression was assessed as indicated with anti-PAR1-PE antibody clones WEDE15 (cleaved and not cleaved PAR1) and (**G**) SPAN12 (only not cleaved PAR1), *n* = 4 individual mPB donors. (**H**) Recovery of PAR1 MFI was calculated %recovery = (MFI_24h after THR washout_/MFI_unstimulated after 24h_) × 100% for both PAR1 antibody clones respectively, *n* = 4 individual mPB donors. Statistical analysis: all data were presented as mean ± SD. (**B**,** C**) two-way ANOVA with Holm–Šídák’s multiple comparison test. (**C**) See Appendix Table S1 for further statistics comparing EPCR expression between CD90^+^ and CD90^-^ populations. (**D**,** E**) One-way ANOVA with Tukey’s multiple comparison test. (**F**,** G**) one-way ANOVA with Holm-Šídák’s multiple comparison, all tested comparisons shown. (**H**) Unpaired two-tailed *t*-test; ns not significant, *P ≤ 0.05, **P ≤ 0.01, ***P ≤ 0.001, ****P ≤ 0.0001, exact *P* values can be found in Appendix Table S3. Source data are available online for this figure.

**Figure EV1:**
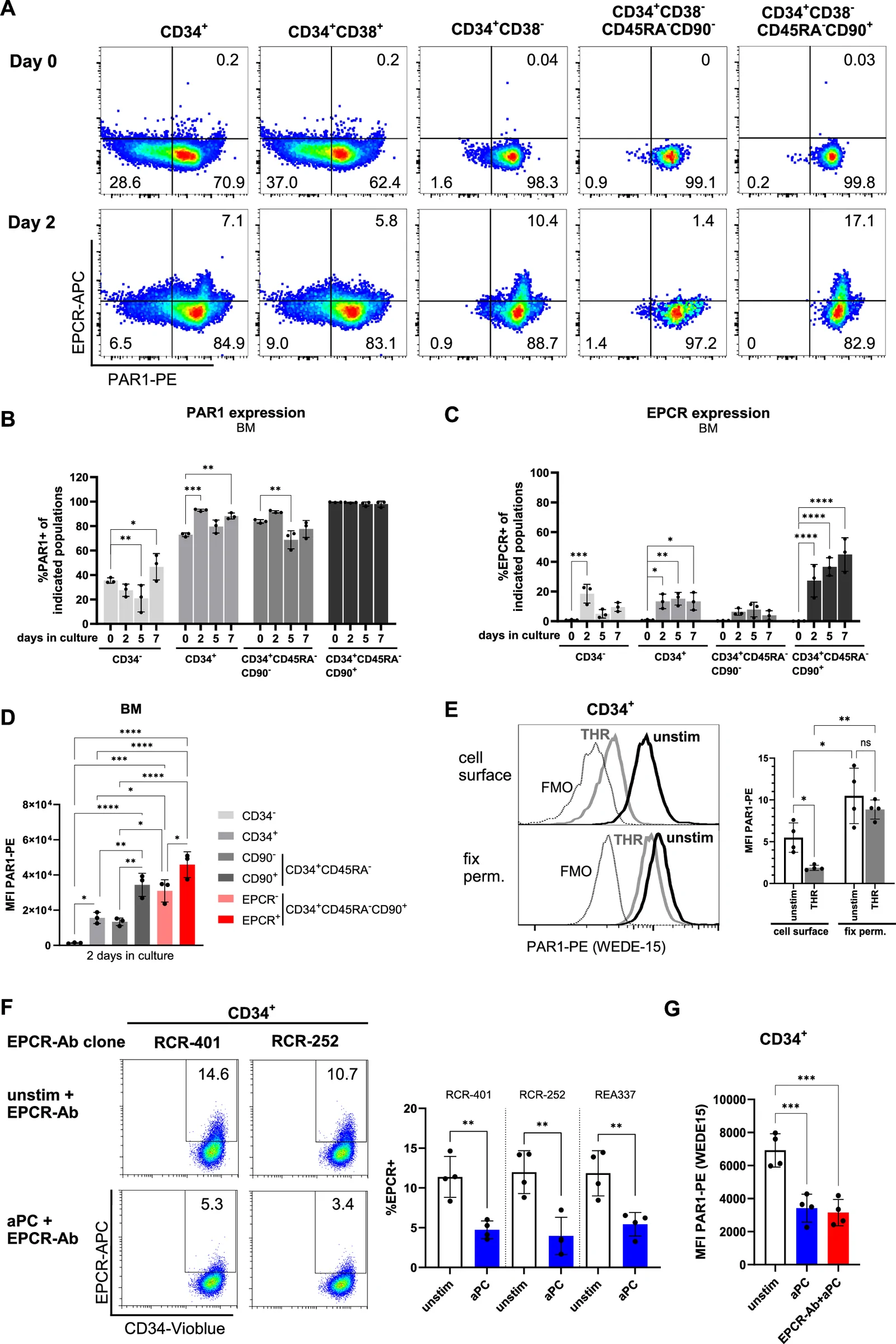
Expression of PAR1 and EPCR of human bone marrow CD34^+^ subpopulations. (**A**) Representative FC plots showing EPCR and PAR1 expression on indicated populations of BM CD34^+^ samples at day 0 and 2 days in culture. (**B**,** C**) PAR1 and EPCR expression during in vitro cultivation are shown in BM samples on the indicated populations, *n* = 3 BM donors. (**D**) MFI of PAR1-PE on indicated populations of BM samples 2 days in culture, *n* = 3 BM donors. (**E**) Cell surface and intracellular (fix perm.) PAR1 expression quantified in 2-day cultivated CD34^+^ cells with and without THR stimulation for 1 h, PAR1 FMOs are depicted as the dotted thin-lined histograms, *n* = 4 mPB donors. (**F**) Staining of EPCR on CD34^+^ cells after 2 days in culture, cells were either incubated for 1 h with aPC (10 µg/ml) at 37 °C or left unstimulated in normal medium before cell surface marker staining was conducted, three different commercially available monoclonal anti-EPCR antibodies were used (clones RCR-401 and RCR-252, also known as REA337), *n* = 4 mPB donors. (**G**) Cell surface PAR1 staining of 2-day cultivated CD34^+^ cells with aPC alone (blue) or in the presence of EPCR-blocking antibody REA337 (red), *n* = 4 mPB donors. Statistical analysis: (**B**–**G**) all data were presented as mean ± SD. (**B**,** C**,** E**) Two-way ANOVA with Holm–Šídák’s multiple comparison test. (**C**) See Appendix Table S1 for two-way ANOVA comparing CD90^+^ with CD90^−^ cells for each day. (**D**,** G**) One-way ANOVA with Tukey’s multiple comparison test. (**F**) Unpaired two-tailed *t*-tests; ns not significant, *P ≤ 0.05, **P ≤ 0.01, ***P ≤ 0.001, ****P ≤ 0.0001, exact *P* values can be found in Appendix Table S9.

We first investigated PAR1 and EPCR expression on healthy adult human CD34^+^ cells collected from peripheral blood after mobilization with G-CSF (mPB) and from bone marrow aspirate (BM, Figs. 1A and EV1A). PAR1 was expressed on most mPB CD34^+^ cells (93.5 ± 6.1%, Fig. 1A,B), whereas CD34^+^ cells in BM samples were only 73.6 ± 0.3% PAR1 positive (Fig. EV1A,B), confirming previous observations of increased PAR1 expression on mobilized CD34^+^ cells (Steidl et al, 2002). Nearly all CD34^+^CD38^-^ cells, multipotent progenitors (MPP, CD34^+^CD38^-^CD45RA^-^CD90^-^) and HSCs (CD34^+^CD38^-^CD45RA^-^CD90^+^ as defined by Notta et al, 2011) were PAR1 positive, regardless of CD34^+^ source (Figs. 1A and EV1A; Appendix Fig. S1).

We next investigated the maintenance of PAR1 expression during cultivation. As CD38 expression has been shown to be altered during cultivation and in relation to the cell cycle state of human HSPCs (McKenzie et al, 2007; Dorrell et al, 2000), we did not use this marker for cultivated cells and defined CD34^+^CD45RA^-^CD90^+^ as a population of bona-fide HSC-enriched expanded cells. During cultivation in an HSC expansion medium (Fares et al, 2017), most CD34^+^ cells remained PAR1^+^ and expression only declined after 5 days to 74.6 ± 13.4% in the CD34^+^CD45RA^-^CD90^-^, but not the CD34^+^CD45RA^-^CD90^+^ population (Fig. 1B. Interestingly, there was an initial increase on PAR1 expression on BM CD34^+^ cells during cultivation to levels similar to mPB CD34^+^ cells (73.6 ± 0.3% at day 0 to 92.7 ± 1.4% at day 2, Fig. EV1B). Differentiated CD34^−^ cells from mPB or BM cells are mostly PAR1 negative (Figs. 1B,D and Fig. EV1B,D).

We confirmed prior observations that EPCR expression was very low on freshly thawed adult human HSCs from BM and mPB (<1%, Figs. 1A,C and EV1A,C) (Anjos-Afonso et al, 2022; Subramaniam et al, 2019). EPCR expression increased in culture if the cultivation medium contained LSD1 degraders, like UM171 (Fares et al, 2017; Subramaniam et al, 2020). In our case, EPCR was expressed on 34.4 ± 10.4% on mPB CD34^+^CD45RA^-^CD90^+^ cells and 27.3 ± 10.9% on BM CD34^+^CD45RA^-^CD90^+^ after 2 days with UM171 (Figs. 1C and EV1C). Consistently, EPCR was expressed more frequently in CD34^+^CD45RA^-^CD90^+^ compared with their CD90^-^ counterparts (Appendix Table S1).

After 2 days in culture, the highest median fluorescence intensity (MFI) of PAR1 was found on CD34^+^CD45RA^-^CD90^+^EPCR^+^ and strongly declined with differentiation in both BM and mPB samples (Figs. 1D and EV1D). In conclusion, although PAR1 expression was not exclusive to HSCs or EPCR-expressing cells, it was expressed at high levels on human adult HSCs, regardless of cell source. PAR1 expression remained stable on the HSC-enriched population under our tested culture conditions, only declining with ongoing differentiation.

### PAR1 activation by thrombin or aPC leads to internalization of the receptor

PAR1 activation by THR in other cell types leads to its cleavage and internalization (Molino et al, 1997). Consistent with this, a 1 h incubation with THR or aPC reduced cell surface PAR1 on CD34^+^ cells (Fig. 1E). Twenty-four hours after short-term THR stimulation and subsequent washout of THR, surface PAR1 expression returned to baseline (Fig. 1F). Unlike most G protein-coupled receptors, cleaved PAR1 is not recycled, but lysosomally degraded since its mode of activation is irreversible (Grimsey et al, 2016). By using antibody clones specifically distinguishing THR-cleaved and non-cleaved (intact) PAR1 protein, we confirmed the presence of uncleaved cell surface PAR1 after 24 h (Fig. 1G,H), indicating the recovered PAR1 to be a new protein. Although we detected a decline in cell surface PAR1 after THR stimulation, there were no differences in total PAR1 levels in response to THR activation after permeabilization of the cells (Fig. EV1E), indicating that PAR1 is restored from intracellular stores. A high concentration of aPC (10 µg/ml) decreased binding of anti-EPCR antibody clones RCR-401 and RCR-252 (also known as REA337, Fig. EV1F) to UM171-cultivated CD34^+^ cells, as these antibodies and aPC compete for binding to EPCR. However, preincubation with an EPCR-blocking antibody did not reduce PAR1 activation/internalization (Fig. EV1G), indicating that at high concentrations, aPC activated PAR1 independent of EPCR. In summary, both THR and aPC led to rapid clearance of PAR1 from the cell surface, which was reinstalled after 24 h.

### Thrombin induces Ca^2+^ flux in CD34^+^ CD45RA^−^CD90^+^ cells

**Figure 2:**
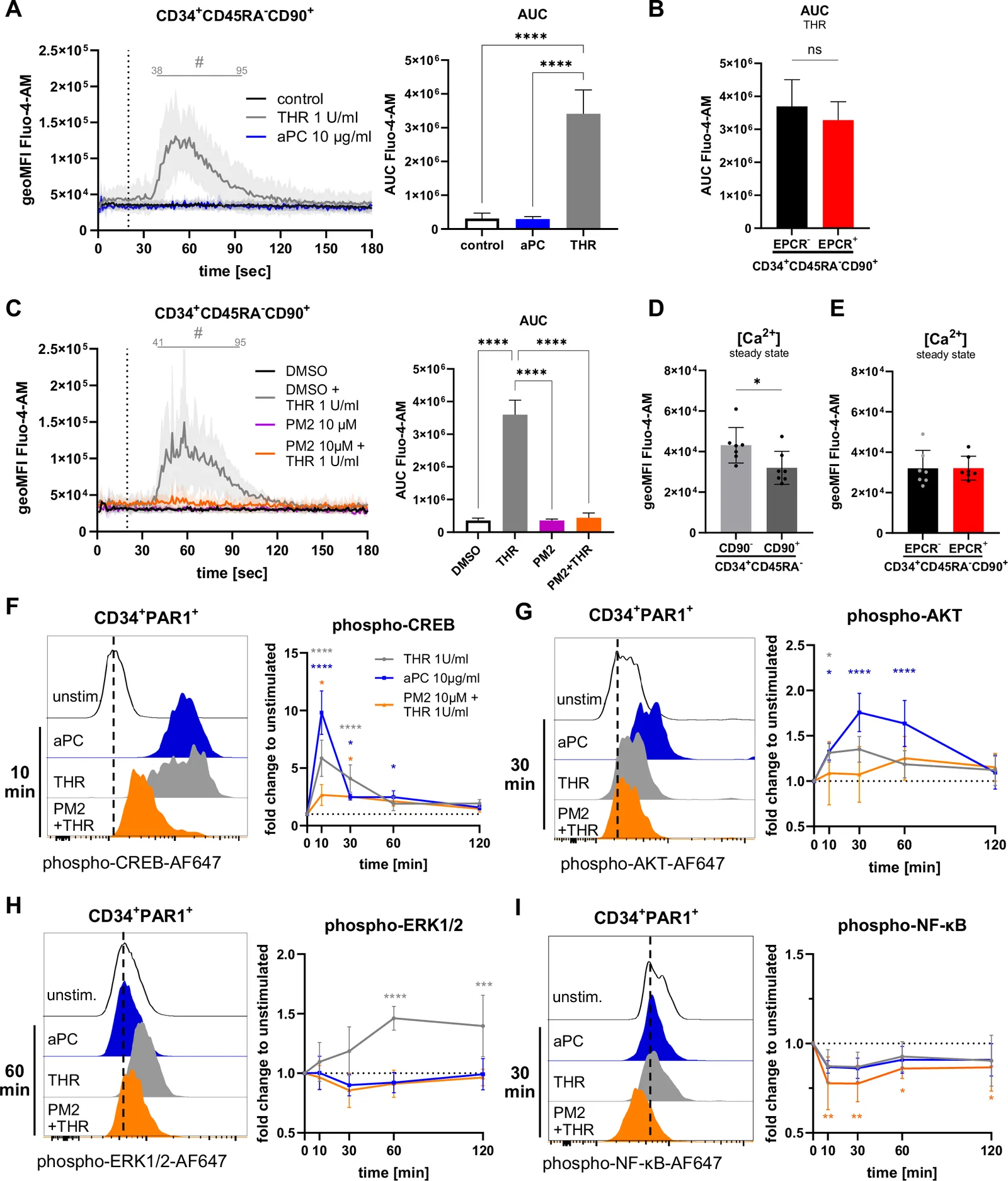
Differential signaling of PAR1 in CD34^+^ cells. (**A**) Changes in relative cytosolic Ca^2+^ concentration in CD34^+^CD45RA^−^CD90^+^ cells were measured fluorometrically with Fluo-4-AM by FC (geoMFI geometric mean fluorescent intensity), dotted line at 20 s indicates addition of indicated stimuli, bar chart depicts area under the curve (AUC) of Ca^2+^ flux curves, control (PBS) and THR *n* = 7 mPB donors, aPC *n* = 3 mPB donors. (**B**) AUC of Fluo-4-AM geoMFI is shown for CD34^+^CD45RA^−^CD90^+^EPCR^+/−^ cells after THR (1 U/mL) stimulation, *n* = 6 mPB donors. (**C**) Changes in relative cytosolic Ca^2+^ concentration in CD34^+^CD45RA^-^CD90^+^ cells. Cells were pretreated with Parmodulin 2 (PM2, 10 µM) or DMSO for 5 min prior to stimulation with THR, where indicated. The dotted line marks the timepoint of THR addition; AUC is shown in the bar chart, *n* = 3 mPB donors. (**D**) Relative cytosolic Ca^2+^ concentration in expanded CD34^+^CD45RA^−^CD90^+/−^cells, *n* = 7 mPB donors. (**E**) Relative cytosolic Ca^2+^ concentration in expanded CD34^+^CD45RA^−^CD90^+^EPCR^+/−^, *n* = 6 mPB donors. (**F**–**I**) Phosphorylation of downstream molecules CREB, AKT, ERK1/2, and NF-κB of PAR1 was analysed via Phosphoflow in 7 days expanded CD34^+^ cells. Cells were stimulated with THR, aPC (10 µg/mL) or PM2 + THR for 10, 30, 60, and 120 min. Graphs depict MFI fold change in CD34^+^PAR1^+^ cells, *n* = 4 mPB donors, mean of one to two technical replicates per donor/condition. Statistical analysis: All data were presented as mean ± SD. (**A**,** C**,** F**–**I**) Kinetic curves: two-way ANOVA with Holm–Šídák’s multiple comparison test. (**A**,** C**) #*P* ≤ 0.05, numbers indicate time interval (start to finish) in which significant findings were made. (**A**,** C**) Bar charts: One-way ANOVA with Tukey’s multiple comparison test. (**B**,** D**,** E**) Unpaired two-tailed *t*-test; (**F**–**I**) for additional statistical testing between stimuli, see Appendix Table S2; *P ≤ 0.05, **P ≤ 0.01, ***P ≤ 0.001, ****P ≤ 0.0001, exact *P* values can be found in Appendix Table S4. Source data are available online for this figure.

**Figure EV2:**
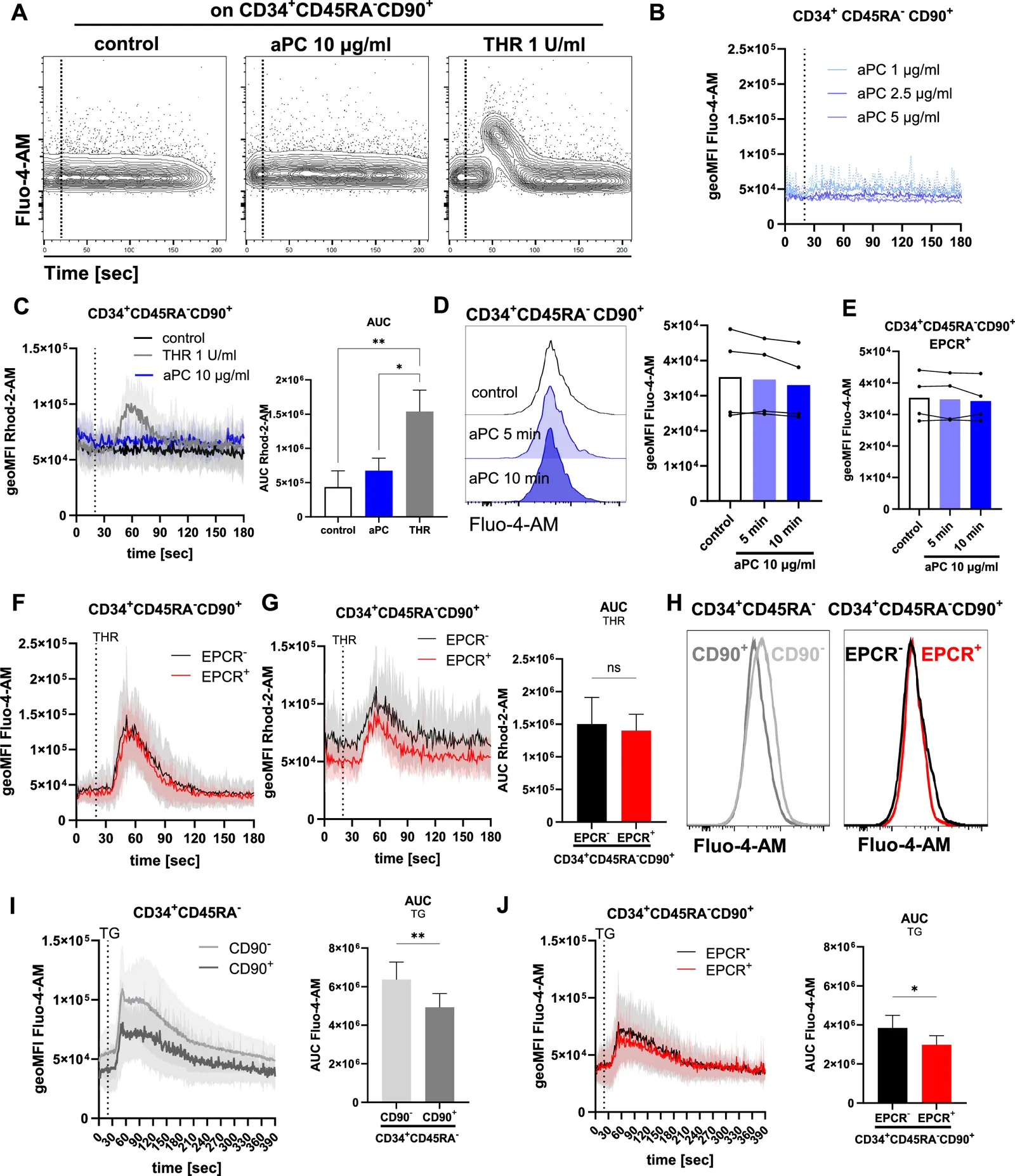
PAR1-Ca^2+^-signaling in human CD34^+^ cells. (**A**) Representative flow cytometry plots of the Ca^2+^ flux assay, shown is the fluorescent signal of Fluo-4-AM over time of the sample recording gated on CD34^+^CD45RA^−^CD90^+^ cells. The dotted line marks the 20 s, where indicated stimuli were added to the sample. (**B**) Fluo-4-AM kinetics of cells stimulated with various aPC doses (1, 2.5, 5 µg/ml) after 20 s, marked by the dotted line, *n* = 3 mPB donors. (**C**) Ca^2+^ kinetics in CD34^+^CD45RA^−^CD90^+^ cells in response to aPC, THR or control, measured with Rhod-2, *n* = 3 mPB donors. (**D**) aPC does not lead to changes in Ca^2+^ concentration after 5 and 10 min in CD34^+^CD45RA^−^CD90^+^ and (**E**) CD34^+^CD45RA^−^CD90^+^EPCR^+^ cells, *n* = 4 mPB donors. (**F**) Fluo-4-AM kinetics and (**G**) Rhod-2 kinetics and AUC of CD34^+^CD45RA^−^CD90^+^EPCR^+/−^ cells after THR stimulation (1 U/ml), (**F**) *n* = 6 mPB donors, (**G**) *n* = 3 mPB donors. (**H**) Representative plots of Fluo-4-AM signal at steady state in CD34^+^CD45RA^−^CD90^+/−^ and CD34^+^CD45RA^−^CD90^+^EPCR^+/−^. (**I**) Ca^2+^ flux caused by Thapsigargin (TG, 1 µM), shown is kinetic curves and areas under the curves (AUC) of CD34^+^CD45RA^−^CD90^+/−^, *n* = 6 mPB and (**J**) CD34^+^CD45RA^−^CD90^+^EPCR^+/−^, *n* = 5 mPB. Statistical analysis: (**B**,** C**,** F**–**J**) data presented as mean ± SD. (**D**,** E**) The bar represents the mean, and the dots connected by a line represent the data from each individual donor. (**C**–**E**) Bar chart: one-way ANOVA with Tukey’s multiple comparison test. (**G**,** I**,** J**) Bar chart: unpaired two-tailed *t*-test; ns not significant, *P ≤ 0.05, **P ≤ 0.01, ***P ≤ 0.001, ****P ≤ 0.0001, exact *P* values can be found in Appendix Table S10.

PAR1 activation by THR in platelets causes flux of Ca^2+^ ions from cellular storage compartments, like the endoplasmic reticulum, into the cytosol, where it acts as a second messenger. In expanded CD34^+^CD45RA^-^CD90^+^ cells, PAR1 activation by THR likewise caused a roughly 60 s long Ca^2+^ flux (Figs. 2A and EV2A,C), detected by increased fluorescence of the Ca^2+^ indicators Fluo-4 and Rhod-2, whereas aPC stimulation did not change free cytosolic Ca^2+^ levels in doses ranging from 1 to 10 µg/mL (Figs. 2A and EV2B) and incubation times up to 10 min (Fig. EV2D,E). Ca^2+^ flux did not differ between EPCR^+^ and EPCR^-^ CD34^+^CD45RA^-^CD90^+^ cells when stimulated with THR (Fig. 2B and EV2F,G). Preincubation of CD34^+^ cells with the allosteric PAR1 modulator Parmodulin 2 (PM2) (Gandhi et al, 2018) completely blocked THR-induced Ca^2+^ flux (Fig. 2C).

Steady state levels of free cytosolic Ca^2+^ were significantly lower in expanded CD34^+^CD45RA^−^CD90^+^ cells compared with CD34^+^CD45RA^−^CD90^−^ cells, independent of EPCR expression, measured by Fluo-4 (Figs. 2D,E and EV2H). Thapsigargin (TG), an endoplasmic reticulum Ca^2+^-ATPase (SERCA) inhibitor, causes a rise of cytosolic Ca^2+^ in most cell types (Thastrup et al, 1990). TG led to significantly higher Ca^2+^ flux in CD34^+^CD45RA^−^CD90^+^EPCR^-^ cells than in CD34^+^CD45RA^−^CD90^+^EPCR^+^ cells; similarly, CD34^+^CD45RA^−^CD90^−^ cells had a higher Ca^2+^ flux than CD34^+^CD45RA^−^CD90^+^ cells (Fig. EV2I,J). The steady state cytosolic Ca^2+^ levels were not altered with regard to EPCR expression. These findings indicate that total Ca^2+^ mobilization capacity in EPCR^+^CD34^+^CD45RA^−^CD90^+^ cells is lower. Low cytosolic Ca^2+^ levels and low Ca^2+^ flux has been shown to protect HSCs from metabolic activation and proliferation (Umemoto et al, 2018; Luchsinger et al, 2019), potentially contributing to higher quiescence of EPCR^+^ HSCs.

### EPCR/PAR1 signals via phosphorylation of ERK1/2, AKT, and CREB

**Figure EV3:**
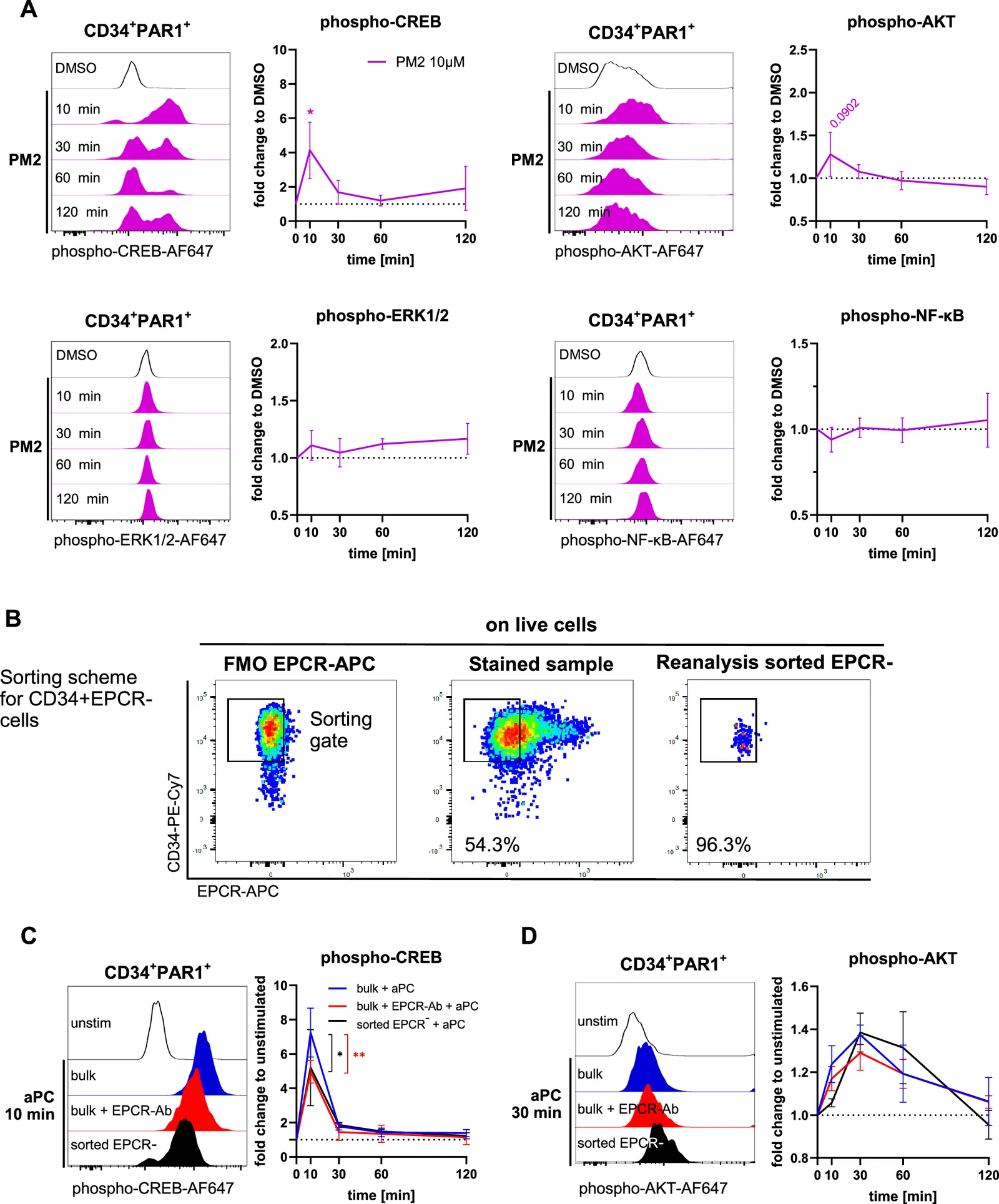
EPCR-independent PAR1 signaling in CD34^+^PAR1^+^ cells. (**A**) phosphoflow analysis of phosphorylated CREB, AKT, ERK1/2 and NfκB of 7-day expanded CD34^+^ cells, pregated on CD34^+^PAR1^+^ cells in response to PM2 (10 µM) for the indicated time period, depicted as fold change to the DMSO control, *n* = 3 mPB donors. (**B**) Representative FACS sorting scheme to sort CD34^+^EPCR^−^ cells at day 7 for phosphoflow analysis. (**C**) Phosphoflow analysis of CREB and (**D**) AKT in response to aPC, either on bulk expanded cells without (blue) or with EPCR-blocking antibody (clone REA337, red) or in sorted EPCR-negative CD34^+^ cells (back), *n* = 3 mPB donors. Statistical analysis: (**A**,** C**,** D**) Data presented as mean ± SD. (**A**) One-way ANOVA with Dunnett’s multiple comparison test compared to the DMSO control. (**C**,** D**) Two-way Anova with Holm–Šídák’s multiple comparison test; ns not significant, *P ≤ 0.05, **P ≤ 0.01, ***P ≤ 0.001, ****P ≤ 0.0001, exact *P* values can be found in Appendix Table S11.

To assess how PAR1 downstream targets were differentially activated by aPC and THR, we used phosphoflow analysis on 7 days expanded mPB CD34^+^ cells. Both aPC and THR stimulation induced phosphorylation of the transcription factor cAMP response element-binding protein (CREB, Fig. 2F) in CD34^+^PAR1^+^ cells. CREB activation was highest with 10 µg/mL aPC, inducing a tenfold increase in phospho-CREB MFI after 10 min, whereas THR induced a 5.8-fold increase at 10 min (Fig. 2F). aPC and THR both lead to phosphorylation of AKT, but aPC-mediated AKT activation was higher at 30 min and more prolonged (Fig. 2G). Strikingly, extracellular signal-regulated kinase 1/2 (ERK1/2) phosphorylation occurred only after THR stimulation, but not with aPC (Fig. 2H). Preincubation with PM2 inhibited THR-mediated AKT and ERK1/2 activation and reduced CREB activation considerably in human CD34^+^PAR1^+^ cells (Fig. 2G,H; Appendix Table S2). Although described in non-hematopoietic cells (Rahman et al, 2001), THR did not cause phosphorylation of NF-κB in CD34^+^PAR1^+^ cells, neither did aPC. PM2 + THR-stimulated cells showed a reduction of NF-κB below the baseline (Fig. 2I). Although PM2 has been shown to modulate THR/PAR1 signaling, there is evidence that PM2 alone can also induce signaling, specifically activating AKT (de Ceunynck et al, 2018). Addition of PM2 in the absence of THR caused a non-significant trend towards AKT activation after 10 min, significant CREB activation in a similar way as THR or aPC with a peak after 10 min in CD34^+^PAR1^+^ cells, but no ERK1/2 and NF-κB activation (Fig. EV3A). These results indicate that PM2 can act on PAR1 independent of THR.

To further address whether EPCR was essential for PAR1 activation by aPC, we FACS separated EPCR^-^ and EPCR^+^CD34^+^ cells and, because of the blocking function of the EPCR-specific antibody, compared the sorted populations with non-sorted CD34^+^ cells. Sorted EPCR^−^ (Fig. EV3B), and similarly EPCR-blocked cells, showed the same AKT activation with aPC but a significantly lower peak CREB activation, compared to the control non-sorted, non-EPCR-blocked cells (Fig. EV3C,D), indicating a largely EPCR-independent PAR1 activation and signaling with our high aPC concentration ex vivo. These data demonstrated ligand-biased PAR1 signaling in human HSPCs, with aPC preferentially activating CREB and AKT pathways, while THR predominantly triggered CREB and ERK1/2 activation without engaging NF-κB.

### CD34^+^ cells treated with aPC have a transcriptional profile of reduced cell cycle activity

**Figure 3:**
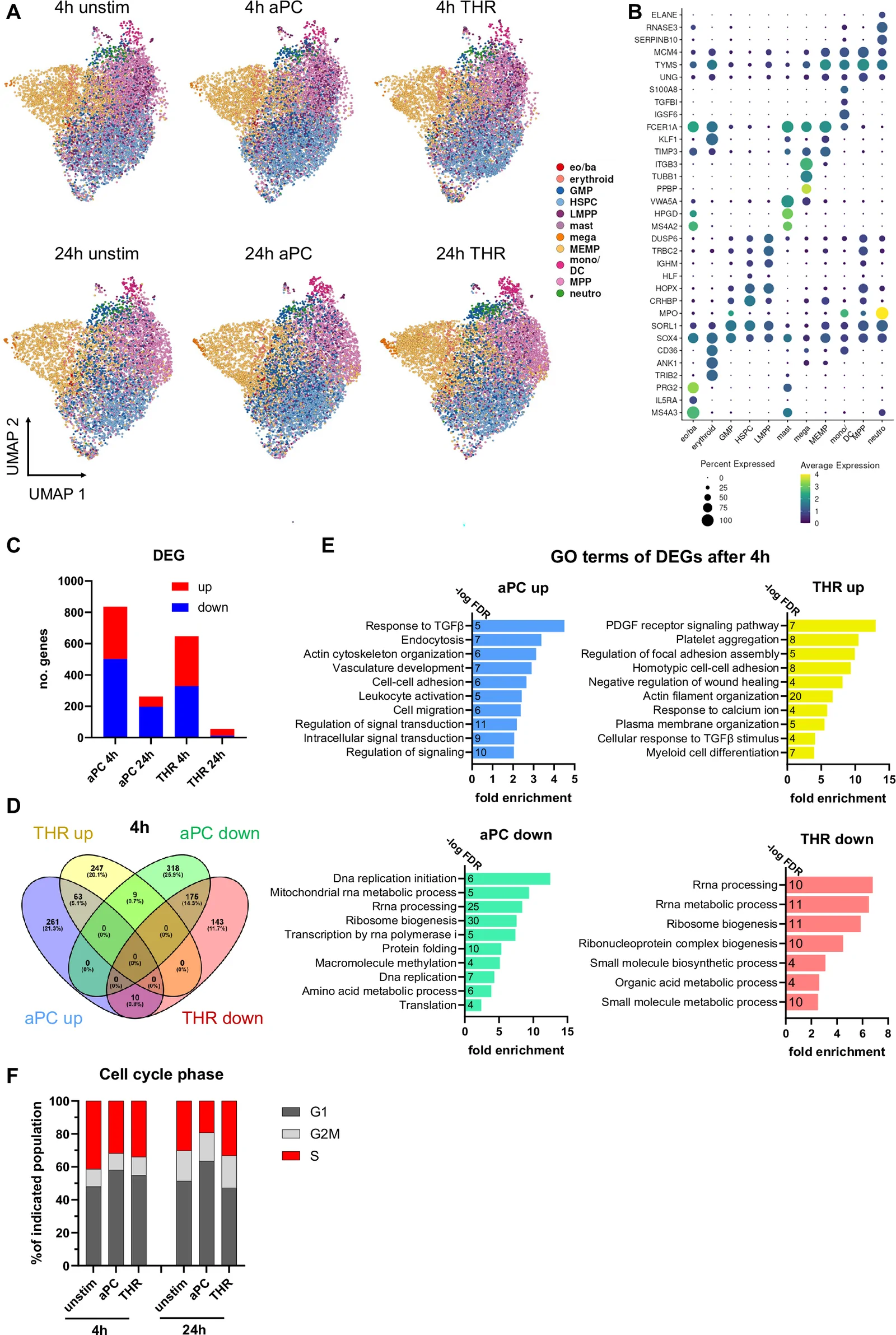
aPC and THR cause different changes in gene expression in human CD34^+^ cells. (**A**) UMAP clustering of CD34^+^ cells stimulated for 4 or 24 h with aPC (10 µg/ml) or THR (1 U/ml) or left unstimulated (unstim); cluster annotations are described in detail in the method section: lymphoid-primed multipotent progenitors (LMPPs), multipotent progenitors (MPPs), granulomonocytic progenitors (GMPs), megakaryocyte erythroid mast cell progenitors (MEMPs), megakaryocytes (mega), eosinophil/basophils (eo/ba), mast cells (mast), erythroid lineage cells (erythroid), neutrophils (neutro), monocytic/dendritic cells (mono/DCs), *n* = 1 mPB donor. (**B**) Dot plot depicting expression of marker genes for annotated clusters. (**C**) Differentially expressed genes (DEG) with a cutoff of log2 fold change of 0.25 and an adjusted *P* value ≤ 0.05 found in stimulated cells versus unstimulated control cells at 4 or 24 h timepoints. (**D**) Venn diagram depicting shared and unique upregulated and downregulated DEGs between aPC and THR at the 4 h timepoint. (**E**) Gene ontology (GO) biological processes enrichment of upregulated and downregulated DEGs after 4 h of stimulation, number in bar represents −log (FDR) rounded to nearest integer, all terms are below threshold of FDR ≤ 0.05. (**F**) Cell cycle phase distribution inferred from transcriptome signatures of unstimulated, aPC and THR-stimulated cells after 4 and 24 h.

**Figure EV4:**
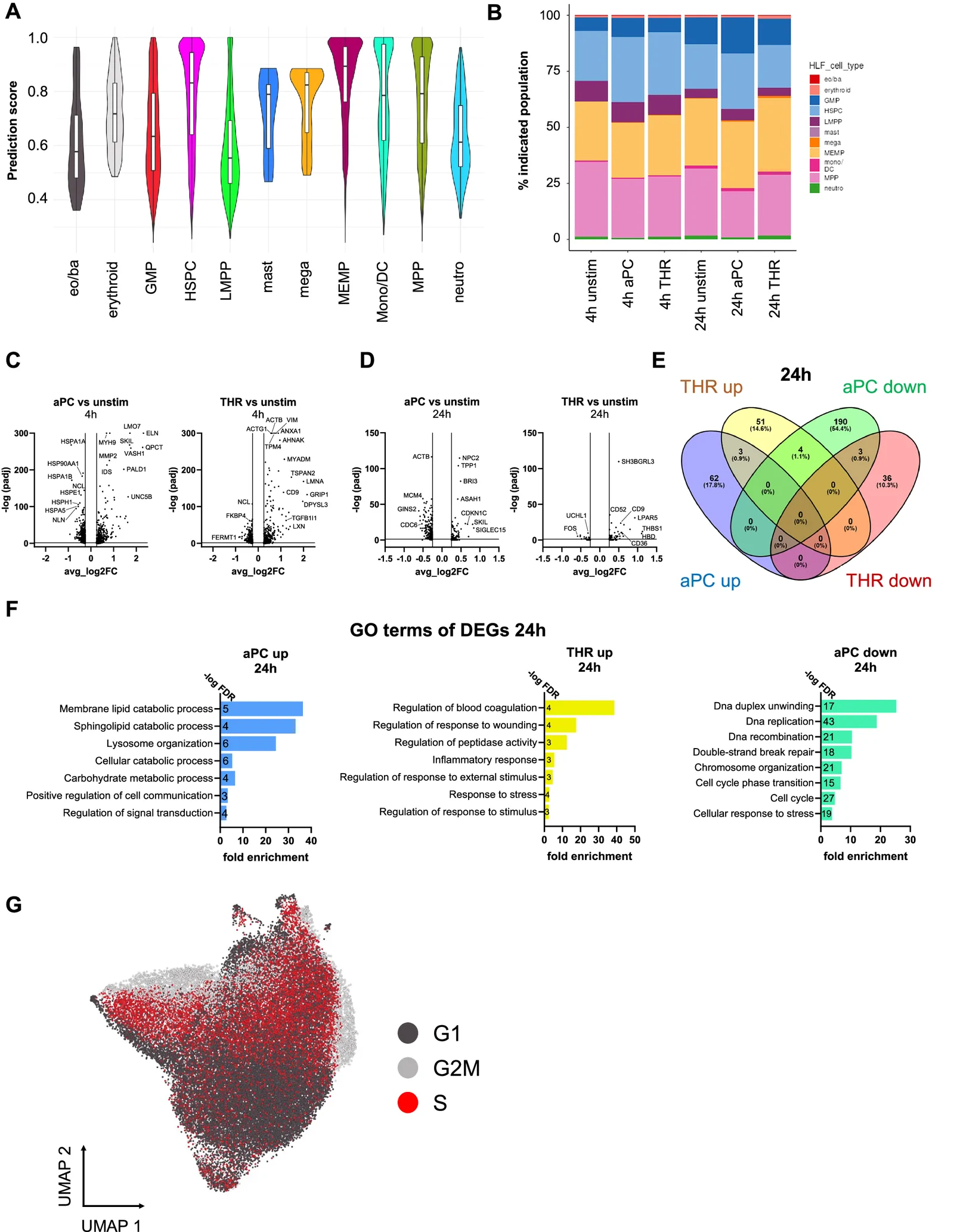
scRNAseq of stimulated CD34^+^ cells. (**A**) Prediction scores for each cluster are depicted *n* = 14,171 (MEMP), *n* = 13,713 (MPP), *n* = 11,989 (HSPC), *n *= 5018 (GMP), *n* = 3422 (LMPP), *n* = 578 (neutro), *n* = 499 (mono/DC), *n* = 490 (erythroid), *n* = 167 (mega), *n* = 107 (eo/ba), *n* = 14 (mast). (**B**) Proportions of clusters for each sample. (**C**) Volcano plot depicting DEG after 4 h and (**D**) 24 h with aPC or THR stimulation compared to unstimulated, *n* = 8602 cells (4 h unstim), *n* = 9024 cells (4 h aPC), *n* = 8170 cells (4 h THR), *n* = 6965 cells (24 h unstim), *n* = 8507 cells (24 h aPC), *n* = 8900 cells (24 h THR) from one donor in one experiment. (**E**) Venn diagram showing shared and unique DEG after 24 h. (**F**) GO biological processes terms enriched in up- and downregulated genes after 24 h with aPC and THR, of note: THR downregulated genes at 24 h were not enriched for any GO term, number in bar represents −log (FDR) rounded to nearest integer, all terms are below threshold of FDR <0.05. (**G**) UMAP depicting the cell cycle phase of CD34^+^ cells. Statistical analysis: (**A**) thick horizontal line indicates median, box indicates interquartile range and whiskers indicate range. (**C**,** D**) Wilcoxon rank-sum test with Bonferroni correction.

The presence of phosphorylated CREB, ERK, and AKT points to transcriptional activation in human CD34^+^ cells in response to PAR1 signaling. We therefore performed single-cell RNA sequencing of CD34^+^ cells 4 and 24 h after stimulation with aPC or THR. Cells were clustered using transcriptional and ABseq signatures and annotated using published data sets using the same cultivation medium (Lehnertz et al, 2021; Sauvageau, 2020a; Sauvageau, 2020b) (Fig. 3A,B), with good prediction scores for all clusters (Fig. EV4A). aPC or THR stimulation did not drastically change cluster sizes (Fig. EV4B). We detected 836 differentially expressed genes (DEGs) in CD34^+^ cells stimulated with aPC after 4 h and 647 DEGs after stimulation with THR, and lower numbers of DEGs after 24 h, especially after THR treatment (Figs. 3C and EV4C,D). The majority of DEGs were exclusive to aPC or THR treatments (Figs. 3D and EV4E). Upregulated genes after 4 h aPC or THR stimulation were involved in cell adhesion, actin cytoskeleton regulation and response to transforming growth factor beta (TGFβ, Fig. 3E), aPC additionally upregulated expression of genes important for cell migration. THR stimulation, but not aPC, induced genes involved in myeloid differentiation and Ca^2+^ signaling, confirming that only THR caused Ca^2+^ flux in these cells (Fig. 2A). THR downregulated organic acid metabolism (Fig. 3E), whereas aPC downregulated expression of genes involved in protein folding, translation and DNA replication, the latter especially at the 24 h timepoint (Fig. EV4F). Transcriptional cell cycle phase analysis indicated that more cells resided in G1 after aPC stimulation after 24 h (Figs. 3F and EV4G). Twenty-four hours after aPC stimulation, cells also upregulated genes in catabolic processes, indicating metabolic reprogramming of the cells, which was not the case after THR stimulation (Fig. EV4F).

### Thrombin rapidly induces metabolic activity in CD34^+^ cells

**Figure 4:**
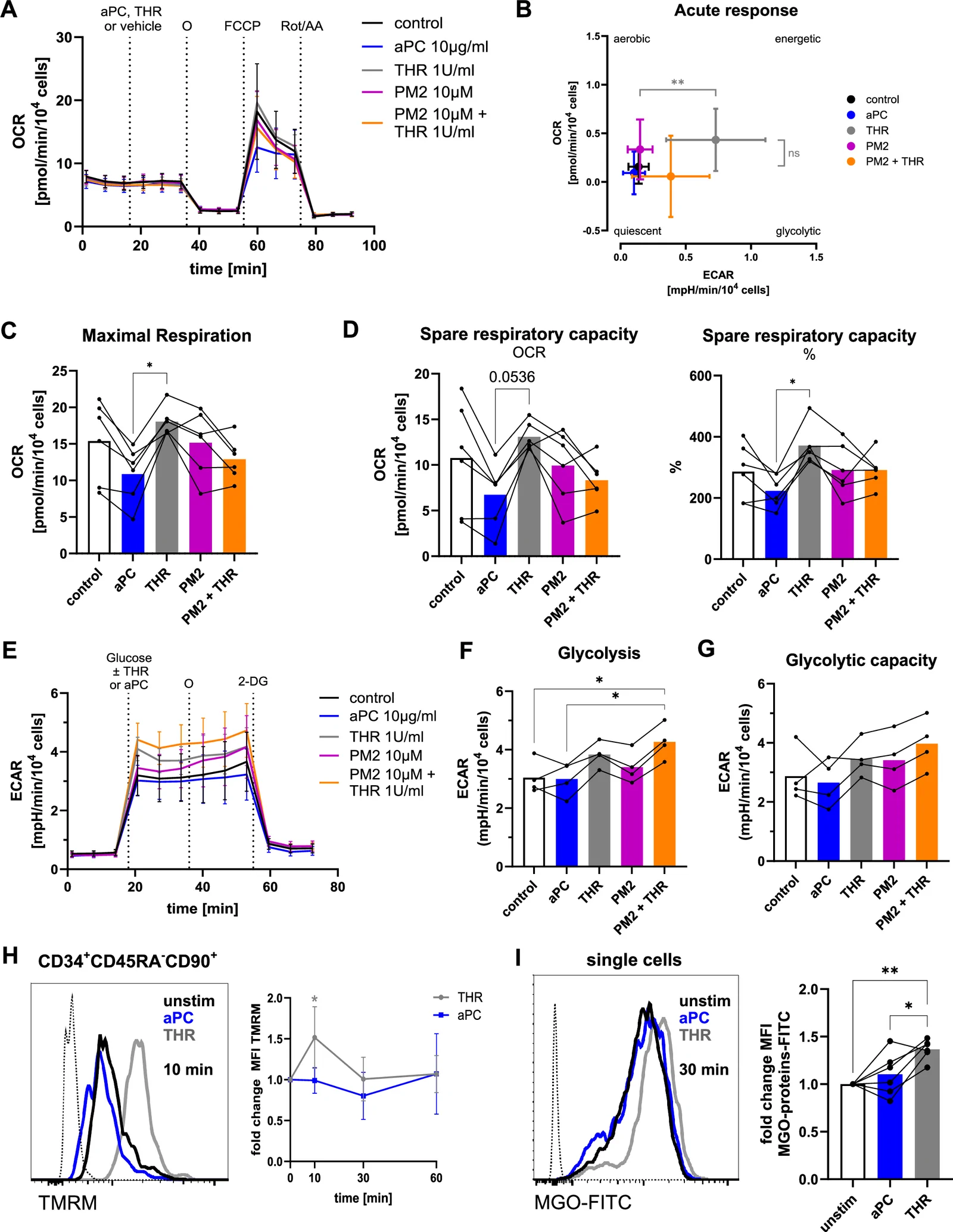
THR activates glycolysis in CD34^+^ cells. (**A**) Seahorse Mito Stress test performed with 2-day-cultivated CD34^+^ cells, oxygen consumption rate (OCR) is measured during stimulation with indicated stimuli, PM2 and DMSO (control) was added to the cells 1 h before start of the assay, aPC, THR or medium (vehicle) was injected after baseline recording, O Oligomycin, FCCP carbonyl cyanide-p-trifluoromethoxyphenylhydrazone, Rot/AA rotenone/antimycin A, *n* = 6 mPB donors. (**B**) Acute response of OCR and ECAR to indicated stimuli, *n* = 6 mPB donors. (**C**) Maximal respiration after FCCP injection and after addition of indicated stimuli, *n* = 6 mPB. (**D**) Spare respiratory capacity calculated as the difference between baseline and maximal respiration in OCR (left) and as a percentage (right), *n* = 6 mPB donors. (**E**) Seahorse glycolysis stress test performed on 2-day-cultivated CD34^+^ cells, ECAR of glucose-starved cells in response to co-injection of glucose±aPC or THR, CD34^+^ cells were pretreated for 1 h with either PM2 or DMSO (control), 2-DG 2-deoxy-D-glucose, *n* = 4 mPB donors. (**F**) Glycolysis rate and in response to indicated stimuli and glucose injection, measured as ECAR, *n* = 4 mPB donors. (**G**) Glycolytic capacity after oligomycin injection and after addition of indicated stimuli, *n* = 4 mPB donors. (**H**) MFI fold change relative to unstimulated control of the mitochondrial membrane potential dye TMRM (tetramethylrhodamine methyl ester) in response to THR (1 U/ml) or aPC (10 µg/ml) for 10, 30, 60 min in CD34^+^CD45RA^−^CD90^+^ cells. The dotted histogram represents cells stimulated with 100 µM FCCP for 30 min as a negative control, *n* = 4 mPB donors. (**I**) Flow cytometric quantification of methylglyoxal protein-adducts (MGO-protein-adducts) in CD34^+^ cells after incubation with PBS (control), aPC (10 µg/ml) or THR (1 U/ml) for 30 min, *n* = 6 mPB donors, dotted histogram shows unstained sample. Statistical analysis: (**A**,** C**–**G**) each dot represents the mean of technical triplicates to sextuplicates of one donor, connecting lines represent samples from one donor, bar height is the mean. (**B**,** H**) mean ± SD. (**B**–**D**,** F**,** G**,** I**) One-way ANOVA with Tukey’s multiple comparison test. (**H**) Two-way ANOVA with Holm–Šídák’s multiple comparison test; ns not significant, *P ≤ 0.05, **P ≤ 0.01, ***P ≤ 0.001, ****P ≤ 0.0001, exact *P* values can be found in Appendix Table S5. Source data are available online for this figure.

**Figure EV5:**
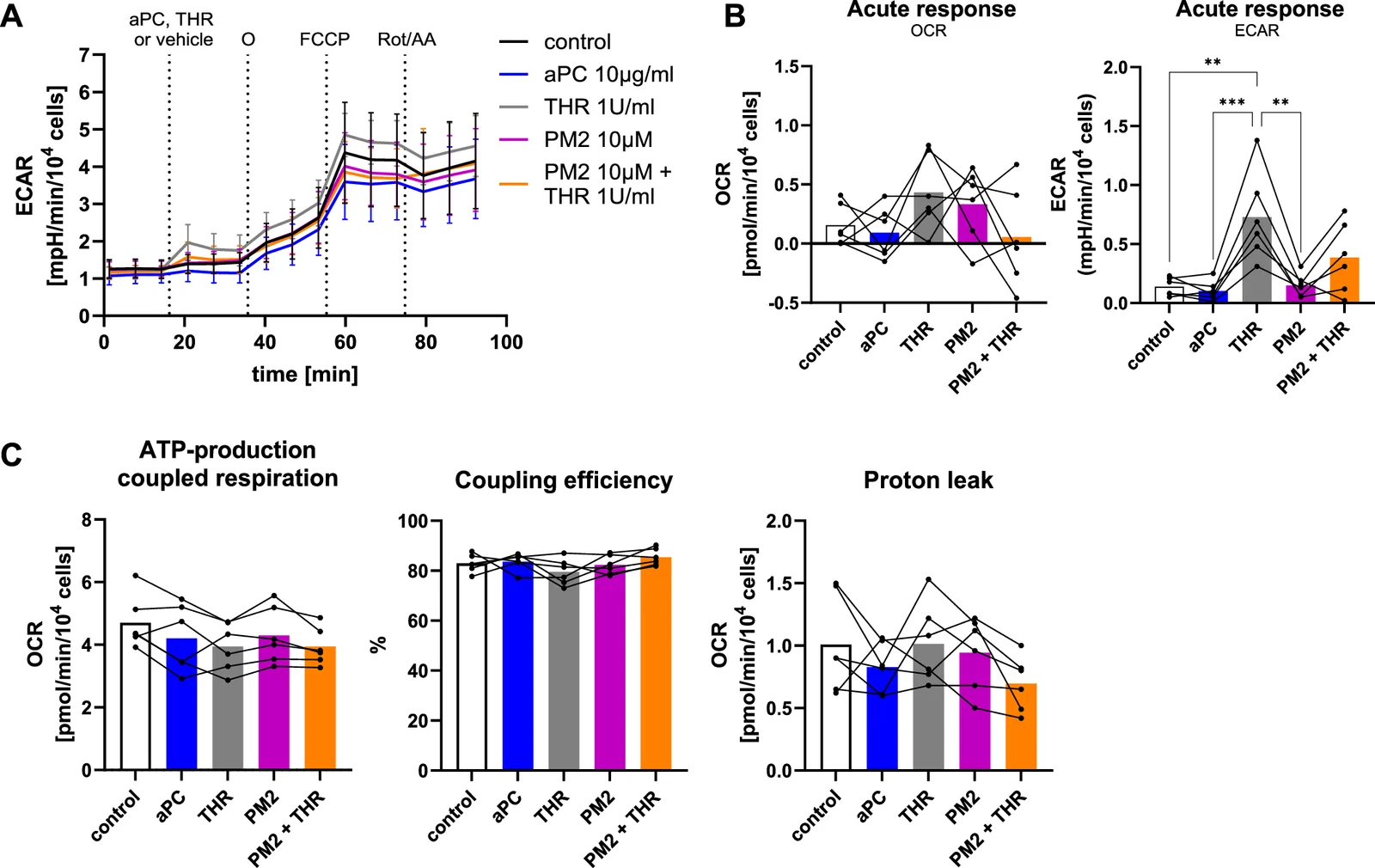
Metabolic parameters of CD34^+^ cells. (**A**) Extracellular acidification rate (ECAR) curves over the course of the Seahorse Mitostress test, dotted lines represent injection of indicated stimuli, *n* = 6 mPB donors. (**B**) Acute response of CD34^+^ cells to indicated stimuli, data shown here is also shown in Fig. 4B, *n* = 6 mPB donors. (**C**) Metabolic parameters derived from the Mitostress test of CD34^+^ cells, *n* = 6 mPB donors. Statistical analysis: (**A**) data were presented as mean ± SD. (**B**,** C**) Bar height represents the mean, lines connect samples from individual donors, one-way Anova with Tukey’s multiple comparison test; *P ≤ 0.05, **P ≤ 0.01, ***P ≤ 0.001, ****P ≤ 0.0001, exact *P* values can be found in Appendix Table S12.

Following our observation of changes in the transcription of metabolic genes, we profiled oxygen consumption rate (OCR), indicative of cellular respiration (Fig. 4A) and extracellular acidification rate (ECAR), indicative of glycolysis (Fig. EV5A), in 2-day cultivated human CD34^+^ HSPCs after THR or aPC stimulation using the Seahorse Analyzer. In other cell types, such as platelets, activation by THR rapidly increases glycolysis, but also oxidative phosphorylation (OXPHOS) to facilitate shape changes, degranulation and clotting (Aibibula et al, 2018).

THR significantly increased the ECAR after 20 min (acute response), while CD34^+^ cells stimulated with aPC, or pretreated with PM2 and stimulated with THR, did not change OCR and ECAR (Figs. 4B and EV5B). Also, maximal respiration and spare respiratory capacity were increased in THR-treated cells compared to aPC-treated CD34^+^ cells (Fig. 4C,D), but ATP-production, coupling efficiency or proton leak remained unchanged (Fig. EV5C). These results indicated that THR did not cause damage to mitochondrial function but rather induced short-term metabolic activation.

Co-injection of glucose ± THR or aPC to glucose-starved CD34^+^ cells (glycolysis stress test) showed a trend of increased glycolysis rate and a significant additive increase in cells that were pretreated with PM2 and stimulated with THR (Fig. 4E,F). Glycolytic capacity was also trending higher with THR and PM2 ± THR (Fig. 4G). In accordance with the higher metabolic activity of THR-stimulated cells, the mitochondrial membrane potential in CD34^+^CD45RA^−^CD90^+^ cells increased transiently after 10 min of THR stimulation (Fig. 4H).

Glycolysis can create reactive byproducts, such as methylglyoxal (MGO), which impairs cellular function due to the formation of protein adducts (advanced glycation end products, AGEs) (Allaman et al, 2015). Thirty minutes after THR stimulation of CD34^+^ cells, levels of MGO-protein adducts increased by 1.4-fold, whereas aPC did not alter MGO-protein levels (Fig. 4I).

Under physiological conditions, HSCs rely on low levels of glycolysis to fuel their energetic needs, protecting them from accumulation of reactive metabolic byproducts. Our experiments show that THR stimulation raises metabolic output, by increasing glycolysis, resulting in significant accumulation of AGEs, while aPC maintained a metabolically quiescent profile characteristic of functional HSCs, reflecting cytoprotective PAR1.

### aPC maintains CD34^+^ HSPC function in vitro

**Figure 5:**
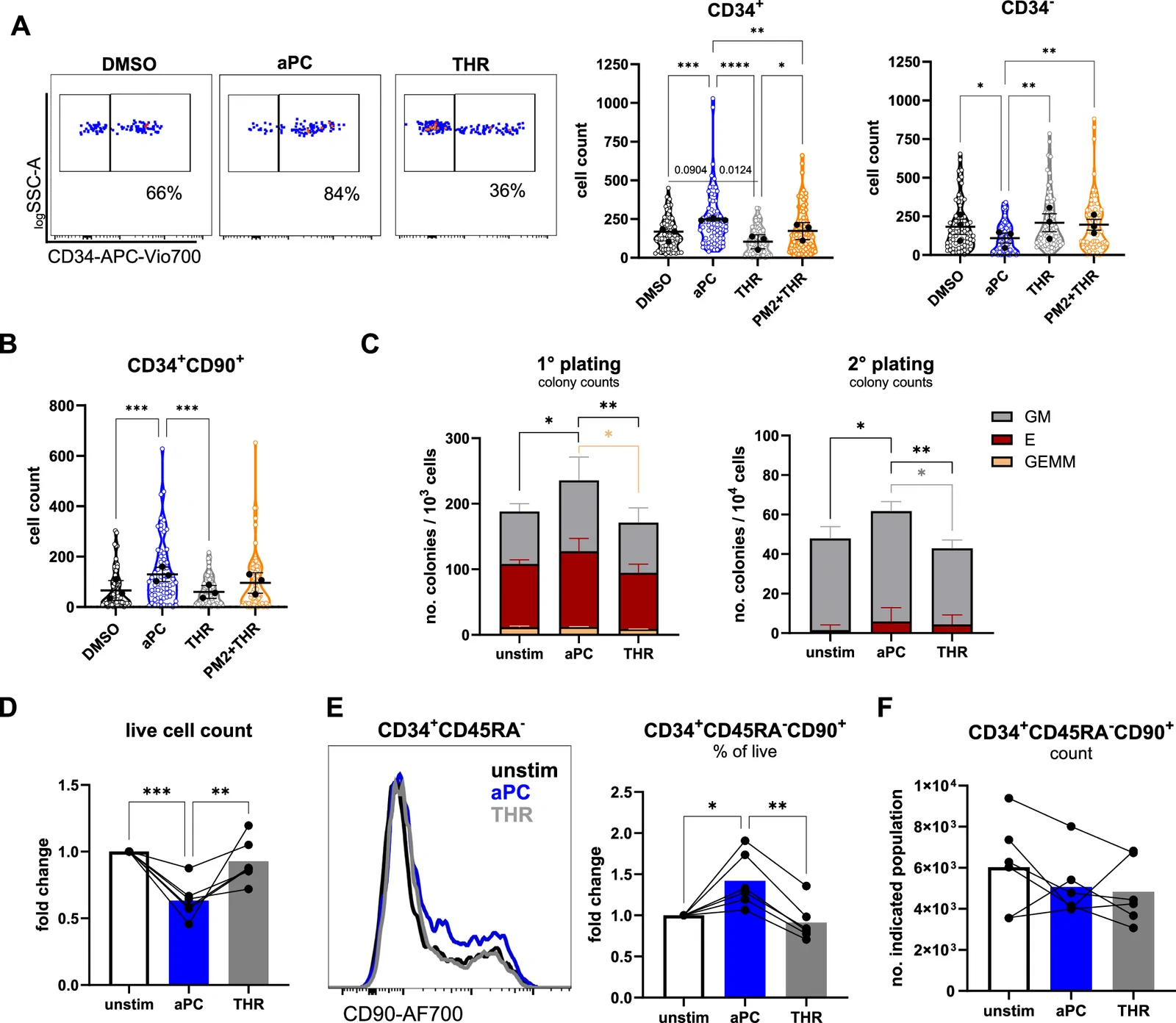
aPC slows down differentiation and increases colony-forming potential in vitro. (**A**) Single-cell expansion assay of CD34^+^ cells stimulated for 1 h with DMSO (0.1%), aPC (10 µg/ml), THR (1 U/ml) or PM2 (10 µM) + THR (1 U/ml); stimulated cells were single cell sorted and cultivated without stimuli for 7 days, representative flow analysis of colonies and cell counts of CD34^+^ and CD34^−^ cells per colony are shown, DMSO *n* = 67, aPC *n* = 66, THR *n* = 67, PM2 + THR *n* = 68 colonies from three independent experiments with 1 mPB donor. (**B**) Cell counts of CD34^+^CD90^+^ cells of each colony after 7 days from a single-cell expansion assay, DMSO *n* = 69, aPC *n* = 68, THR *n* = 66, PM2 + THR *n* = 70 colonies from three independent experiments from 1 mPB donor. (**C**) Colony-forming unit (CFU) assays of CD34^+^ cells cultivated for 2 days and then stimulated for 1 h with aPC (10 µg/ml), THR (1 U/ml), or left unstimulated; number of erythroid (E), granulocyte-macrophage (GM) and granulocyte-erythroid, macrophage, megakaryocyte (GEMM) colonies in primary and secondary platings are shown, *n* = 3 mPB donors. (**D**) CD34^+^ cells were cultivated for 7 days in normal HSC medium (unstim) or medium supplemented with aPC (10 µg/ml) or THR (1 U/ml), shown is the fold change of live cell count to normal medium, *n* = 6 mPB donors. (**E**) Representative expression levels of HSC marker CD90 and fold change of frequency of CD34^+^CD45RA^−^CD90^+^ cells of all live cells after 7 days with aPC (10 µg/ml), THR (1 U/ml), or normal medium are shown, *n* = 6 mPB donors. (**F**) Total count of CD34^+^CD45RA^−^CD90^+^ cells in cultures after 7 days of continuous aPC or THR stimulation starting from 5000 input CD34^+^ cells, *n* = 6 mPB donors. Statistical analysis: (**A**,** B**) Colored dots and violin plots indicate outcomes from every colony, thick filled black dots indicate the mean of each independent experiment with black line and error bars indicating mean ± SD, numerical *p* values indicate significance of the means, stars indicate significance when all colonies are compared. **C** colored comparison depicts difference in one colony type, black comparison indicates comparison between total colony count. (**D**–**F**) The bar height represents the mean; lines connect each individual donor. (**A–F**) One-way ANOVA with Tukey’s multiple comparison test, *P ≤ 0.05, **P ≤ 0.01, ***P ≤ 0.001, ****P ≤ 0.0001, exact *P* values can be found in Appendix Table S6. Source data are available online for this figure.

**Figure EV6:**
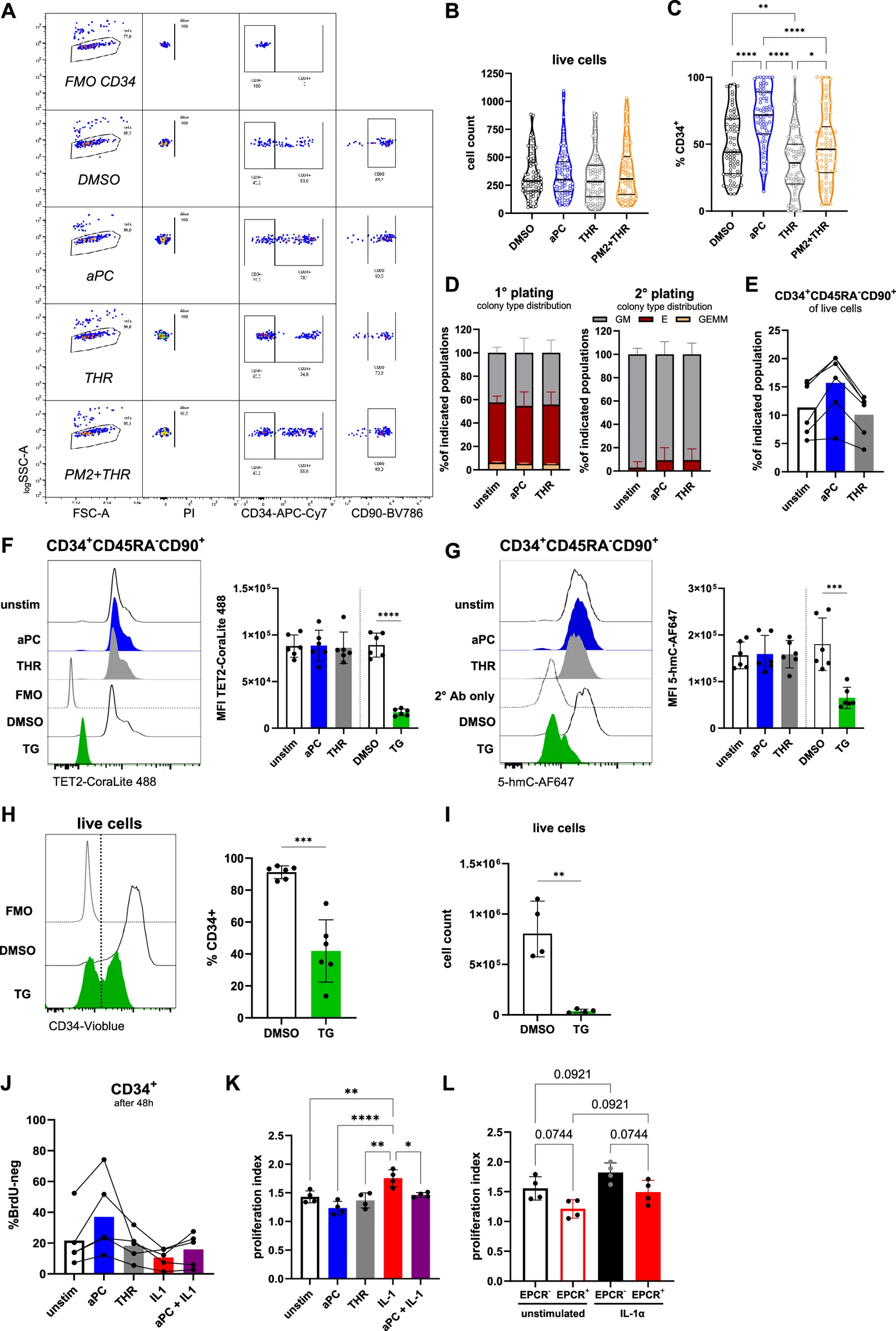
In vitro culture and colony formation after stimulation with aPC or THR. (**A**) Representative FC gating for analysis of colonies from the single-cell expansion assay. (**B**) Colony size (live cells per colony) and (**C**) frequency of CD34^+^ cells in colonies after 7 days from single-cell expansion assay, *n* = 69–71 colonies from three independent experiments, 1 mPB donor. (**D**) Colony type distribution of CD34^+^ cells in primary and secondary plating of colony-forming unit (CFU) assay, CD34^+^ cells were cultivated for 2 days prior to stimulation with aPC (10 µg/ml) or THR (1 U/ml) for 1 h before primary plating, *n* = 3 mPB donors. (**E**) Frequency of HSC-enriched CD34^+^CD45RA^−^CD90^+^ cells within all live cells after 7 days, *n* = 6 mPB donors. (**F**) TET2 expression and (**G**) 5-hydroxymethylcytosine (5-hmC) levels in CD34^+^CD45RA^−^CD90^+^ cells after 7 days of cultivation with normal HSC medium (unstim), aPC (10 µg/ml), THR (1 U/ml), DMSO (0.1%), or Thapsigargin (TG, 1 µM), *n* = 6 mPB donors. (**H**) Frequency of CD34^+^ cells after 7 days of cultivation with DMSO (0.1%) or TG (1 µM), *n* = 6 mPB donors. (**I**) number of live cells per well after 7 days of cultivation, starting from 7.5 × 10^4^ cells, *n* = 4 mPB donors. (**J**) Bar chart showing incorporation of BrdU into the genome of CD34^+^ cells after 2 days in culture with the following stimuli: aPC (10 µg/ml), THR (1 U/ml), Il-1α (10 ng/ml), or aPC (10 µg/ml) and IL-1α (10 ng/ml) or unstimulated, *n *= 4 mPB donors. (**K**) proliferation indices of CD34^+^CD45RA^−^CD90^+^ after 3 days in culture with the following stimuli: aPC (10 µg/ml), THR (1 U/ml), Il-1α (10 ng/ml) or aPC (10 µg/ml), and IL-1α (10 ng/ml) or unstimulated, *n* = 4 mPB donors. (**L**) proliferation indices of CD34^+^CD45RA^−^CD90^+^EPCR^+/−^ after 3 days in culture with or without Il-1α (10 ng/ml), *n* = 4 mPB donors. Statistical analysis: (**B**,** C**) thick line indicates median, thin line indicates interquartile. (**D**,** F**–**I**,** K**,** L**) Data presented as mean ± SD. (**E**,** J**) Bar height represents the mean, lines connect samples from individual donors. (**B**–**E**,** J**,** K**) One-way ANOVA with Tukey’s multiple comparison test. (**F**,** G**) Unstim vs. aPC vs. THR: one-way Anova with Tukey’s multiple comparison test, DMSO vs. TG: unpaired two-tailed *t*-test, (**H**,** I**) unpaired two-tailed *t*-test, (**L**) two-way Anova with Holm–Šídák’s multiple comparison test; *P ≤ 0.05, **P ≤ 0.01, ***P ≤ 0.001, ****P ≤ 0.0001, exact *P* values can be found in Appendix Table S13.

Gene expression analysis indicated a lower proliferation rate in aPC-stimulated CD34^+^ cells, and we now addressed whether this also led to altered HSPC expansion in vitro. Therefore, we conducted single-cell expansion assays with CD34^+^ cells stimulated for 1 h with aPC, THR, or PM2 and THR and then sorted into individual wells. After 7 days, aPC-stimulated cells gave rise to colonies with higher numbers of CD34^+^ cells (Fig. 5A) and HSC-enriched CD34^+^CD90^+^ cells (Fig. 5B and EV6A) without a change in total cell count per colony (Fig. EV6B). Consequently, the percentage of CD34^+^ cells in the colonies increased with aPC treatment (Fig. EV6C), while THR had the opposite effect, leading to increased differentiation of CD34^+^ cells (Fig. EV6C). PM2 prevented this effect in part by increasing the contribution and absolute number of CD34^+^ cells back to levels of control (Figs. 5A and EV6C). aPC stimulation also increased colony numbers in colony-forming-unit assays (Fig. 5C). Upon secondary plating, aPC-stimulated cells trended toward higher colony counts, whereas THR-treated cells produced significantly fewer colonies (Fig. 5C), but the overall colony type distribution both in primary and secondary plating was not changed among treatments (Fig. EV6D). Continuous PAR1 activation with aPC over 7 days of cultivation of CD34^+^ cells reduced the total number of cells but increased the contribution of CD34^+^CD45RA^−^CD90^+^ cells, resulting in unchanged absolute counts of CD34^+^CD45RA^−^CD90^+^ cells. (Figs. 5D–F and EV6E).

Since HSC differentiation is concomitant with DNA demethylation (Hogart et al, 2012; Farlik et al, 2016), we investigated whether aPC and THR also changed levels of methyl cytosine dioxygenase TET2, resulting in DNA demethylation as a potential explanation for decreased/increased differentiation. Reduced 5-methyl cytosine levels have been described in aPC-stimulated T cells (Gupta et al, 2023), and THR may change TET2 levels due to elevated intracellular Ca^2+^ levels (Luchsinger et al, 2019). We did, however, not observe any changes in TET2 and 5-hydroxymethylcytosine (5-hmC) levels in CD34^+^CD45RA^−^CD90^+^ cells after 1 week of continuous aPC or THR treatment (Fig. EV6F,G). Since Thapsigargin also increased cytosolic Ca^2+^ levels (Fig. EV2I,J), we used it as a positive control and could observe a significant decrease in TET2 levels (Fig. EV6F) and reduced 5-hmC (Fig. EV6G). This resulted in loss of CD34^+^ cells (Fig. EV6H) and also blocked cell proliferation (Fig. EV6I). In summary, aPC promoted clonogenic potential and maintained undifferentiated hematopoietic cells in vitro.

### aPC counteracts interleukin 1α (IL-1α)-induced proliferation of CD34^+^ HSPCs in vitro

**Figure 6:**
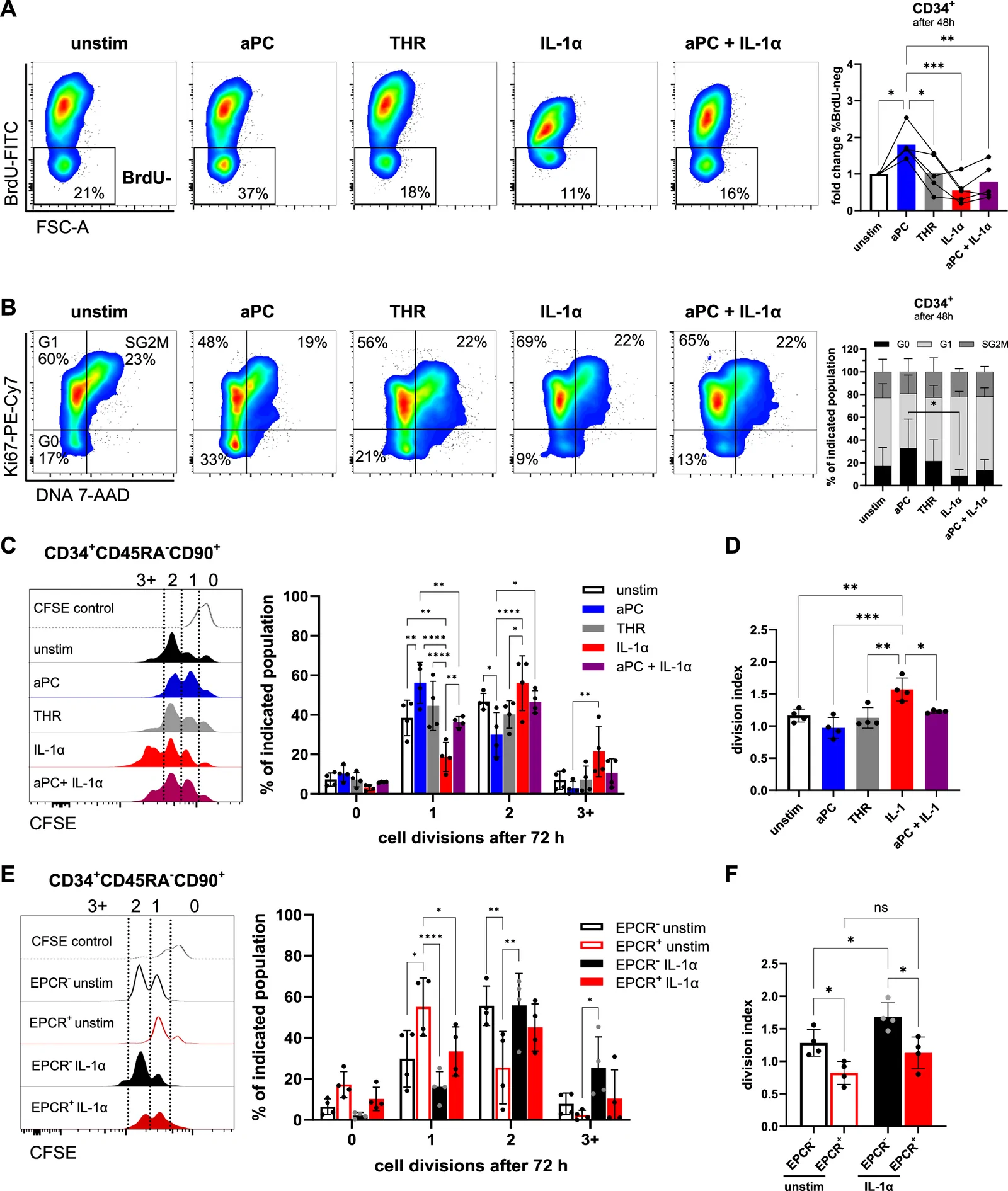
aPC and EPCR protect CD34^+^ cells from cell cycle activation in vitro. (**A**) Representative FC plots depicting BrdU incorporation and fold change of percentage of BrdU-negative cells after 48 h and (**B**) cell cycle analysis by Ki67 and 7-AAD of CD34^+^ cells after 48 h of stimulation with aPC (10 µg/ml), THR (1 U/ml), IL-1α (10 ng/ml), aPC (10 µg/ml) +IL-1α (10 ng/ml) or normal medium (unstim), *n* = 5 mPB donors. (**C**) Cell divisions of CD34^+^CD45RA^−^CD90^+^ cells within CD34^+^ bulk were assessed by CFSE dye dilution after 72 h of stimulation with aPC (10 µg/ml), THR (1 U/ml), IL-1α (10 ng/ml), aPC (10 µg/ml) +IL-1α (10 ng/ml) or normal medium (unstim), CFSE control is an unstimulated control recorded after 24 h to mark starting CFSE signal. Dotted vertical lines indicate cell divisions, *n* = 4 mPB donors. (**D**) Division indices of samples from (**C**), *n* = 4 mPB donors. (**E**) Cell divisions of CD34^+^CD45RA^−^CD90^+^EPCR^+/−^ cells were assessed by CFSE dye dilution after 72 h of stimulation with IL-1α (10 ng/ml) or unstimulated cells, CFSE control is an unstimulated control recorded after 24 h to mark starting CFSE signal, dotted vertical lines indicate cell divisions, *n* = 4 mPB donors. (**F**) Division indices of samples and populations from **E**), *n* = 4 mPB donors. Statistical analysis: (A) Connected dots indicate each donor, bar height represents the mean. (**B**–**F**) Data depicted as mean ± SD. (**A**,** B**) Numbers in FC plots indicate the mean of all donors. (**A**,** D**) One-way ANOVA with Tukey’s multiple comparison test. (**C**,** E**,** F**) Two-way Anova with Holm–Šídák’s multiple comparison test; ns not significant, *P ≤ 0.05, **P ≤ 0.01, ***P ≤ 0.001, ****P ≤ 0.0001, exact *P* values can be found in Appendix Table S7. Source data are available online for this figure.

In mouse models, EPCR^+^ HSCs have been shown to be protected from activation by inflammatory cytokines (Rommel et al, 2022; Rabe et al, 2020); however, it is unclear if aPC can also play a role in protecting human CD34^+^ cells in vitro. We investigated whether the stimulation of CD34^+^ cells with aPC alters their proliferation and whether it prevents IL1a-induced HSC activation (Pietras et al, 2016; Carey et al, 2017). Freshly thawed CD34^+^ cells cultivated with aPC showed a delay in the first cell division determined by BrdU incorporation after 48 h (Figs. 6A and EV6J). Consequently, with aPC in the culture medium, more CD34^+^ cells resided still in G0, defined by absence of Ki67 after 48 h, compared to cells stimulated with IL-1α (Fig. 6B). Cell division counting via CFSE dye dilution showed that within 72 h, CD34^+^CD45RA^−^CD90^+^ cells underwent fewer cell divisions in the presence of aPC, whereas addition of IL-1α accelerated cell divisions (Fig. 6C,D). Notably, a 4 h pretreatment with aPC fully prevented the IL-1α-driven proliferation (Fig. 6C). Both the division index (Fig. 6D) and the proliferation index (Fig. EV6K) were elevated with IL-1α but normalized to baseline when cells were pretreated with aPC. In contrast, THR altered neither BrdU incorporation (Fig. 6A) nor cell cycle status of CD34^+^ cells after 48 h (Fig. 6B), nor division kinetics (Fig. 6C,D). Within the CD34^+^CD45RA^−^CD90^+^ population, EPCR^+^ cells divided slower than EPCR^-^, also under IL-1α stimulation, indicating an intrinsic protection of human EPCR^+^ HSPC against activation by this inflammatory cytokine also in vitro (Figs. 6E,F and EV6L).

### aPC increases human hematopoietic cell contribution in NBSGW mice

**Figure 7:**
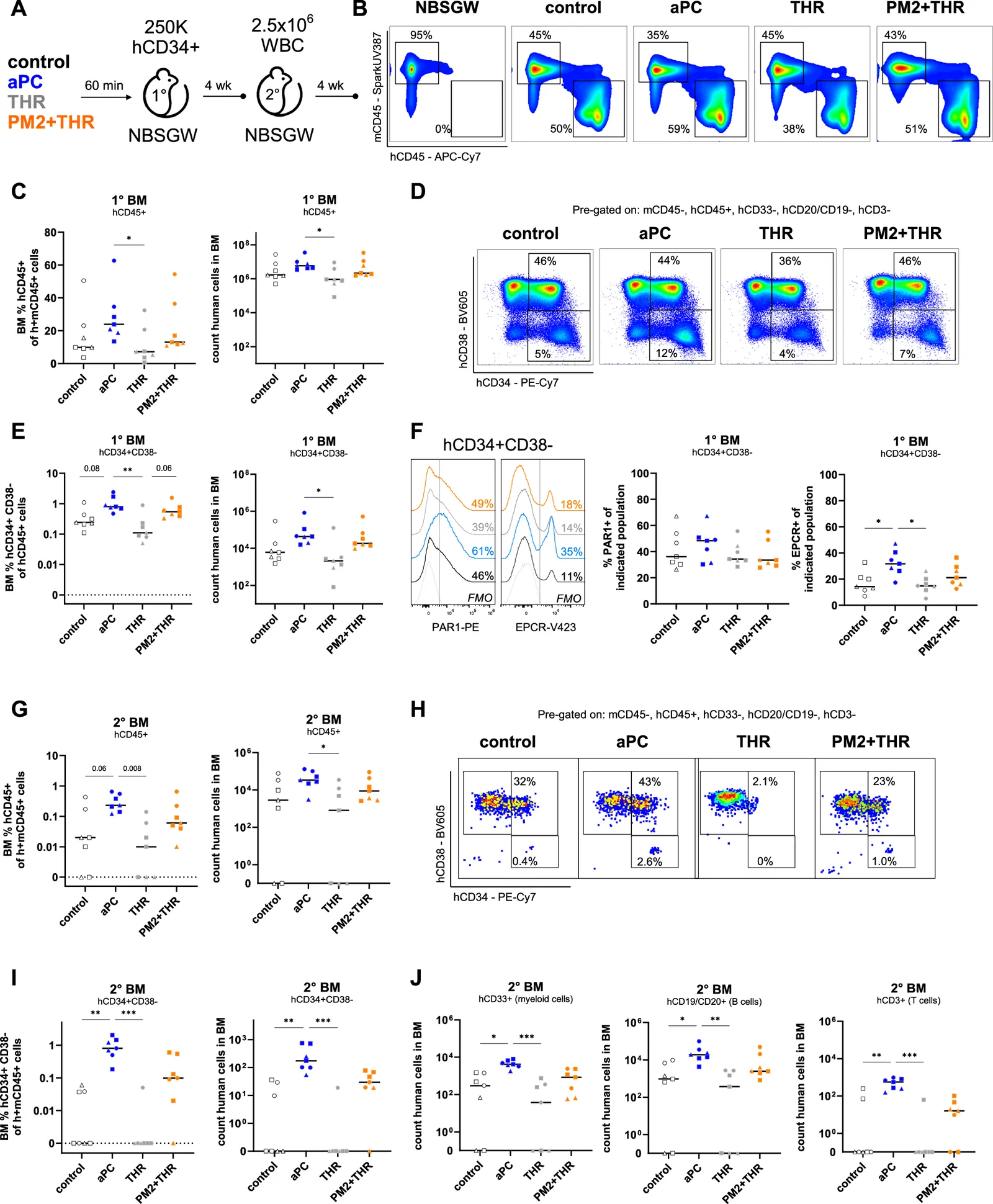
In vitro aPC stimulation of CD34^+^ cells enhances short-term human engraftment in NBSGW recipients. (**A**) Primary (1°) and secondary (2°) human mPB CD34^+^ xenograft transplantation model in NBSGW mice. Primary transplantation of hCD34^+^ cells stimulated for 60 min with aPC (10 µg/mL), THR (1 U/mL), and PM2 (10 µM) + THR (1 U/mL) or control (0.1% DMSO). 2.5 × 10^5^ CD34^+^ cells were transplanted primarily into unconditioned NBSGW recipient mice. Peripheral blood and BM analysis 4 weeks post-transplantation. 2.5 × 10^6^ WBCs from the bone marrow of 1° transplanted NBSGW recipient mice were transplanted secondary (2°) into unconditioned NBSGW recipient mice. BM analysis 4 weeks post-transplantation. (**B**) Representative FC plots showing composition of murine (m) and human (h) CD45^+^ cells in the BM of 1° NBSGW mice 4 weeks post-transplantation for all conditions. (**C**) Frequency and count of human CD45^+^ cells in murine BM of 1° transplanted NBSGW mice. aPC, THR, PM2 + THR and control (DMSO), *n* = 7 biological replicates per treatment from three independent experiments. Different symbol shapes indicate three independent performed stimulations and transplantations. (**D**) Representative FC plots showing composition of human immature hCD45^+^hCD33^−^, hCD19/20^−^ hCD3^−^ cells in the BM of 1° NBSGW mice 4 weeks post-transplantation for all conditions. (**E**) Frequency within human CD45^+^ cells and absolute count of human CD34^+^CD38^−^ cells in the BM. Dotted line indicates human engraftment below 0.001% = 0%. (**F**) Cell surface expression of PAR1 and EPCR on CD34^+^CD38^−^ cells in the BM of 1° transplanted NBSGW mice after 4 weeks. (**G**) Percentage and count of human CD45^+^ cells in murine BM of 2° WBC transplanted NBSGW mice. aPC, THR, PM2 + THR and control (DMSO), *n* = 7 biological replicates per treatment from three independent experiments, different symbol shapes indicate three independent stimulations and transplantations. Dotted line indicates human engraftment below 0.001% =  0%. (**H**) Representative FC plots showing composition of human immature hCD45^+^hCD33^−^, hCD19/20^−^ hCD3^−^ cells in the BM of 2° NBSGW mice 4 weeks post-transplantation for all conditions. (**I**) Frequency within human CD45^+^ cells and count of human CD34^+^CD38^−^ of 2° NBSGW mice. Dotted line indicates human engraftment below 0.001% = 0%. (**J**) Count of human CD33^+^ (myeloid), CD19/CD20^+^ (B cells), and CD3^+^ (T cells) in the BM of 2° transplanted NBSGW mice. Statistical analysis: All data represented as median, non-parametric one-way ANOVA with Kruskal–Wallis test and Dunn’s multiple comparison test; *P < 0.05, **P < 0.01, ***P < 0.001, exact *P* values can be found in Appendix Table S8. Source data are available online for this figure.

We hypothesized that aPC treatment would support engraftment of human CD34^+^ cells in NBSGW mice by increasing stemness and preventing differentiation, as seen in our in vitro experiments. We therefore stimulated CD34^+^ cells with aPC, THR or PM2 + THR for 1 h and transplanted 2.5 × 10^5^ stimulated or control CD34^+^ cells per mouse (Fig. 7A). Four weeks after transplantation, there was a trend of more human CD45^+^ cells in the BM (both in frequency and absolute count) in mice transplanted with aPC-treated cells (Fig. 7B,C; Appendix Fig. S2C). THR-treated cells showed a trend towards lower engraftment. Most effects of PAR1 activation were apparent on immature CD34^+^CD38^−^ cells: aPC increased the median number of CD34^+^CD38^−^ in the BM sevenfold, whereas THR stimulation caused a 65% reduction of median human CD34^+^CD38^−^ cell number (Fig. 7D,E). PM2 pretreatment prevented THR-mediated reduction of human BM engraftment (Fig. 7C) and tripled the median number of CD34^+^CD38^−^ cells compared to control (Fig. 7D,E), probably associated with its ability to bias THR/PAR1 signaling towards aPC/PAR1 signaling. Interestingly, mice transplanted with aPC-stimulated cells had an increased proportion of EPCR-positive cells within the CD34^+^CD38^−^ population (Fig. 7F). PAR1+ levels in CD34+CD38− cells from the BM were not altered between groups and only around 40–50% in vivo (Fig. 7F), which was considerably lower than on the freshly thawed mPB samples (close to 100%, Fig. 1A).

**Figure EV7:**
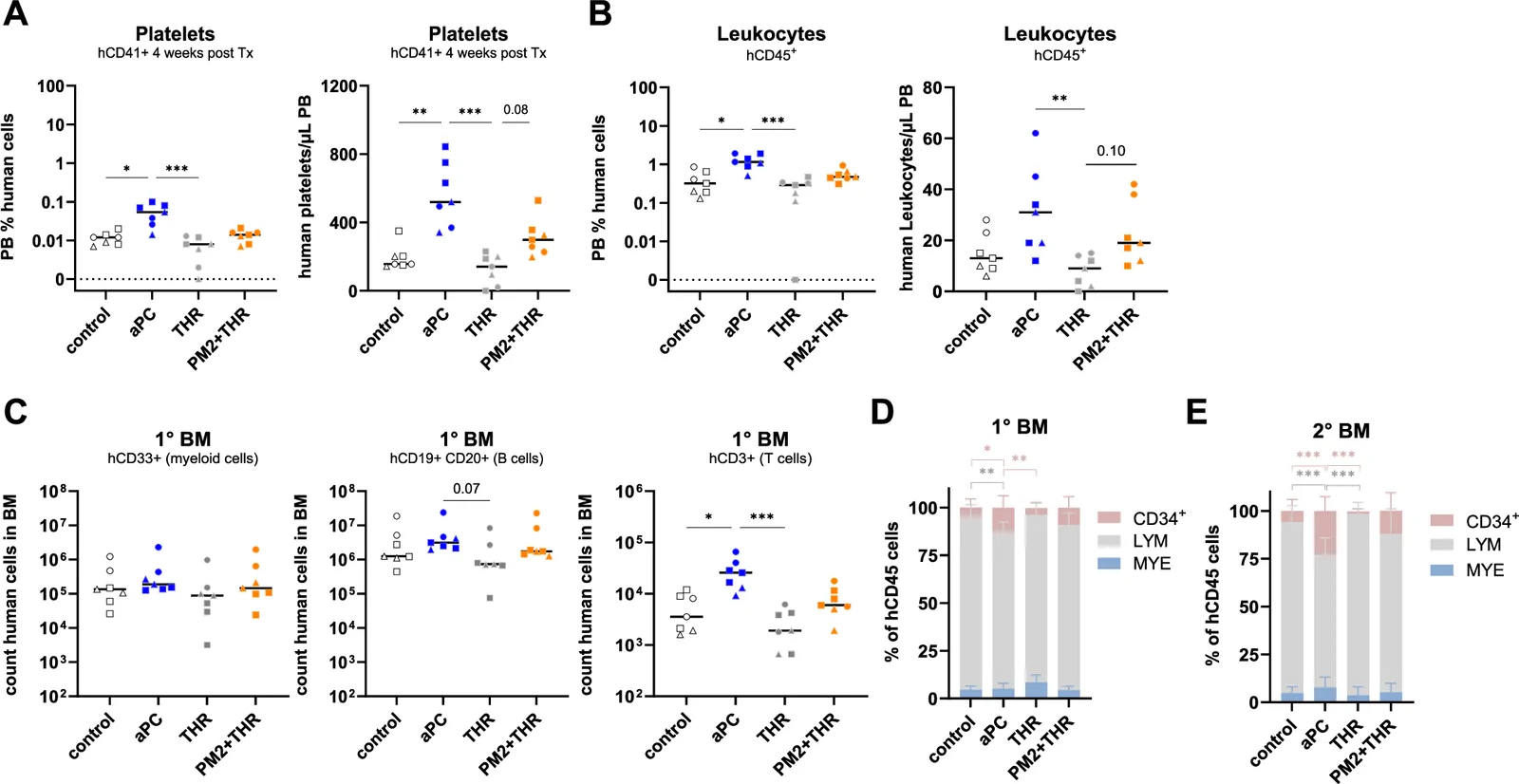
Peripheral blood and multilineage engraftment of human cells in NBSGW mice. (**A**) Frequency and cell counts/µl of human platelets (hCD41^+^) are shown 4 weeks after transplantation of stimulated CD34^+^ cells into NBSGW mice. aPC (*n* = 7), THR (*n* = 7), PM2 + THR (*n* = 7), and control (DMSO, *n* = 7). Different symbol shapes indicate three independent performed stimulations and transplantations. (**B**) Frequency and cell counts/µl of human leukocytes (hCD45^+^) are shown 4 weeks after transplantation of stimulated CD34^+^ cells into NBSGW mice. (**C**) Counts of human CD33^+^ (myeloid), CD19/CD20^+^ (B cells), hCD3^+^ (T cells) in the BM of transplanted NBSGW mice after 4 weeks. (**D**) relative contribution of CD34^+^ , myeloid (MYE) and lymphoid (LYM, B + T cells) in BM of 1° and (**E**) 2° transplanted NBSGW mice after 4 weeks. Statistical analysis: (**A**–**C**) Data represented as median, non-parametric one-way-ANOVA with Kruskal–Wallis test. (**D**,** E**) Data represented as mean ± SD, two-way Anova with Holm–Šídák’s multiple comparison test, only selected comparisons are shown; *P ≤ 0.05, **P ≤ 0.01, ***P ≤ 0.001, ****P ≤ 0.0001, exact *P* values can be found in Appendix Table S14.

In accordance with other studies (Choo et al, 2024; Hess et al, 2020; Rahmig et al, 2016), we detected low levels of peripheral blood chimerism in this mouse model, but also here, we observed a significant increase in human platelets and leukocytes in mice transplanted with aPC-stimulated CD34^+^ cells (Fig. EV7A,B; Appendix Fig. S2A,B). Due to the low levels of human peripheral blood cells, we analysed multilineage differentiation capacity in the BM of the transplanted mice. Counts and relative contribution of myeloid cells (hCD33^+^) were similar between all groups, however more B cells (hCD19/CD20^+^) were present in mice transplanted with aPC-treated CD34^+^ cells compared to the THR group. T cells (hCD3^+^) were also more abundant in mice transplanted with aPC-treated CD34^+^ cells, compared to control and THR-treated cells (Fig. EV7C,D).

To prove that aPC treatment supported short-term serial repopulation potential of CD34^+^ cells, we performed secondary transplantations using whole BM from primary recipients one month after their transplantation. All secondary recipients that received aPC or PM2 + THR-stimulated cells showed human engraftment in the BM after 4 weeks, but only 4/7 mice which received THR-stimulated cells. (Fig. 7G). Following our observations in primary recipients, human CD34^+^CD38^−^ cells were much more abundant in the BM after aPC treatment (7/7 mice engrafted CD34^+^CD38^−^ cells), and THR diminished secondary engraftment of CD34^+^CD38^−^ cells (Fig. 7H,I). Similarly, aPC treatment led to multilineage humanization of all (7/7) secondary recipients, in stark contrast to THR-stimulated or control cells. In these groups, human T cells were only detectable in 1/7 (THR) and 2/7 (control) mice (Fig. 7J). PM2 + THR improved short-term multilineage differentiation capacity, leading to myeloid, B and T cell development in 5/7 mice (Fig. 7J). The relative contribution of myeloid cells was not altered in the BM between groups; however, in the aPC group, CD34^+^ cells were more abundant (Fig. EV7E). Taken together, the opposing effects of aPC and THR on CD34^+^ HSPCs influenced their short-term serial engraftment potential, where a single dose of aPC maintained CD34^+^CD38^−^ cells and enabled short-term serial multilineage differentiation capacity.

## Discussion

In our study, we describe the divergent signals and biological function mediated by aPC- or THR-induced activation of PAR1 in CD34^+^ HSPC. Although EPCR, the coreceptor of PAR1, is a well-known HSC marker, our understanding of its functional role in HSPCs is less well understood, and our study therefore contributes important insights.

Our data support a model in which THR-induced PAR1 signaling leads to enhanced differentiation of HSPCs at the cost of maintaining HSPCs, including phenotypic HSCs. On the molecular levels, THR induced ERK1/2 phosphorylation and Ca^2+^ flux in CD34^+^ cell populations, and both signals have been linked to hematopoietic differentiation and migration/mobilization (Wang et al, 2021; Li et al, 2025; Henschler et al, 2003). The Ca^2+^ flux increases cytosolic Ca^2+^ levels, which will also elevate mitochondrial Ca^2+^ concentration (Hajnóczky et al, 1995; Collins et al, 2001), thereby increasing mitochondrial activity (Robb-Gaspers et al, 1998; Vilas-Boas et al, 2023). This Ca^2^^+^-mitochondria axis was linked in HSCs to increase cell division and differentiation (Umemoto et al, 2018). In our experiments, THR increased glycolysis in CD34^+^ cells, which at first glance may not be harmful for HSC function, since they normally rely on glycolysis during quiescence (Morganti et al, 2022). On the other hand, increased levels of glycolysis produce cytotoxic methylglyoxal (Allaman et al, 2015), which accumulated in CD34^+^ cells after THR stimulation, potentially contributing to decreased HSPC potential observed in our study.

In contrast, AKT phosphorylation was much stronger after stimulation with aPC than with THR. AKT activation has been shown to be induced in vitro in cytokine-containing media, as freshly isolated HSCs show no AKT activity (Yamazaki et al, 2006), and to be beneficial for HSC survival in culture (Sakurai et al, 2023). We further detected activation of CREB, an important transcription factor also downstream of TPO/MPL signaling in HSCs (Tsutsumi et al, 2023), following stimulation with both aPC and THR, pointing to shared pathways activated downstream of PAR1 by both activators.

Importantly, aPC treatment counteracted IL-1-induced proliferation of human CD34^+^CD45RA^−^CD90^+^ cells in vitro. In mouse studies, it was reported that acute or chronic exposure to IL-1 led to myeloid priming and eventually hematopoietic impairment (Pietras et al, 2016; Chavez et al, 2022). Elevated IL-1 levels in the BM are associated with aging, during which, interestingly, the contribution of EPCR^+^ HSC increases (Kovtonyuk et al, 2022; Lin and Trumpp, 2023). This increase may be explained by the selective protection of EPCR^+^ HSC in mice, which were maintained in quiescence during viral infection or repeated IL-1 application (Rommel et al, 2022; Rabe et al, 2020). In this respect, our observation that aPC treatment prevents IL-1-induced cell-cycle activation in vitro also in human HSPC points to a similar role of aPC/EPCR/PAR1 signaling in human cells under inflammatory stress.

Most strikingly, we observed that a brief 1-hour in vitro stimulation of CD34^+^ cells with aPC supported serial short-term human cell engraftment in NBSGW mice, with better multilineage engraftment and higher numbers of CD34^+^CD38^−^ HSPCs, considered to be phenotypical repopulating HSC (Bhatia et al, 1997). We did not analyze long-term engraftment in NBSGW mice, which is a major limitation of our study. However, secondary transplantation selectively measures HSCs with self-renewal and multilineage reconstitution capacity (Wiekmeijer et al, 2014; Park et al, 2008). Indeed, the vast majority of human MPPs and committed progenitors do not persist beyond early timepoints and fail to contribute to secondary engraftment in xenografts (Notta et al, 2011; Notta et al, 2016; Majeti et al, 2007).

Our findings are highly relevant for improving HSC transplantation outcomes by ensuring more robust hematopoietic recovery. As shown by Gur-Cohen et al (Gur-Cohen et al, 2015), aPC increases migration of murine HSPCs towards CXCL-12 and bone marrow retention, a mechanism which may contribute to the increased engraftment we observed. Although homing was not directly addressed in our study, our transcriptome analysis of aPC-treated CD34^+ ^cells, revealing a significant enrichment in pathways regulating migration and cell adhesion, supports the notion that the findings from mouse models also apply to human cells.

EPCR expression on non-cultivated human HSCs (Fares et al, 2017; Anjos-Afonso et al, 2022; Subramaniam et al, 2019) is very low, and others have argued that human CD34^+^ cells do not express thrombomodulin, necessary to convert protein C to aPC in situ (Basu et al, 2020), questioning the role of aPC/EPCR/PAR1 in human HSCs. In human and murine blood, free aPC levels are below 1–2 ng/ml (Dempfle et al, 2011; Pepler et al, 2015; Zheng et al, 2007), but higher local concentrations can be expected, e.g., in the BM after conversion of protein C to aPC on EPCR^+^ BM endothelial cells or EPCR^+^ HSCs (Gur-Cohen et al, 2015). EPCR^+^ endothelial BM cells have recently been shown to maintain human HSPC function via NOTCH3 signaling on mesenchymal stem cells when stimulated with aPC, supporting the notion that aPC/EPCR/PAR1 signaling is relevant in maintaining HSPC function within the BM niche (Xu et al, 2026). The superior engraftment of EPCR^+^ human HSC argues strongly for an important function in human HSC (Anjos-Afonso et al, 2022). Physiologically low THR concentrations can also be expected to be locally elevated in the BM during trauma. THR/PAR1 was implicated in driving the bone repair process following fractures (Song et al, 2005) by activating osteoblasts and increasing IL-6 production (Pagel et al, 2009). Studies investigating whether elevated THR levels due to (thrombo-) inflammation or bleeding also directly activate HSCs in vivo are, however, lacking.

In our experiments, aPC acted largely independently of EPCR on PAR1 to maintain quiescence of CD34^+^ HSPCs in vitro, partly because of the high aPC concentration. In vitro, HSCs lose quiescence after 24–72 h (Shin et al, 2020; Hammond et al, 2023; Johnson et al, 2024), and HSCs that remain non-cycling ex vivo have been shown to engraft more effectively (Glimm et al, 2000; Jetmore et al, 2002). EPCR-independent aPC/PAR1 signaling could therefore overcome the low EPCR expression found on human HSCs from apheresis material and bone marrow aspirate, both of which are clinically relevant sources for HSC transplantations.

Pharmacological modulation of PAR1 using PM2 supports the functional relevance of biased PAR1 signaling in human CD34^+^ HSPCs. However, the extent to which PM2 recapitulates aPC-like signaling is questionable, since we did not observe AKT activation as reported in endothelial cells (de Ceunynck et al, 2018). There might be further signaling events downstream of PAR1 that remain unaltered by PM2 in CD34^+^ cells, such as G_α12/13_-mediated RhoA activation by THR (Aisiku et al, 2015; Künze & Isermann, 2023), which we did not investigate here. Nonetheless, PM2 was able to rescue CD34^+^ HSPCs from THR-mediated reduction of short-term engraftment and this effect was associated with abrogation of THR-induced ERK1/2 and CREB phosphorylation and Ca^2+^ flux. Our findings highlight a previously underappreciated fine-tuning of PAR1 signaling in CD34^+^ HSPCs by using small molecule modulators, which may be used to support hematopoietic potential.

Although we cannot causally link the observed PAR1 downstream signaling events following aPC and THR stimulation to the functional changes in CD34^+^ HSPCs, our study characterizes which PAR1 signaling pathways are activated in human HSPCs in vitro and provides evidence for a supportive role of aPC treatment of CD34^+^ cells in their short-term engraftment in mice. This could be followed up in future studies using approaches that can selectively uncouple individual PAR1 signals from downstream effectors, to further define the role of EPCR/PAR1 signaling in HSC maintenance or activation.

Overcoming low levels of HSCs engraftment after transplantation remains a major clinical need and would provide significant benefits for patients, particularly in therapies that rely on ex vivo culture and genetic modification of HSCs. We demonstrate that PAR1 is a druggable target in human CD34^+^ HSPCs in vitro, and that its biased activation by aPC offers a promising strategy to improve HSC engraftment.

## Methods

**Table:** Reagents and tools table

Reagent/resource	Reference or source	Identifier or catalog number
**Experimental models**		
NBSGW	Jackson Laboratory	NOD.Cg-*Kit*^*W-41J*^ *Tyr* ^*+*^ *Prkdc*^*scid*^ *Il2rg*^*tm1Wjl*^/ThomJ
CD34-positive enriched human cells (mobilized peripheral blood)	BioIVT	HUMANCD34-0132289
CD34-positive enriched human cells (mobilized peripheral blood)	Lonza	4Y-101C
CD34-positive enriched human cells (mobilized peripheral blood)	Cyto-Care GmbH as mobilized apheresis bag, enriched by Somagenetix AG	
CD34-positive enriched human cells (bone marrow aspirate)	BioIVT	HUMANCD34-0122753
**Recombinant DNA**		
NA		
**Antibodies**		
Anti-human CD34-Vioblue	Miltenyi	130-113-182
Anti-human CD38-BV605	BD Bioscience	562665
Anti-human CD45RA-BV786	BD Bioscience	563870
Anti-human CD90-Alexa Fluor 700	BioLegend	328120
Anti-human EPCR-APC (clone RCR-401)	BioLegend	351906
Anti-human EPCR-APC (clone RCR-252)	BD Bioscience	563622
Anti-human EPCR-PE-Vio 770	Miltenyi	130-122-978
Anti-human Thrombin-Receptor-PE (PAR1-PE, clone WEDE15)	Beckman Coulter	IM2584
Anti-human Thrombin-Receptor-PE (PAR1-PE, clone SPAN12)	Beckman Coulter	IM2583
Anti-human CD34-APC-Vio700	Miltenyi Biotec	130-124-457
Anti-human CD90-BV785	BioLegend	328142
Anti-pAKT-Alexa Fluor 647	BD Bioscience	560343
Anti-pERK1/2- Alexa Fluor 647	BD Bioscience	612593
Anti-pNF-kB- Alexa Fluor 647	BD Bioscience	558422
Anti-pCREB- Alexa Fluor 647	Cell Signaling technology	14001
Anti-human CD34-PE-Cy7	Biolegend	343516
Anti-Ki67-PE-Cy7	eBioscience	25-5698-82
Anti-BrdU-FITC	eBioscience	11-5071-42
Anti-human TET2-CoraLite 488	Proteintech	CL488-21207-100UL
Rabbit anti-5-hmC	Active Motif	39092
Goat anti-rabbit- Alexa Fluor 647	invitrogen	A21244
Anti-Methylglyoxal-FITC	Sigma-Aldrich	SAB5202574-100UG
Anti-mouse CD45-SparkUV 387	BioLegend	103188
Anti-mouse Ter119-PE-Cy5	BioLegend	116210
Anti-human CD19-FITC	BioLegend	392507
Anti-human CD20-FITC	BioLegend	302303
Anti-human CD33-APC	BioLegend	303407
Anti-human CD34-PE-Cy7	BD Bioscience	560710
Anti-human CD45-APC-Cy7	BioLegend	304014
Anti-human CD3-BV510	BioLegend	317332
Anti-human CD38-BV605	BioLegend	356641
Anti-mouse CD61-Alexa Fluor 647	BioLegend	133934
Anti-human CD45-PE Dazzle 594	BioLegend	304052
Anti-human CD41-BV421	BioLegend	303730
Anti-human CD38	BD Bioscience	940466
Anti-human CD45RA	BD Bioscience	940011
Anti-human CD44	BD Bioscience	940039
Anti-human CD10	BD Bioscience	940045
Anti-human CD123	BD Bioscience	940020
Anti-human CD127	BD Bioscience	940012
Anti-human CD41a	BD Bioscience	940219
Anti-human CD110	BD Bioscience	940302
Anti-human CD49f	BD Bioscience	940160
Anti-human CD90	BD Bioscience	940032
Anti-human CD34	BD Bioscience	940021
**Oligonucleotides and other sequence-based reagents**		
NA		
**Chemicals, enzymes and other reagents**		
IMDM	PAN biotech	P04-20150
Stemspan SFEM	Stemcell Technologies	09650
GlutaMax	Thermo Fisher Scientific	35050061
PenStrep	Thermo Fisher Scientific	15140122
Pyruvate	Thermo Fisher Scientific	11360070
PBS	Thermo Fisher Scientific	10010-015
Fetal bovine serum	Sigma-Aldrich	F7524-500ml
EDTA	Invitrogen	15575-038
HBSS without Ca2 + /Mg2+	Corning	21-022-CV
HBSS with Ca2+/Mg2+	Thermo Fisher Scientific	14025-050
Recombinant human SCF	Thermo Fisher Scientific	300-07
Recombinant human FLT3L	Thermo Fisher Scientific	300-19
Recombinant human TPO	Thermo Fisher Scientific	300-18
Recombinant human IL-1α	Thermo Fisher Scientific	17830783
UM171	Selleckchem	discontinued
DMSO	Sigma-Aldrich	D2438
Parmodulin-2	MCE	HY-13965
Human thrombin	Sigma-Aldrich	T6884
Human native activated Protein C	Invitrogen	RP-43095
Human FcR blocking reagent	Miltenyi Biotech	130-059-901
Normal mouse serum	Invitrogen	50-112-1171
Normal rat serum	Invitrogen	10-710-C
PDL-coated 96-well plate	Agilent	103798-100
Seahorse XF DMEM	Agilent	103575-100
Glucose solution	Agilent	103577-100
Mito stress test	Agilent	103015-100
2-Deoxyglucose	Sigma-Aldrich	D6134-1G
7-AAD viability staining solution	BioLegend	420404
Propidium iodide (PI)	MP Bio	02195458-CF
FoxP3/transcription factor staining kit	eBioscience	00-5523-00
Paraformaldehyde 16%	Thermo Fisher Scientific	28908
Methanol	Carl Roth	0082.2
Fluo-4-AM	MCE	MCE-HY-101896-100uG
Rhod-2-AM	MCE	MCE-HY-D0989-50uG
Verapamil hydrochlorid	Chemodex	CDX-V0007-M100
Thapsigargin	Cyaman Chemical	CAY-10522-1
DAPI solution	Miltenyi Biotec	130-111-570
CFSE Cell Division Tracker Kit	BioLegend	423801
BrdU	Thermo Fisher Scientific	H27260.03
RNase-free DNase Set	Qiagen	79254
Human Methylcellulose Complete Media	R&D systems	HSC003
TMRM	MCE	MCE-HY-D0984
APC Calibrite beads	BD Biosciences	340487
BD Rhapsody cDNA Kit	BD Biosciences	633773
BD Rhapsody Cartridge Kit	BD Biosciences	633733
BD Hu Single Cell Sample Multiplexing Kit	BD Biosciences	633781
BD Rhapsody Enhanced Cartridge Reagent Kit	BD Biosciences	664887
BD Rhapsody WTA Amplification Kit	BD Biosciences	633801
Bioanalyzer High Sensitivity DNA Kit	Agilent	5067-4626
AMPure XP Beads	Beckman Coulter	A63881
EDTA-coated 20 µL microcapillaries	Sarstedt	19.447.001
EDTA-coated tubes	Sarstedt	20.1278.100
**Software**		
FlowJo v10	BD Bioscience	
Prism 10.6.10	GraphPad	
CytExpert v2.7	Beckman Coulter	
Venny 2.1	https://bioinfogp.cnb.csic.es/tools/venny/ Oliveros, 2007–2015	
ShinyGO 0.85.1	https://bioinformatics.sdstate.edu/go/ Ge et al, 2020	
**Other**		
Seahorse XFe96	Agilent	
2100 Bioanalyzer	Agilent	
Aurora 5 L	Cytek	
FACSAria III 4 L	BD Bioscience	
Miltenyi MACSQuant 10	Miltenyi Biotec	
Cytoflex S	Beckman Coulter	
CytoFlex LX	Beckman Coulter	
Illumina Novaseq X Plus	Illumina	

### Animals

All mice were housed in an AAALAC-accredited animal facility at the University of California, Santa Cruz (UCSC) and maintained and used under approved Institutional Animal Care and Use Committee (IACUC) guidelines under the protocol FORSC-2312. Barrier mouse rooms were temperature-controlled and on a 12-h day/night cycle. Mice were kept in specific pathogen-free conditions in individually ventilated cages. Mice had ad libitum access to water and food, and daily care assessments. Strains used for this study were male and female NOD.Cg-*Kit*^*W-41J*^ *Tyr*^*+*^ *Prkdc*^*scid*^ *Il2rg*^*tm1Wjl*^/ThomJ (NBSGW, Jackson Laboratory #026622, in-house breeding UCSC) at the age of 4-6 weeks.

### Cells and cell culture

All CD34^+^ cells were bought from commercial providers and sourced from healthy adult donors after informed consent was given. Unmobilized BM aspirate-derived CD34^+^ cells were bought from BioIVT and mobilized peripheral blood (mPB) CD34^+^ cells from BioIVT, Lonza or Cyto-Care.eu GmbH either as purified CD34^+^ cells or as apheresis bags and enriched in-house.

Cryopreserved CD34^+^ cells were thawed, washed with IMDM (PAN biotech, #P04-20150) and resuspended in Stemspan SFEM (Stemcell technologies, #09650), 1% GlutaMax (Thermo Fisher Scientific, #35050061), 1% Penstep (Thermo Fisher Scientific #15140122), 100 ng/mL recombinant human SCF (Thermo Fisher Scientific # 300-07), 100 ng/mL recombinant human FLT3L (Thermo Fisher Scientific #300-19), 50 ng/mL recombinant human TPO (Thermo Fisher Scientific #300-18) and 35 nM UM171 (Selleckchem, discontinued) and plated in non-coated 24-well plates. Half medium changes were performed every day, and cells were replated if necessary. Cells were cultivated at 37 °C in 5% CO_2_, 21% oxygen and 90% humidity.

### Flow cytometry of CD34+ cells

CD34^+^ cells were stained in FACS buffer (PBS, EDTA 5 mM, 2% FCS) after incubation with FcR blocking reagent (Miltenyi). Cells were incubated with antibodies (see supplemental Flow Cytometry panel 1) for 30 min at 4 °C in the dark and then washed with FACS buffer. Dead cells were excluded with 7-AAD. Flow cytometry was performed on an Aurora 5 L flow cytometer (Cytek Bioscience), and data were analysed using FlowJo 10.10 (BD Biosciences).

### Xenograft transplantation

Two days after cultivation, CD34^+^ cells were washed, counted (hemocytometer), and an equal number of cells were incubated in a total volume of 1 mL full culture media supplemented either 10 µM parmodulin 2 (MCE #HY-13965) in 0.1% DMSO (Sigma-Aldrich #D2438) or 0.1% DMSO for 30 min at 37 °C (water bath). After 30 min of incubation, cells were stimulated with either 1 U/mL human thrombin (Sigma-Aldrich #T6884) or 10 µg/mL aPC (Invitrogen #RP-43095) for 60 min. Cells were washed twice with 9 ml HBSS without calcium, magnesium (Corning #21-022-CV) and resuspended in HBSS. For primary transplantation, 2.5 × 10^5^ CD34+ cells were injected into the retrobulbar venous plexus of unconditioned female and male NOD.Cg-*Kit*^*W-41J*^ *Tyr* ^+^ *Prkdc*^*scid*^ *Il2rg*^*tm1Wjl*^/ThomJ (NBSGW) mice under isoflurane anesthesia. For secondary transplantation, 2.5 × 10^6^ whole BM cells of human engrafted primary recipient mice were transplanted into non-conditioned secondary NBSGW mice, as described above.

Humanization of primary and secondary transplanted NBSGW recipient mice was assessed 4 weeks post-transplantation via flow cytometry. The humanization (=human cell chimerism) of NBSGW recipient mice was calculated as (Count human cells/(Count human CD45-positive cells + count mouse CD45-positive cells)). All transplanted mice were included in the analysis, and cells from all primary transplanted mice were transplanted into a secondary recipient.

### Peripheral blood sampling

For blood sampling, the mice were short-term anesthetized with isoflurane (3–4%). Whole peripheral blood was obtained via retrobulbar venous plexus puncture using EDTA-coated 20 µL microcapillaries (Sarstedt #19.447.001), collecting 1–2 drops into EDTA-coated tubes (Sarstedt #20.1278.100). About 5 µL blood was mixed with FACS buffer antibodies and a defined number of APC Calibrite beads (BD Biosciences #340487) at room temperature (RT), specific for flow cytometric analysis of peripheral blood for platelets and erythrocytes (see supplemental Flow Cytometry panel 3) or for nucleated blood cells (see supplemental Flow Cytometry panel 4).

Whole blood samples were stained for 20 min at RT and diluted with 295 µL of staining media. For nucleated cells, 15 µL of whole blood was stained for 20 min at RT, followed by RBC lysis at RT in 1 mL of RBC lysis buffer (0.15 M NH₄Cl, 0.01 M Tris-HCl, pH 7.5, 5 min). Cells were washed and acquired at the CytoFLEX LX (Beckman Coulter).

### Bone marrow cell sampling

Recipient mice were euthanized by CO_2_ inhalation and cervical dislocation. For quantification of absolute cell numbers, a known concentration of APC Calibrite beads (BD Biosciences, Cat# 340487) was added to each sample prior to processing.

Whole bone marrow cells were isolated from femur, tibiae, and hips by spinning (2500×*g*, 1 min, RT) into 500 µL FACS buffer. Red blood cells (RBCs) were lysed by a 5-min incubation at RT in 1 mL of RBC lysis buffer (0.15 M NH₄Cl, 0.01 M Tris-HCl, pH 7.5) and samples washed (300×*g*, 5 min, 4 °C) and resuspended in 500 µL of FACS buffer supplemented with 1:100 human FcR blocking reagent (Miltenyi Biotech #130-059-901) and 5% normal mouse (Invitrogen #50-112-1171) and 5% normal rat (Invitrogen #10-710-C) serum. 20% of the volume was used for flow cytometric staining (see supplemental Flow Cytometry panel 5) on ice for 30 min. Cells were all acquired at the CytoFLEX LX (Beckman Coulter).

### Single-cell expansion assay

Stimulated CD34^+^ cells were FcR blocked (Miltenyi) and stained with antibodies (see supplemental Flow Cytometry Panel 2), and single CD34^+^ cells were sorted with the ARIA III (BD Biosciences) into 100 µL complete medium (without stimuli) in a 96-well U-bottom plate (Greiner Bio-one). Single cells were cultivated for 7 days. All wells were stained (supplemental Flow Cytometry Panel 2), and samples were acquired using the CytoFLEX LX (Beckman Coulter).

### Extracellular flux

1 × 10^5^ CD34^+^ cells were plated (PDL-coated 96-well plate, Agilent, #103798-100) per well in Seahorse XF DMEM (Agilent, #103575-100), with 1 mM pyruvate (Thermo Fisher Scientific, #11360070), 1% GlutaMax, and 10 mM glucose (Agilent, #103577-100) containing DMSO (0.1%) ±  Parmodulin-2 (10 uM). Mito Stress test (Agilent, #103015-100) was performed by injecting aPC or THR after baseline recording, followed by Oligomycin (1.5 mM), FCCP (0.5 mM) and Rotenone/Antimycin A (0.5 mM) according to manufacturer’s instructions. Glycostress test was performed by plating 100k cells per well in DMEM without glucose with DMSO (0.1%) ± Parmodulin-2 (10 µM). Cells were starved for 1 h at 37 °C in a non-CO_2_ incubator. To measure glycolysis, aPC or THR was coinjected with glucose (10 mM), followed by oligomycin (1.5 mM) and 2-deoxyglucose (50 mM, Sigma, #D6134-1G) injection according to the manufacturer’s instructions. The acute response was calculated by subtracting the last value of the baseline from the first value after stimulation.

### Single-cell RNAseq

CD34^+^ cells were cultivated for 48 h before stimulation with aPC or THR for 4 and 24 h. Cells were washed and incubated for 10 min with Fc blocking reagent before staining with oligo-tagged antibodies (see Appendix scRNAseq Panel) according to the manufacturer’s instructions for 30 min on ice. Cells were washed and each sample was separately incubated with one sample tag from the human multiplexing kit for 30 min before two washes. Equal number of cells from each sample were pooled and loaded onto one BD Rhapsody cartridge, cell lysis, cDNA and exonuclease treatment, WTA, sample tag and antibody library preparation were down according to manufacturer’s guide. Quality and concentration of each library was tested with an Agilent bioanalyzer, and libraries were pooled in a ratio of 1:1:28 (sample tag:ABseq:WTA). Sequencing was performed at the Functional Genomic Center Zurich on an Illumina Novaseq X Plus using 4 lanes of a 10B flow cell with 1 billion reads per lane.

### Data analysis of scRNAseq

Multiplets, dead cells and sample tag negative cells were excluded, and analysis was conducted in ScSeurat. Per sample tag, 6965 to 9024 cells were included for analysis. Cells were clustered using weighted nearest neighbor (WNN) analysis by integrating WTA and BD®AbSeq data. Reference-based annotation was conducted by comparing our WTA data to published data sets (Sauvageau, 2020a; Sauvageau, 2020b) consisting of CD34^+^ cells, which were expanded for 7 days in the same medium we are using (Lehnertz et al, 2021). About 17,909 (96.6%) genes were overlapping from our data set and the reference data sets and labels transferred to our data set, creating an annotated data set with an average median prediction score of 0.72. Cutoff for differentially expressed genes (DEGs) were a log2 fold change of 0.25 and an adjusted *p* value <0.05. Gene ontology of DEGs was analysed using ShinyGO 0.85 (Ge et al, 2020), with a false discovery rate (FDR) cutoff of 0.05. Cell cycle phase was annotated according to (Tirosh et al, 2016).

### PAR1 internalization and aPC/EPCR binding

CD34^+^ cells were cultivated for 2 days before stimulation with aPC or THR. aPC-binding to EPCR was blocked by preincubation of cells for 1 h at 37 °C using anti-EPCR-PE-Vio 770 (Miltenyi, #130-122-978, 1:10). After the indicated timepoints, cells were washed with FACS buffer. Cell surface PAR1 expression was assessed by staining cells after incubation of FcR blocking agent for 5 min with CD34-Vioblue (1:100) and PAR1-PE (Clone Span12 to detect only intact PAR1, Beckman Coulter #IM2583, clone WEDE15, Beckman Coulter #IM2584, to detect activated and not activated PAR1, both 1:50). Staining was performed for 30 min at 4 °C in the dark. To stain internalized PAR1, stimulated cells were washed, fixed, and permeabilized using the FoxP3/transcription factor staining kit (eBioscience: #00-5523-00). Fixation/permeabilization was performed for 30 min at room temperature. Cells were washed with Permeabilization buffer, after which cells were stained in permeabilization buffer with CD34-Vioblue and PAR1-PE (clone WEDE15) at room temperature for 30 min. Cells were washed and acquired on the MACSquant Analyzer 10 (Miltenyi) or Cytoflex S (Beckman Coulter).

### Phosphoflow

CD34^+^ cells were expanded for 7 days. In some cases, CD34^+^EPCR^−^ cells were sorted beforehand using an FACSAria III 4 L (BD Bioscience). On the day of the assay, cells were washed with IMDM and resuspended in Stemspan SFEM without any supplements or cytokines, but with or without PM2 (10 µM) or an EPCR-blocking antibody (EPCR-PE-Vio 770, Miltenyi, #130-122-978, 1:10), divided into a 96-well plate and starved for 1 h at 37 °C. aPC or THR was added for the indicated time and concentration, and then cells were directly fixed by the addition of 250 µl 4% PFA in PBS for 15 min at room temperature. Cells were washed twice with FACS buffer, and after the second wash, resuspended in pure methanol at −20 °C. Samples were stored at −80 °C for multiple days, before dividing each sample into three to four subsamples which were washed with FACS buffer twice and resuspended in FcR block and stained with CD34-Vioblue, PAR1-PE (WEDE15) and one phospho-specific antibody, anti-pAKT-AF647 (1:10, BD Bioscience #560343), anti-pERK1/2-AF647 (1:10, BD Bioscience, #612593), anti-pNfkB-AF647 (1:10, BD Bioscience # 558422) or anti-pCREB-AF647 (1:200, Cell Signaling technology #14001). Cells were stained for 1 h at room temperature before washing twice.

### Ca^2+^ flux

Expanded CD34^+^ cells were counted and stained with the following antibodies: CD34-PE-Cy7(1:100, Biolegend #343516), CD45RA-BV786 (1:100, BD Bioscience, #563870) and CD90-AF700 (1:100, Biolegend, #328120) and EPCR-APC (1:50, Biolegend, #351906) 30 min on ice in dark, washed and resuspended at 10^7^cells/ml in Ca/Mg-free PBS with either 1 µM Fluo-4-AM (MCE, #MCE-HY-101896-100uG) or 2.5 µM Rhod-2-AM (MCE, #MCE-HY-D0989-50uG) and 50 µM Verapamil hydrochlorid (Chemodex, #CDX-V0007-M100) and incubated for 30 min at room temperature in the dark, then washed and resuspended in PBS with 50 µM Verapamil. During flow cytometry recording, THR, aPC or Thapsigargin (Cayman Chemical # CAY-10522-1) were added after 20 s of baseline recording as a 10x solution and mixed into the sample by pipetting. Parmodulin 2 was added 5 min before recording. DAPI (1:100, Miltenyi # 130-111-570) was used to exclude dead cells.

### CFSE dye dilution

Thawed human CD34^+^ cells were stained with CFSE (1 µM, BioLegend # 423801) for 5 min at 37 °C in 1 ml PBS. The staining was stopped by the addition of 10 ml IMDM with 10% FBS and incubation for a further 15 min at 37 °C. Cells were washed, resuspended in medium and cultivated for 72 h. aPC was added 4 h prior to IL-1α (Peprotech # 17830783) addition. A small portion of cells was taken after 24 h to determine the starting value for CFSE labeling.

### BrdU incorporation and cell cycle analysis

CD34^+^ cells were cultivated after thawing in full medium containing 5 µM BrdU (Thermo Fisher Scientific, # H27260.03). A 50% medium change was performed once after 24 h with medium containing BrdU. After 48 h, cells were washed and cell surface staining performed with CD34-Vioblue (1:100, Miltenyi #130-113-182) on ice for 30 min, followed by washing. In some cases, due to small cell numbers, the unstained murine fibroblast cell line SC-1 was added to bulk up cells. The Foxp3/Transcription Factor kit (eBioscience # 00-5521-00) was used to fix and permeabilize the cells for 30 min at room temperature. After that, cells were washed and resuspended in DNase solution (30 units/sample in HBSS) and incubated for 1 h at 37 °C. Cells were then washed and stained in permeabilization buffer with the following antibodies: Ki67-PE-Cy7 (1:100, eBioscience # 25-5698-82) and BrdU-FITC (1:24, eBioscience, #11-5071-42) antibody for 1 h at room temperature, before a final wash and staining of DNA with 7-AAD (1:10). Samples were acquired on a Cytek Aurora and SC-1 cells were excluded from flow cytometry analysis by lack of CD34 staining and cell size.

### TET2 and 5-hmC staining

CD34^+^ cells were expanded for 7 days with indicated stimuli, before washing, FcR blocking and staining for CD34-Vioblue (1:100, Miltenyi # 130-113-182), CD45RA-BV786 (1:100, BD Bioscience, #563870), and CD90-AF700 (1:100, Biolegend, #328120). Cells were washed after 30 min and fixation/permeabilization was done using the FoxP3/transcription factor staining kit (eBioscience: #00-5523-00 for 30 min at room temperature. TET2 was stained using the polyclonal TET2-CoraLite 488 antibody (Proteintech, # CL488-21207-100UL, 1:50) for 1 h at room temperature. In case 5-hmC was stained, fixed cells were resuspended in DNase solution (30 units/sample in HBSS) and incubated for 1 h at 37 °C, before washing with permeabilization buffer. 5-hmC was stained using anti-5-hmC antibody (Active Motif, #39092, 10 µg/ml final concentration) for 1 h at room temperature before secondary staining for 1 h using goat anti-rabbit-antibody conjugated to Alexa Fluor 647 (Invitrogen, #A21244, 1:500).

### Colony-forming unit assay

1000 human CD34^+^ cells were added to methylcellulose-containing medium (R&D systems #HSC003) and 1 ml plated in duplicates. Colonies were evaluated microscopically after 12 days, and for secondary plating, cells were detached from the initial plate by washing with warm PBS with 5 mM EDTA, counted and 10,000 live cells were replated in a new methylcellulose-containing medium and scored after 13 days.

### Measurement of methylglyoxal proteins adducts

CD34^+^ cells were cultivated in complete medium for 2 days before stimulation with either aPC (10 µg/ml), THR (1 U/ml) or PBS in 50 ul medium in a 96-well plate for 30 min at 37 °C. Without washing, 200 ul pure methanol (−20 °C) was added to each well. Cells were stored at −20 °C before washing with PBS. Methylglyoxal protein adducts were stained using a monoclonal Methylglyoxal-FITC antibody for 30 min at 4 °C (Clone: 9E7, Sigma-Aldrich # SAB5202574-100UG), diluted 1:50 in FACS buffer. Cells were washed with FACS buffer and acquired using an Aurora 5 L flow cytometer (Cytek).

### TMRM staining

CD34^+^ cells were cultivated in complete medium for 2 days. Cells were washed with warm PBS and stained with TMRM (20 nM, MCE, #MCE-HY-D0984) in PBS with Verapamil hydrochloride (50 µM) for 30 min at 37 °C in the dark. Cells were then washed and resuspended in fresh complete medium, supplemented with verapamil. Cells were stimulated as indicated and after the indicated timepoints washed with ice cold FACS buffer with verapamil (50 µM) and stained with the following antibodies: CD34-Vioblue (1:100, Miltenyi #130-113-182), CD45RA-BV786 (1:100, BD Bioscience, #563870) and CD90-AF700 (1:100, BioLegend, #328120) for 30 min on ice, before final washing and acquisition on an Aurora 5L (Cytek). TMRM was also used to exclude dead cells, since they do not possess a mitochondrial membrane potential.

### Statistical analysis

Statistical tests (GraphPad Prism 10.6.10) are described in the figure legends. In case only selected multiple comparisons were analysed, all selected comparisons are indicated, including non-significant findings. Venn diagrams were created using Venny 2.1 (Oliveros, 2007). Sample size was not estimated before conducting experiments, and personal was not blinded. NBSGW mice were allocated randomly to different groups; otherwise no randomization was performed. Unless a technical failure occurred, all samples were included in the analysis. Normal distribution was assumed for all in vitro experiments. For in experiments involving NBSGW mice, a non-normal distribution was assumed, as reported previously (McIntosh et al, 2015), unless otherwise indicated.

## Supplementary information

Appendix

Peer Review File

Source data Fig. 1

Source data Fig. 2

Source data Fig. 4

Source data Fig. 5

Source data Fig. 6

Source data Fig. 7

Expanded View Figures

## Data Availability

scRNAseq data set is available under https://www.ncbi.nlm.nih.gov/geo/query/acc.cgi?acc=GSE313745. The source data of this paper are collected in the following database record: biostudies:S-SCDT-10_1038-S44321-026-00529-y.

## References

[CR1] Aibibula M, Naseem KM, Sturmey RG (2018) Glucose metabolism and metabolic flexibility in blood platelets. J Thromb Haemost 16:2300–2314 10.1111/jth.14274 30151891

[CR2] Aisiku O, Peters CG, de Ceunynck K, Ghosh CC, Dilks JR, Fustolo-Gunnink SF, Huang M, Dockendorff C, Parikh SM, Flaumenhaft R (2015) Parmodulins inhibit thrombus formation without inducing endothelial injury caused by vorapaxar. Blood 125:1976–1985 10.1182/blood-2014-09-599910 PMC4366627 25587041

[CR3] Allaman I, Bélanger M, Magistretti PJ (2015) Methylglyoxal, the dark side of glycolysis. Front Neurosci 9:23 10.3389/fnins.2015.00023 PMC4321437 25709564

[CR4] Anjos-Afonso F, Buettner F, Mian SA, Rhys H, Perez-Lloret J, Garcia-Albornoz M, Rastogi N, Ariza-McNaughton L, Bonnet D (2022) Single cell analyses identify a highly regenerative and homogenous human CD34+ hematopoietic stem cell population. Nat Commun 13:2048 10.1038/s41467-022-29675-w PMC9018830 35440586

[CR5] Balazs AB, Fabian AJ, Esmon CT, Mulligan RC (2006) Endothelial protein C receptor (CD201) explicitly identifies hematopoietic stem cells in murine bone marrow. Blood 107:2317–2321 10.1182/blood-2005-06-2249 PMC1895725 16304059

[CR6] Basu S, Liang HPH, Hernandez I, Zogg M, Fields B, May J, Ogoti Y, Wyseure T, Mosnier LO, Burns RT et al (2020) Role of thrombomodulin expression on hematopoietic stem cells. J Thromb Haemost 18:123–135 10.1111/jth.14663 PMC6940513 31628891

[CR7] Becker HJ, Ishida R, Wilkinson AC, Kimura T, Lee MSJ, Coban C, Ota Y, Tanaka Y, Roskamp M, Sano T et al (2023) Controlling genetic heterogeneity in gene-edited hematopoietic stem cells by single-cell expansion. Cell Stem Cell 30:987–1000.e8 10.1016/j.stem.2023.06.002 PMC10338855 37385251

[CR8] Bhatia M, Wang JC, Kapp U, Bonnet D, Dick JE (1997) Purification of primitive human hematopoietic cells capable of repopulating immune-deficient mice. Proc Natl Acad Sci USA 94:5320–5325 10.1073/pnas.94.10.5320 PMC24676 9144235

[CR9] Bisht K, Barbier V, Shatunova S, Winkler IG, Lévesque J-P (2026) Endothelial protein C receptor CD201 is a better marker than stem cell antigen-1 to identify mouse long-term reconstituting hematopoietic stem cells following septic challenge. Exp Hematol 154:105326 10.1016/j.exphem.2025.105326 41270997

[CR10] Brass LF, Pizarro S, Ahuja M, Belmonte E, Blanchard N, Stadel JM, Hoxie JA (1994) Changes in the structure and function of the human thrombin receptor during receptor activation, internalization, and recycling. J Biol Chem 269:2943–2952 7507931

[CR11] Carey A, Edwards 5thDK, Eide CA, Newell L, Traer E, Medeiros BC, Pollyea DA, Deininger MW, Collins RH, Tyner JW et al (2017) Identification of interleukin-1 by functional screening as a key mediator of cellular expansion and disease progression in acute myeloid leukemia. Cell Rep 18:3204–3218 10.1016/j.celrep.2017.03.018 PMC5437102 28355571

[CR12] Chavez JS, Rabe JL, Hernandez G, Mills TS, Niño KE, Davizon-Castillo P, Pietras EM (2022) PU.1 expression defines distinct functional activities in the phenotypic HSC compartment of a murine inflammatory stress model. Cells 11:680 10.3390/cells11040680 PMC8870714 35203330

[CR13] Che JLC, Bode D, Kucinski I, Cull AH, Bain F, Becker HJ, Jassinskaja M, Barile M, Boyd G, Belmonte M et al (2022) Identification and characterization of in vitro expanded hematopoietic stem cells. EMBO Rep 23:e55502 10.15252/embr.202255502 PMC9535767 35971894

[CR14] Choo S, Wolf CB, Mack HM, Egan MJ, Kiem H-P, Radtke S (2024) Choosing the right mouse model: comparison of humanized NSG and NBSGW mice for in vivo HSC gene therapy. Blood Adv 8:916–926 10.1182/bloodadvances.2023011371 PMC10877116 38113461

[CR15] Collins TJ, Lipp P, Berridge MJ, Bootman MD (2001) Mitochondrial Ca(2+) uptake depends on the spatial and temporal profile of cytosolic Ca(2+) signals. J Biol Chem 276:26411–26420 10.1074/jbc.M101101200 11333261

[CR16] de Ceunynck K, Peters CG, Jain A, Higgins SJ, Aisiku O, Fitch-Tewfik JL, Chaudhry SA, Dockendorff C, Parikh SM, Ingber DE et al (2018) PAR1 agonists stimulate APC-like endothelial cytoprotection and confer resistance to thromboinflammatory injury. Proc Natl Acad Sci USA 115:E982–E991 10.1073/pnas.1718600115 PMC5798377 29343648

[CR18] Dempfle C-EH, Elmas E, Link A, Suvajac N, Liebe V, Janes J, Borggrefe M (2011) Endogenous plasma activated protein C levels and the effect of enoxaparin and drotrecogin alfa (activated) on markers of coagulation activation and fibrinolysis in pulmonary embolism. Crit Care 15:R23 10.1186/cc9968 PMC3222057 21241489

[CR19] Dorrell C, Gan OI, Pereira DS, Hawley RG, Dick JE (2000) Expansion of human cord blood CD34+CD38−cells in ex vivo culture during retroviral transduction without a corresponding increase in SCID repopulating cell (SRC) frequency: dissociation of SRC phenotype and function. Blood 95:102–110 10607692

[CR20] Fares I, Chagraoui J, Lehnertz B, MacRae T, Mayotte N, Tomellini E, Aubert L, Roux PP, Sauvageau G (2017) EPCR expression marks UM171-expanded CD34+ cord blood stem cells. Blood 129:3344–3351 10.1182/blood-2016-11-750729 28408459

[CR21] Farlik M, Halbritter F, Müller F, Choudry FA, Ebert P, Klughammer J, Farrow S, Santoro A, Ciaurro V, Mathur A et al (2016) DNA methylation dynamics of human hematopoietic stem cell differentiation. Cell Stem Cell 19:808–822 10.1016/j.stem.2016.10.019 PMC5145815 27867036

[CR22] Fukudome K, Esmon CT (1994) Identification, cloning, and regulation of a novel endothelial cell protein C/activated protein C receptor. J Biol Chem 269:26486–26491 7929370

[CR23] Gandhi DM, Majewski MW, Rosas R, Kentala K, Foster TJ, Greve E, Dockendorff C (2018) Characterization of protease-activated receptor (PAR) ligands: parmodulins are reversible allosteric inhibitors of PAR1-driven calcium mobilization in endothelial cells. Bioorg Med Chem 26:2514–2529 10.1016/j.bmc.2018.04.016 PMC5937995 29685684

[CR24] Ge SX, Jung D, Yao R (2020) ShinyGO: a graphical gene-set enrichment tool for animals and plants. Bioinformatics 36:2628–2629 10.1093/bioinformatics/btz931 PMC7178415 31882993

[CR25] Glimm H, Oh IH, Eaves CJ (2000) Human hematopoietic stem cells stimulated to proliferate in vitro lose engraftment potential during their S/G(2)/M transit and do not reenter G(0). Blood 96:4185–4193 11110690

[CR26] Grimsey NJ, Coronel LJ, Cordova IC, Trejo J (2016) Recycling and endosomal sorting of protease-activated receptor-1 Is Distinctly Regulated by Rab11A and Rab11B proteins. J Biol Chem 291:2223–2236 10.1074/jbc.M115.702993 PMC4732206 26635365

[CR28] Gupta D, Elwakiel A, Ranjan S, Pandey MK, Krishnan S, Ambreen S, Henschler R, Rana R, Keller M, Ceglarek U et al (2023) Activated protein C modulates T-cell metabolism and epigenetic FOXP3 induction via α-ketoglutarate. Blood Adv 7:5055–5068 10.1182/bloodadvances.2023010083 PMC10471940 37315174

[CR29] Gur-Cohen S, Itkin T, Chakrabarty S, Graf C, Kollet O, Ludin A, Golan K, Kalinkovich A, Ledergor G, Wong E et al (2015) PAR1 signaling regulates the retention and recruitment of EPCR-expressing bone marrow hematopoietic stem cells. Nat Med 21:1307–1317 10.1038/nm.3960 PMC4776769 26457757

[CR30] Hajnóczky G, Robb-Gaspers LD, Seitz MB, Thomas AP (1995) Decoding of cytosolic calcium oscillations in the mitochondria. Cell 82:415–424 10.1016/0092-8674(95)90430-1 7634331

[CR31] Hammond CA, Wu SW, Wang F, MacAldaz ME, Eaves CJ (2023) Aging alters the cell cycle control and mitogenic signaling responses of human hematopoietic stem cells. Blood 141:1990–2002 10.1182/blood.2022017174 36652668

[CR32] Henschler R, Piiper A, Bistrian R, Möbest D (2003) SDF-1alpha-induced intracellular calcium transient involves Rho GTPase signalling and is required for migration of hematopoietic progenitor cells. Biochem Biophys Res Commun 311:1067–1071 10.1016/j.bbrc.2003.10.112 14623290

[CR33] Hess NJ, Lindner PN, Vazquez J, Grindel S, Hudson AW, Stanic AK, Ikeda A, Hematti P, Gumperz JE (2020) Different human immune lineage compositions are generated in non-conditioned NBSGW mice depending on HSPC source. Front Immunol 11:573406 10.3389/fimmu.2020.573406 PMC7604455 33193358

[CR34] Hogart A, Lichtenberg J, Ajay SS, Anderson S, Margulies EH, Bodine DM (2012) Genome-wide DNA methylation profiles in hematopoietic stem and progenitor cells reveal overrepresentation of ETS transcription factor binding sites. Genome Res 22:1407–1418 10.1101/gr.132878.111 PMC3409254 22684279

[CR36] Iwasaki H, Arai F, Kubota Y, Dahl M, Suda T (2010) Endothelial protein C receptor-expressing hematopoietic stem cells reside in the perisinusoidal niche in fetal liver. Blood 116:544–553 10.1182/blood-2009-08-240903 20442369

[CR37] Jetmore A, Plett PA, Tong X, Wolber FM, Breese R, Abonour R, Orschell-Traycoff CM, Srour EF (2002) Homing efficiency, cell cycle kinetics, and survival of quiescent and cycling human CD34(+) cells transplanted into conditioned NOD/SCID recipients. Blood 99:1585–1593 10.1182/blood.v99.5.1585 11861272

[CR38] Johnson CS, Williams M, Sham K, Belluschi S, Ma W, Wang X, Lau WWY, Kaufmann KB, Krivdova G, Calderbank EF et al (2024) Adaptation to ex vivo culture reduces human hematopoietic stem cell activity independently of the cell cycle. Blood 144:729–741 10.1182/blood.2023021426 PMC7616366 38805639

[CR39] Kent DG, Copley MR, Benz C, Wöhrer S, Dykstra BJ, Ma E, Cheyne J, Zhao Y, Bowie MB, Zhao Y et al (2009) Prospective isolation and molecular characterization of hematopoietic stem cells with durable self-renewal potential. Blood 113:6342–6350 10.1182/blood-2008-12-192054 19377048

[CR40] Kohlscheen S, Schenk F, Rommel MGE, Cullmann K, Modlich U (2019) Endothelial protein C receptor supports hematopoietic stem cell engraftment and expansion in Mpl-deficient mice. Blood 133:1465–1478 10.1182/blood-2018-03-837344 30683655

[CR41] Kovtonyuk LV, Caiado F, Garcia-Martin S, Manz E-M, Helbling P, Takizawa H, Boettcher S, Al-Shahrour F, Nombela-Arrieta C, Slack E et al (2022) IL-1 mediates microbiome-induced inflammaging of hematopoietic stem cells in mice. Blood 139:44–58 10.1182/blood.2021011570 34525198

[CR42] Künze G, Isermann B (2023) Targeting biased signaling by PAR1: function and molecular mechanism of parmodulins. Blood 141:2675–2684 10.1182/blood.2023019775 PMC10646804 36952648

[CR44] Lehnertz B, Chagraoui J, MacRae T, Tomellini E, Corneau S, Mayotte N, Boivin I, Durand A, Gracias D, Sauvageau G (2021) HLF expression defines the human hematopoietic stem cell state. Blood 138:2642–2654 10.1182/blood.2021010745 34499717

[CR45] Li L, Xu D, Huang X (2025) SERCA-mediated endoplasmic reticulum stress facilitates hematopoietic stem cell mobilization. Stem Cell Res Ther 16:208 10.1186/s13287-025-04345-y PMC12023629 40275396

[CR46] Lin DS, Trumpp A (2023) Differential expression of endothelial protein C receptor (EPCR) in hematopoietic stem and multipotent progenitor cells in young and old mice. Cells Dev 174:203843 10.1016/j.cdev.2023.203843 37080459

[CR47] Luchsinger LL, Strikoudis A, Danzl NM, Bush EC, Finlayson MO, Satwani P, Sykes M, Yazawa M, Snoeck H-W (2019) Harnessing hematopoietic stem cell low intracellular calcium improves their maintenance in vitro. Cell Stem Cell 25:225–240.e7 10.1016/j.stem.2019.05.002 PMC7649753 31178255

[CR48] Mac NA, Prasad S, Capece GE, Martínez-Zamudio RI, McClenaghan C, Poole LG (2025) Identification of novel gene expression programs in thrombin-stimulated hepatic stellate cells. Res Pract Thromb Haemost 9:103223 10.1016/j.rpth.2025.103223 PMC12637384 41278464

[CR49] Majeti R, Park CY, Weissman IL (2007) Identification of a hierarchy of multipotent hematopoietic progenitors in human cord blood. Cell Stem Cell 1:635–645 10.1016/j.stem.2007.10.001 PMC2292126 18371405

[CR51] McIntosh BE, Brown ME, Duffin BM, Maufort JP, Vereide DT, Slukvin II, Thomson JA (2015) Nonirradiated NOD,B6.SCID Il2rγ-/- Kit(W41/W41) (NBSGW) mice support multilineage engraftment of human hematopoietic cells. Stem Cell Rep 4:171–180 10.1016/j.stemcr.2014.12.005 PMC4325197 25601207

[CR52] McKenzie JL, Gan OI, Doedens M, Dick JE (2007) Reversible cell surface expression of CD38 on CD34-positive human hematopoietic repopulating cells. Exp Hematol 35:1429–1436 10.1016/j.exphem.2007.05.017 17656009

[CR54] Molinar-Inglis O, Birch CA, Nicholas D, Orduña-Castillo L, Cisneros-Aguirre M, Patwardhan A, Chen B, Grimsey NJ, Coronel LJ, Lin H et al (2021) aPC/PAR1 confers endothelial anti-apoptotic activity via a discrete, β-arrestin-2-mediated SphK1-S1PR1-Akt signaling axis. Proc Natl Acad Sci USA 118:e2106623118 10.1073/pnas.2106623118 PMC8670512 34873055

[CR55] Molino M, Bainton DF, Hoxie JA, Coughlin SR, Brass LF (1997) Thrombin receptors on human platelets. Initial localization and subsequent redistribution during platelet activation. J Biol Chem 272:6011–6017 10.1074/jbc.272.9.6011 9038223

[CR56] Morganti C, Cabezas-Wallscheid N, Ito K (2022) Metabolic regulation of hematopoietic stem cells. Hemasphere 6:e740 10.1097/HS9.0000000000000740 PMC9242402 35785147

[CR57] Munz CM, Dressel N, Chen M, Grinenko T, Roers A, Gerbaulet A (2023) Regeneration after blood loss and acute inflammation proceeds without contribution of primitive HSCs. Blood 141:2483–2492 10.1182/blood.2022018996 36787502

[CR58] Notta F, Doulatov S, Laurenti E, Poeppl A, Jurisica I, Dick JE (2011) Isolation of single human hematopoietic stem cells capable of long-term multilineage engraftment. Science 333:218–221 10.1126/science.1201219 21737740

[CR59] Notta F, Zandi S, Takayama N, Dobson S, Gan OI, Wilson G, Kaufmann KB, McLeod J, Laurenti E, Dunant CF et al (2016) Distinct routes of lineage development reshape the human blood hierarchy across ontogeny. Science 351:aab2116 10.1126/science.aab2116 PMC4816201 26541609

[CR60] Oliveros JC (2007) Venny: an interactive tool for comparing lists with Venn’s diagrams. https://bioinfogp.cnb.csic.es/tools/venny/index.html

[CR61] Pagel CN, Song S-J, Loh LH, Tudor EM, Murray-Rust TA, Pike RN, Mackie EJ (2009) Thrombin-stimulated growth factor and cytokine expression in osteoblasts is mediated by protease-activated receptor-1 and prostanoids. Bone 44:813–821 10.1016/j.bone.2008.12.031 19442625

[CR62] Park CY, Majeti R, Weissman IL (2008) In vivo evaluation of human hematopoiesis through xenotransplantation of purified hematopoietic stem cells from umbilical cord blood. Nat Protoc 3:1932–1940 10.1038/nprot.2008.194 19180077

[CR63] Pepler L, Yu P, Dwivedi DJ, Trigatti BL, Liaw PC (2015) Characterization of mice harboring a variant of EPCR with impaired ability to bind protein C: novel role of EPCR in hematopoiesis. Blood 126:673–682 10.1182/blood-2014-02-558940 26045607

[CR64] Pietras EM, Mirantes-Barbeito C, Fong S, Loeffler D, Kovtonyuk LV, Zhang S, Lakshminarasimhan R, Chin CP, Techner J-M, Will B et al (2016) Chronic interleukin-1 exposure drives haematopoietic stem cells towards precocious myeloid differentiation at the expense of self-renewal. Nat Cell Biol 18:607–618 10.1038/ncb3346 PMC4884136 27111842

[CR65] Rabe JL, Hernandez G, Chavez JS, Mills TS, Nerlov C, Pietras EM (2020) CD34 and EPCR coordinately enrich functional murine hematopoietic stem cells under normal and inflammatory conditions. Exp Hematol 81:1–15.e6 10.1016/j.exphem.2019.12.003 PMC6938677 31863798

[CR66] Rahman A, Anwar KN, Uddin S, Xu N, Ye RD, Platanias LC, Malik AB (2001) Protein kinase C-delta regulates thrombin-induced ICAM-1 gene expression in endothelial cells via activation of p38 mitogen-activated protein kinase. Mol Cell Biol 21:5554–5565 10.1128/MCB.21.16.5554-5565.2001 PMC87277 11463837

[CR67] Rahmig S, Kronstein-Wiedemann R, Fohgrub J, Kronstein N, Nevmerzhitskaya A, Bornhäuser M, Gassmann M, Platz A, Ordemann R, Tonn T et al (2016) Improved human erythropoiesis and platelet formation in humanized NSGW41 mice. Stem Cell Rep 7:591–601 10.1016/j.stemcr.2016.08.005 PMC5063583 27618723

[CR68] Riewald M, Petrovan RJ, Donner A, Mueller BM, Ruf W (2002) Activation of endothelial cell protease activated receptor 1 by the protein C pathway. Science 296:1880–1882 10.1126/science.1071699 12052963

[CR69] Robb-Gaspers LD, Burnett P, Rutter GA, Denton RM, Rizzuto R, Thomas AP (1998) Integrating cytosolic calcium signals into mitochondrial metabolic responses. EMBO J 17:4987–5000 10.1093/emboj/17.17.4987 PMC1170827 9724635

[CR70] Rommel MGE, Walz L, Fotopoulou F, Kohlscheen S, Schenk F, Miskey C, Botezatu L, Krebs Y, Voelker IM, Wittwer K et al (2022) Influenza A virus infection instructs hematopoiesis to megakaryocyte-lineage output. Cell Rep 41:111447 10.1016/j.celrep.2022.111447 36198277

[CR71] Sakurai M, Ishitsuka K, Ito R, Wilkinson AC, Kimura T, Mizutani E, Nishikii H, Sudo K, Becker HJ, Takemoto H et al (2023) Chemically defined cytokine-free expansion of human haematopoietic stem cells. Nature 615:127–133 10.1038/s41586-023-05739-9 36813966

[CR72] Sauvageau G (2020a) Gene expression omnibus GSM4643123. https://www.ncbi.nlm.nih.gov/geo/query/acc.cgi?acc=GSM4643123

[CR73] Sauvageau G (2020b) Gene expression omnibus GSM4643124. https://www.ncbi.nlm.nih.gov/geo/query/acc.cgi?acc=GSM4643124

[CR74] Shin JJ, Schröder MS, Caiado F, Wyman SK, Bray NL, Bordi M, Dewitt MA, Vu JT, Kim W-T, Hockemeyer D et al (2020) Controlled cycling and quiescence enables efficient HDR in engraftment-enriched adult hematopoietic stem and progenitor cells. Cell Rep 32:108093 10.1016/j.celrep.2020.108093 PMC7487781 32877675

[CR76] Song SJ, Pagel CN, Campbell TM, Pike RN, Mackie EJ (2005) The role of protease-activated receptor-1 in bone healing. Am J Pathol 166:857–868 10.1016/S0002-9440(10)62306-1 PMC1602347 15743797

[CR77] Spencer JA, Ferraro F, Roussakis E, Klein A, Wu J, Runnels JM, Zaher W, Mortensen LJ, Alt C, Turcotte R et al (2014) Direct measurement of local oxygen concentration in the bone marrow of live animals. Nature 508:269–273 10.1038/nature13034 PMC3984353 24590072

[CR78] Steidl U, Kronenwett R, Rohr U-P, Fenk R, Kliszewski S, Maercker C, Neubert P, Aivado M, Koch J, Modlich O et al (2002) Gene expression profiling identifies significant differences between the molecular phenotypes of bone marrow-derived and circulating human CD34+ hematopoietic stem cells. Blood 99:2037–2044 10.1182/blood.v99.6.2037 11877277

[CR79] Subramaniam A, Talkhoncheh MS, Magnusson M, Larsson J (2019) Endothelial protein C receptor (EPCR) expression marks human fetal liver hematopoietic stem cells. Haematologica 104:e47–e50 10.3324/haematol.2018.198515 PMC6355479 30026339

[CR80] Subramaniam A, Žemaitis K, Talkhoncheh MS, Yudovich D, Bäckström A, Debnath S, Chen J, Jain MV, Galeev R, Gaetani M et al (2020) Lysine-specific demethylase 1A restricts ex vivo propagation of human HSCs and is a target of UM171. Blood 136:2151–2161 10.1182/blood.2020005827 PMC7645986 32582923

[CR81] Thastrup O, Cullen PJ, Drøbak BK, Hanley MR, Dawson AP (1990) Thapsigargin, a tumor promoter, discharges intracellular Ca2+ stores by specific inhibition of the endoplasmic reticulum Ca2(+)-ATPase. Proc Natl Acad Sci USA 87:2466–2470 10.1073/pnas.87.7.2466 PMC53710 2138778

[CR82] Tirosh I, Izar B, Prakadan SM, Wadsworth MH, Treacy D, Trombetta JJ, Rotem A, Rodman C, Lian C, Murphy G et al (2016) Dissecting the multicellular ecosystem of metastatic melanoma by single-cell RNA-seq. Science 352:189–196 10.1126/science.aad0501 PMC4944528 27124452

[CR83] Tsutsumi N, Masoumi Z, James SC, Tucker JA, Winkelmann H, Grey W, Picton LK, Moss L, Wilson SC, Caveney NA et al (2023) Structure of the thrombopoietin-MPL receptor complex is a blueprint for biasing hematopoiesis. Cell 186:4189–4203.e22 10.1016/j.cell.2023.07.037 PMC10528194 37633268

[CR84] Ugarte F, Forsberg EC (2013) Haematopoietic stem cell niches: new insights inspire new questions. EMBO J 32:2535–2547 10.1038/emboj.2013.201 PMC3791375 24022369

[CR85] Umemoto T, Hashimoto M, Matsumura T, Nakamura-Ishizu A, Suda T (2018) Ca2+-mitochondria axis drives cell division in hematopoietic stem cells. J Exp Med 215:2097–2113 10.1084/jem.20180421 PMC6080917 29946000

[CR86] Vilas-Boas EA, Cabral-Costa JV, Ramos VM, Da Caldeira Silva CC, Kowaltowski AJ (2023) Goldilocks calcium concentrations and the regulation of oxidative phosphorylation: too much, too little, or just right. J Biol Chem 299:102904 10.1016/j.jbc.2023.102904 PMC9947387 36642177

[CR87] Wang W, Zhang Y, Dettinger P, Reimann A, Kull T, Loeffler D, Manz MG, Lengerke C, Schroeder T (2021) Cytokine combinations for human blood stem cell expansion induce cell-type- and cytokine-specific signaling dynamics. Blood 138:847–857 10.1182/blood.2020008386 33988686

[CR88] Wiekmeijer A-S, Pike-Overzet K, Brugman MH, Salvatori DCF, Egeler RM, Bredius RGM, Fibbe WE, Staal FJT (2014) Sustained engraftment of cryopreserved human bone marrow CD34(+) cells in young adult NSG mice. Biores Open Access 3:110–116 10.1089/biores.2014.0008 PMC4048975 24940562

[CR89] Williams DA, Kohn DB, Thrasher AJ (2025) Ex vivo modification of hematopoietic stem and progenitor cells for gene therapy. Mol Ther 33:2141–2153 10.1016/j.ymthe.2025.03.058 PMC12126793 40176348

[CR90] Xu C, Lv X, Yang S, Lv Y, Zheng Y, Wang Y-X, Hui Y, Sun G, Zhao X, Ma L-Y et al (2026) Procr+ endothelial progenitor cells govern hematopoiesis through fine-tuning mesenchymal stem cell niche signals. Blood 148:841–855 10.1182/blood.2025031474 42101967

[CR91] Yamazaki S, Iwama A, Takayanagi S, Morita Y, Eto K, Ema H, Nakauchi H (2006) Cytokine signals modulated via lipid rafts mimic niche signals and induce hibernation in hematopoietic stem cells. EMBO J 25:3515–3523 10.1038/sj.emboj.7601236 PMC1538571 16858398

[CR93] Zheng X, Li W, Song Y, Hu Y, Ferrell GL, Esmon NL, Esmon CT (2007) Non-hematopoietic EPCR regulates the coagulation and inflammatory responses during endotoxemia. J Thromb Haemost 5:1394–1400 10.1111/j.1538-7836.2007.02592.x 17445091

