# Supplementary material for: Activated protein C promotes hematopoietic stem and progenitor cell quiescence and engraftment
Source: EMBO Mol Med. 2026 Oct 9;18(11):4433–67. doi: 10.1038/s44321-026-00529-y (PMC13748566; doi:10.1038/s44321-026-00529-y)
Supplement: Supplementary file 1 [file 44321_2026_529_MOESM1_ESM.pdf]

Appendix

Table of contents:

Appendix Figure S1: Gating scheme: human CD34+ subsets………….……………………...2
Appendix Figure S2: Gating scheme: human cells in NBSGW mice………………………..3
Appendix Table S1……………………………………………...………………………....4
Appendix Table S2 ……….……..……………………………………………….………...5
Appendix Table S3………………...……………………………………………………….6
Appendix Table S4……………………………………………………………………..….8
Appendix Table S5……………………………………………………………………….37
Appendix Table S6……………………………………………………………………….39
Appendix Table S7……………………………………………………………………….41
Appendix Table S8……………………………………………………………………….44
Appendix Table S9……………………………………………………………………….46
Appendix Table S10………...……………………………………………………………48
Appendix Table S11……...………………………………………………………………49
Appendix Table S12………………..…………………………………………………….51
Appendix Table S13……………………..……………………………………………….53
Appendix Table S14……………………..……………………………………………….55
Flow Cytometry Panels …………………………………………………………………57

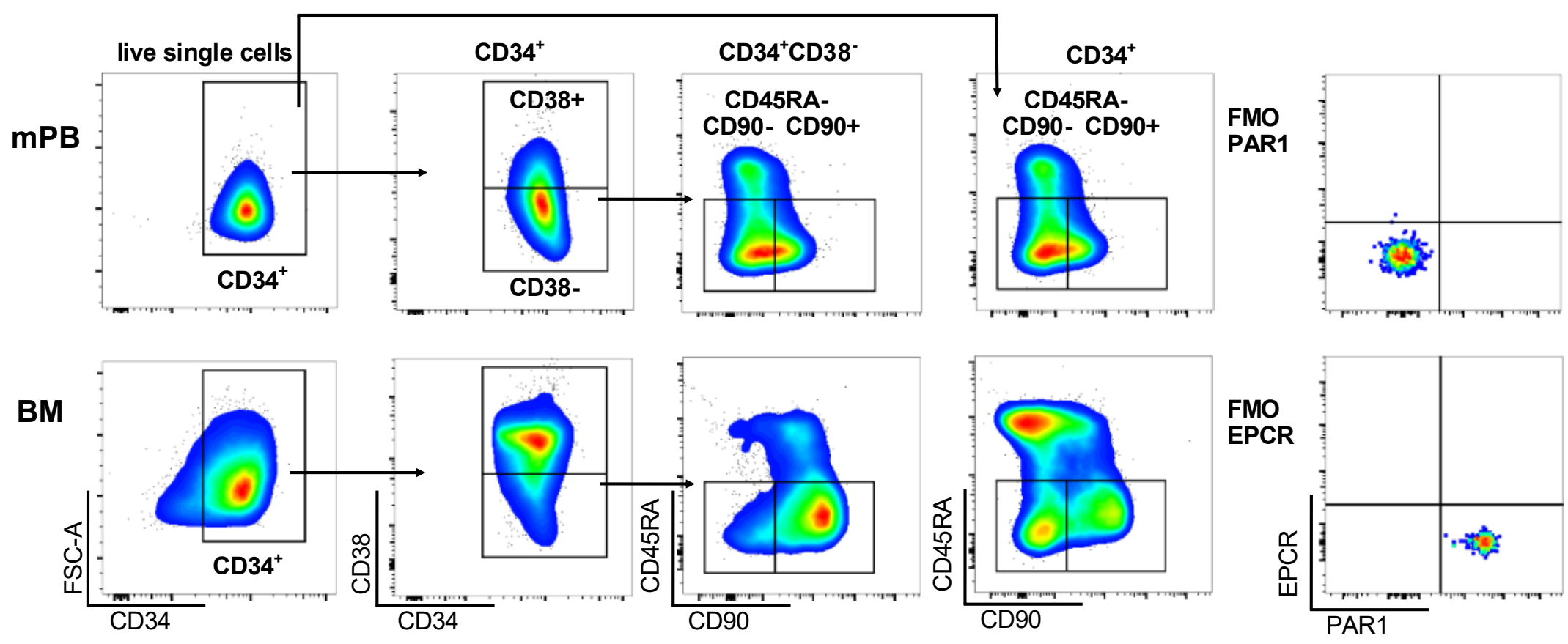

Appendix Figure S1: **Gating schemes for human CD34+ subsets**

Representative flow cytometry (FC) gating scheme for analysis of BM and mPB samples.

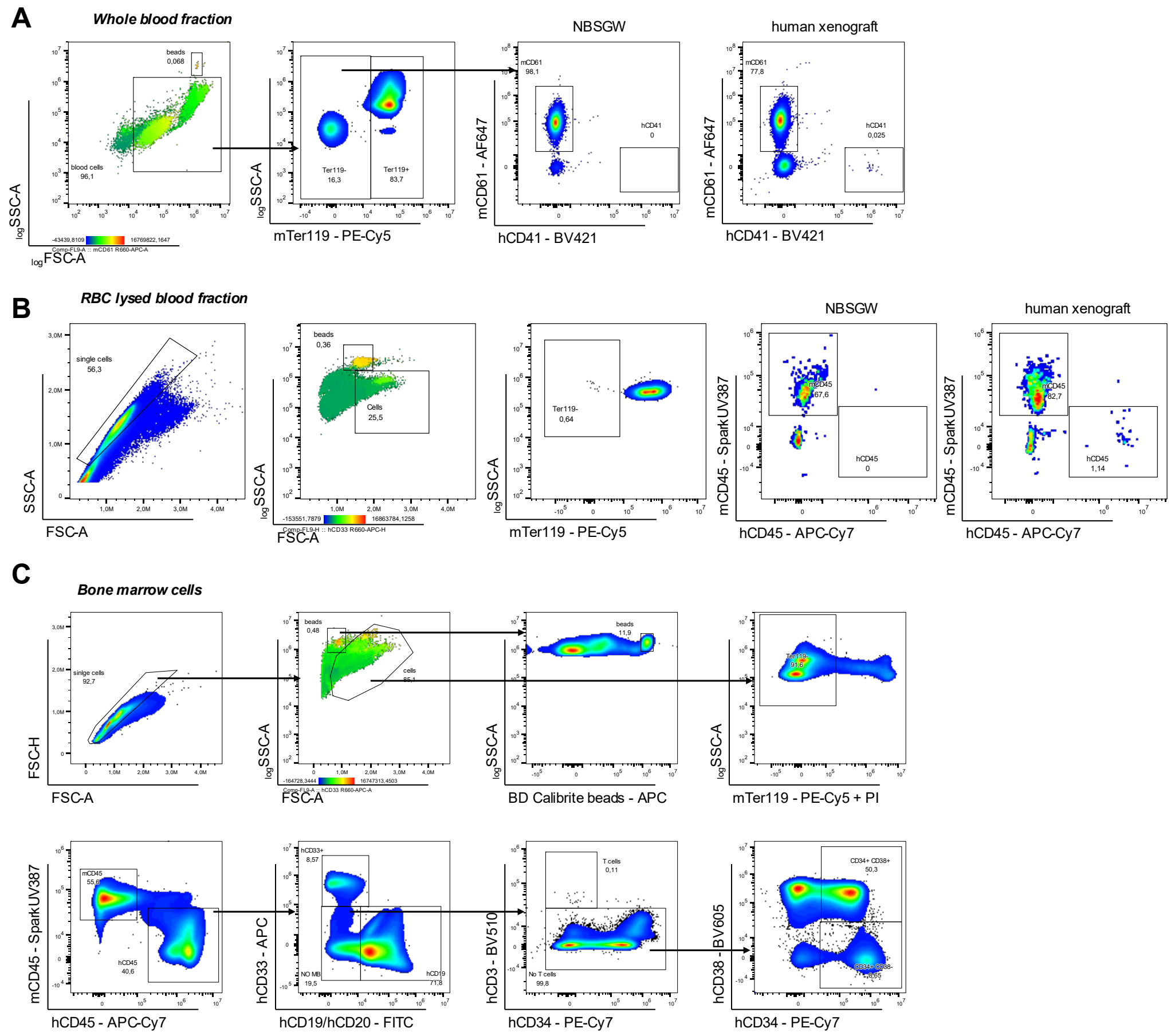

Appendix Figure S2: Gating scheme: human cells in NBSGW mice

Representative gating schemes for analysis of **(A)** human and murine platelets in the whole blood fraction, **(B)** human leukocytes in RBC lysed blood fraction **(C)** human cells in the bone marrow of transplanted NBSGW mice.

Appendix Table S1 to Figure EV1C and 1C: results from two-way Anova with Holm-Šídák's multiple comparison test, comparing EPCR expression of CD34+CD45RA-CD90+ with CD34+CD45RA-CD90- cells for each day.

BM %EPCR+

		CD34+CD45RA-CD90+ - CD34+CD45RA-CD90-			
		Mean diff.	Below threshold?	Summary	Adjusted P Value
days in culture	0	-0.2343	No	ns	0.9645
	2	21	Yes	**	0.0019
	5	28.79	Yes	***	0.0001
	7	40.98	Yes	****	<0.0001

mPB %EPCR+

		CD34+CD45RA-CD90+ - CD34+CD45RA-CD90-			
		Predicted (LS) Mean diff.	Below threshold?	Summary	Adjusted P Value
days in culture	0	0.4504	No	ns	0.9291
	2	24.71	Yes	****	<0.0001
	5	25.31	Yes	****	<0.0001
	7	31.24	Yes	****	<0.0001

Appendix Table S2 to Figures 2 G-J**:** results from two-way Anova with Holm-Šídák's multiple comparison test, comparing fold change of phosphorylated CREB, AKT, ERK1/2 and NFκB between different stimuli for each time point.

phospho-CREB

	Comparison	Mean diff.	Below threshold?	Summary	Adjusted P Value
10min	THR vs. aPC	-3.968	Yes	****	<0.0001
	THR vs. PM2 + THR	3.176	Yes	****	<0.0001
	aPC vs. PM2 + THR	7.145	Yes	****	<0.0001
30min	THR vs. aPC	1.621	Yes	*	0.0179
	THR vs. PM2 + THR	1.579	Yes	*	0.0179
	aPC vs. PM2 + THR	-0.04196	No	ns	0.9408
60min	THR vs. aPC	-0.5657	No	ns	0.6849
	THR vs. PM2 + THR	-0.2114	No	ns	0.7807
	aPC vs. PM2 + THR	0.3542	No	ns	0.7807
120min	THR vs. aPC	0.3374	No	ns	0.7986
	THR vs. PM2 + THR	0.4715	No	ns	0.7903
	aPC vs. PM2 + THR	0.1341	No	ns	0.8124

phospho-AKT

	Comparison	Mean diff.	Below threshold?	Summary	Adjusted P Value
10min	THR vs. aPC	-0.01353	No	ns	0.9195
	THR vs. PM2 + THR	0.2242	No	ns	0.2242
	aPC vs. PM2 + THR	0.2377	No	ns	0.2242
30min	THR vs. aPC	-0.4062	Yes	**	0.0076
	THR vs. PM2 + THR	0.2778	Yes	*	0.0427
	aPC vs. PM2 + THR	0.6841	Yes	****	<0.0001
60min	THR vs. aPC	-0.449	Yes	**	0.0046
	THR vs. PM2 + THR	-0.06574	No	ns	0.6241
	aPC vs. PM2 + THR	0.3832	Yes	*	0.0122
120min	THR vs. aPC	0.02586	No	ns	0.9661
	THR vs. PM2 + THR	-0.03122	No	ns	0.9661
	aPC vs. PM2 + THR	-0.05707	No	ns	0.9642

phospho-ERK1/2

	Comparison	Mean diff.	Below threshold?	Summary	Adjusted P Value
10min	THR vs. aPC	0.09314	No	ns	0.523
	THR vs. PM2 + THR	0.1299	No	ns	0.4038
	aPC vs. PM2 + THR	0.0368	No	ns	0.6865
30min	THR vs. aPC	0.285	Yes	**	0.0059
	THR vs. PM2 + THR	0.329	Yes	**	0.0022
	aPC vs. PM2 + THR	0.04396	No	ns	0.6298
60min	THR vs. aPC	0.5397	Yes	****	<0.0001
	THR vs. PM2 + THR	0.5495	Yes	****	<0.0001
	aPC vs. PM2 + THR	0.009756	No	ns	0.9147
120min	THR vs. aPC	0.4045	Yes	***	0.0001
	THR vs. PM2 + THR	0.4323	Yes	****	<0.0001
	aPC vs. PM2 + THR	0.02789	No	ns	0.7596

phospho-NFκB

	Comparison	Mean diff.	Below threshold?	Summary	Adjusted P Value
10min	THR vs. aPC	0.003628	No	ns	0.9526
	THR vs. PM2 + THR	0.09597	No	ns	0.321
	aPC vs. PM2 + THR	0.09234	No	ns	0.321
30min	THR vs. aPC	0.007572	No	ns	0.9013
	THR vs. PM2 + THR	0.09357	No	ns	0.3423
	aPC vs. PM2 + THR	0.086	No	ns	0.3423
60min	THR vs. aPC	0.01829	No	ns	0.7646
	THR vs. PM2 + THR	0.06798	No	ns	0.6091
	aPC vs. PM2 + THR	0.04969	No	ns	0.6606
120min	THR vs. aPC	-0.005408	No	ns	0.9294
	THR vs. PM2 + THR	0.03716	No	ns	0.8891
	aPC vs. PM2 + THR	0.04257	No	ns	0.8891

Appendix Table S3: P values reported in Figure 1

Fig. 1B	
Holm-Šídák's multiple comparisons test	Adjusted P Value
CD34+	
0 vs. 2	0.9362
0 vs. 5	0.0029
0 vs. 7	0.0335
CD34+CD45RA-CD90+	
0 vs. 2	0.9839
0 vs. 5	0.9839
0 vs. 7	0.9839
CD34+CD45RA-CD90-	
0 vs. 2	0.9435
0 vs. 5	<0.0001
0 vs. 7	<0.0001

Fig. 1C	
Holm-Šídák's multiple comparisons test	Adjusted P Value
CD34+	
0 vs. 2	0.0117
0 vs. 5	0.1229
0 vs. 7	0.0117
CD34+CD45RA-CD90+	
0 vs. 2	<0.0001
0 vs. 5	<0.0001
0 vs. 7	<0.0001
CD34+CD45RA-CD90-	
0 vs. 2	0.1151
0 vs. 5	0.5754
0 vs. 7	0.5244

Fig. 1D	
Tukey's multiple comparisons test	Adjusted P Value
$CD34^-$ vs. $CD34^+$	0.0002
$CD34^-$ vs. $CD90^-$	0.0013
$CD34^-$ vs. $CD90^+$	<0.0001
$CD34^-$ vs. $EPCR^-$	<0.0001
$CD34^-$ vs. $EPCR^+$	<0.0001
$CD34^+$ vs. $CD90^-$	0.9721
$CD34^+$ vs. $CD90^+$	0.0748
$CD34^+$ vs. $EPCR^-$	0.4394
$CD34^+$ vs. $EPCR^+$	0.0002
$CD90^-$ vs. $CD90^+$	0.0125
$CD90^-$ vs. $EPCR^-$	0.1211
$CD90^-$ vs. $EPCR^+$	<0.0001
$CD90^+$ vs. $EPCR^-$	0.9175
$CD90^+$ vs. $EPCR^+$	0.2268
$EPCR^-$ vs. $EPCR^+$	0.0282

Fig 1E	
Tukey's multiple comparisons test	Adjusted P Value
unstim vs. aPC 10 µg/ml	<0.0001
unstim vs. THR 1U/ml	<0.0001
aPC 10 µg/ml vs. THR 1U/ml	0.4033

Fig. 1F	
Holm-Šídák's multiple comparisons test	Adjusted P Value
unstim vs. THR	0.0001
unstim vs. 2h	0.0007
unstim vs. 4h	0.0025
unstim vs. 24h	0.6575
unstim 24h vs. 24h	0.1098
unstim vs. unstim 24h	0.0669

Fig. 1G	
Holm-Šídák's multiple comparisons test	Adjusted P Value
unstim vs. 2h	0.0037
unstim vs. 4h	0.0108
unstim vs. 24h	0.1276
unstim 24h vs. 24h	0.0935
unstim vs. unstim 24h	0.0074

Fig. 1H	
Unpaired t test	P Value
SPAN 12 vs. WEDE15	0.6246

Appendix Table S4: P values reported in Figure 2

Fig. 2A kinetics<br>Holm-Šídák's multiple comparisons test	Adjusted P Value
sec 1	
unstimulated vs. THR 1U/ml	0.7603
unstimulated vs. aPC 10ug/ml	0.7063
sec 2	
unstimulated vs. THR 1U/ml	0.6979
unstimulated vs. aPC 10ug/ml	0.6068
sec 3	
unstimulated vs. THR 1U/ml	0.8262
unstimulated vs. aPC 10ug/ml	0.8262
sec 4	
unstimulated vs. THR 1U/ml	0.5903
unstimulated vs. aPC 10ug/ml	0.786
sec 5	
unstimulated vs. THR 1U/ml	0.8598
unstimulated vs. aPC 10ug/ml	0.8598
sec 6	
unstimulated vs. THR 1U/ml	0.8583
unstimulated vs. aPC 10ug/ml	0.8787
sec 7	
unstimulated vs. THR 1U/ml	0.7958
unstimulated vs. aPC 10ug/ml	0.8227
sec 8	
unstimulated vs. THR 1U/ml	0.8005
unstimulated vs. aPC 10ug/ml	0.8531
sec 9	
unstimulated vs. THR 1U/ml	0.9198
unstimulated vs. aPC 10ug/ml	0.9198
sec 10	
unstimulated vs. THR 1U/ml	0.8258
unstimulated vs. aPC 10ug/ml	0.8258
sec 11	
unstimulated vs. THR 1U/ml	0.7931
unstimulated vs. aPC 10ug/ml	0.9576
sec 12	
unstimulated vs. THR 1U/ml	0.6811
unstimulated vs. aPC 10ug/ml	0.9893
sec 13	
unstimulated vs. THR 1U/ml	0.5208
unstimulated vs. aPC 10ug/ml	0.9469
sec 14	
unstimulated vs. THR 1U/ml	0.6515
unstimulated vs. aPC 10ug/ml	0.8032

sec 15	
unstimulated vs. THR 1U/ml	0.6201
unstimulated vs. aPC 10ug/ml	0.9462
sec 16	
unstimulated vs. THR 1U/ml	0.7758
unstimulated vs. aPC 10ug/ml	0.7758
sec 17	
unstimulated vs. THR 1U/ml	0.7287
unstimulated vs. aPC 10ug/ml	0.9424
sec 18	
unstimulated vs. THR 1U/ml	0.6232
unstimulated vs. aPC 10ug/ml	0.7097
sec 19	
unstimulated vs. THR 1U/ml	0.8164
unstimulated vs. aPC 10ug/ml	0.8164
sec 20	
unstimulated vs. THR 1U/ml	0.7587
unstimulated vs. aPC 10ug/ml	0.9629
sec 21	
unstimulated vs. THR 1U/ml	0.7559
unstimulated vs. aPC 10ug/ml	0.7828
sec 22	
unstimulated vs. THR 1U/ml	0.7296
unstimulated vs. aPC 10ug/ml	0.8338
sec 23	
unstimulated vs. THR 1U/ml	0.6261
unstimulated vs. aPC 10ug/ml	0.7205
sec 24	
unstimulated vs. THR 1U/ml	0.73
unstimulated vs. aPC 10ug/ml	0.826
sec 25	
unstimulated vs. THR 1U/ml	0.5621
unstimulated vs. aPC 10ug/ml	0.7764
sec 26	
unstimulated vs. THR 1U/ml	0.4558
unstimulated vs. aPC 10ug/ml	0.9771
sec 27	
unstimulated vs. THR 1U/ml	0.357
unstimulated vs. aPC 10ug/ml	0.8356
sec 28	
unstimulated vs. THR 1U/ml	0.7486
unstimulated vs. aPC 10ug/ml	0.8326
sec 29	
unstimulated vs. THR 1U/ml	0.6572
unstimulated vs. aPC 10ug/ml	0.8937

sec 30	
unstimulated vs. THR 1U/ml	0.5541
unstimulated vs. aPC 10ug/ml	0.987
sec 31	
unstimulated vs. THR 1U/ml	0.3368
unstimulated vs. aPC 10ug/ml	0.9188
sec 32	
unstimulated vs. THR 1U/ml	0.5322
unstimulated vs. aPC 10ug/ml	0.9903
sec 33	
unstimulated vs. THR 1U/ml	0.6456
unstimulated vs. aPC 10ug/ml	0.6854
sec 34	
unstimulated vs. THR 1U/ml	0.5992
unstimulated vs. aPC 10ug/ml	0.9035
sec 35	
unstimulated vs. THR 1U/ml	0.6179
unstimulated vs. aPC 10ug/ml	0.7094
sec 36	
unstimulated vs. THR 1U/ml	0.2926
unstimulated vs. aPC 10ug/ml	0.9913
sec 37	
unstimulated vs. THR 1U/ml	0.1691
unstimulated vs. aPC 10ug/ml	0.9352
sec 38	
unstimulated vs. THR 1U/ml	0.0208
unstimulated vs. aPC 10ug/ml	0.717
sec 39	
unstimulated vs. THR 1U/ml	0.0021
unstimulated vs. aPC 10ug/ml	0.8445
sec 40	
unstimulated vs. THR 1U/ml	0.0012
unstimulated vs. aPC 10ug/ml	0.8818
sec 41	
unstimulated vs. THR 1U/ml	<0.0001
unstimulated vs. aPC 10ug/ml	0.8957
sec 42	
unstimulated vs. THR 1U/ml	<0.0001
unstimulated vs. aPC 10ug/ml	0.9021
sec 43	
unstimulated vs. THR 1U/ml	<0.0001
unstimulated vs. aPC 10ug/ml	0.8333
sec 44	
unstimulated vs. THR 1U/ml	<0.0001
unstimulated vs. aPC 10ug/ml	0.9227

sec 45	
unstimulated vs. THR 1U/ml	<0.0001
unstimulated vs. aPC 10ug/ml	0.8595
sec 46	
unstimulated vs. THR 1U/ml	<0.0001
unstimulated vs. aPC 10ug/ml	0.8845
sec 47	
unstimulated vs. THR 1U/ml	<0.0001
unstimulated vs. aPC 10ug/ml	0.9327
sec 48	
unstimulated vs. THR 1U/ml	<0.0001
unstimulated vs. aPC 10ug/ml	0.8111
sec 49	
unstimulated vs. THR 1U/ml	<0.0001
unstimulated vs. aPC 10ug/ml	0.8015
sec 50	
unstimulated vs. THR 1U/ml	<0.0001
unstimulated vs. aPC 10ug/ml	0.7618
sec 51	
unstimulated vs. THR 1U/ml	<0.0001
unstimulated vs. aPC 10ug/ml	0.9775
sec 52	
unstimulated vs. THR 1U/ml	<0.0001
unstimulated vs. aPC 10ug/ml	0.9009
sec 53	
unstimulated vs. THR 1U/ml	<0.0001
unstimulated vs. aPC 10ug/ml	0.9029
sec 54	
unstimulated vs. THR 1U/ml	<0.0001
unstimulated vs. aPC 10ug/ml	0.9668
sec 55	
unstimulated vs. THR 1U/ml	<0.0001
unstimulated vs. aPC 10ug/ml	0.9118
sec 56	
unstimulated vs. THR 1U/ml	<0.0001
unstimulated vs. aPC 10ug/ml	0.9794
sec 57	
unstimulated vs. THR 1U/ml	<0.0001
unstimulated vs. aPC 10ug/ml	0.6108
sec 58	
unstimulated vs. THR 1U/ml	<0.0001
unstimulated vs. aPC 10ug/ml	0.79
sec 59	
unstimulated vs. THR 1U/ml	<0.0001
unstimulated vs. aPC 10ug/ml	0.9003

sec 60	
unstimulated vs. THR 1U/ml	<0.0001
unstimulated vs. aPC 10ug/ml	0.8611
sec 61	
unstimulated vs. THR 1U/ml	<0.0001
unstimulated vs. aPC 10ug/ml	0.8703
sec 62	
unstimulated vs. THR 1U/ml	<0.0001
unstimulated vs. aPC 10ug/ml	0.9159
sec 63	
unstimulated vs. THR 1U/ml	<0.0001
unstimulated vs. aPC 10ug/ml	0.934
sec 64	
unstimulated vs. THR 1U/ml	<0.0001
unstimulated vs. aPC 10ug/ml	0.9905
sec 65	
unstimulated vs. THR 1U/ml	<0.0001
unstimulated vs. aPC 10ug/ml	0.9715
sec 66	
unstimulated vs. THR 1U/ml	<0.0001
unstimulated vs. aPC 10ug/ml	0.9335
sec 67	
unstimulated vs. THR 1U/ml	<0.0001
unstimulated vs. aPC 10ug/ml	0.8265
sec 68	
unstimulated vs. THR 1U/ml	<0.0001
unstimulated vs. aPC 10ug/ml	0.8233
sec 69	
unstimulated vs. THR 1U/ml	<0.0001
unstimulated vs. aPC 10ug/ml	0.9577
sec 70	
unstimulated vs. THR 1U/ml	<0.0001
unstimulated vs. aPC 10ug/ml	0.8306
sec 71	
unstimulated vs. THR 1U/ml	<0.0001
unstimulated vs. aPC 10ug/ml	0.7639
sec 72	
unstimulated vs. THR 1U/ml	<0.0001
unstimulated vs. aPC 10ug/ml	0.742
sec 73	
unstimulated vs. THR 1U/ml	<0.0001
unstimulated vs. aPC 10ug/ml	0.9248
sec 74	
unstimulated vs. THR 1U/ml	<0.0001
unstimulated vs. aPC 10ug/ml	0.9433

sec 75	
unstimulated vs. THR 1U/ml	<0.0001
unstimulated vs. aPC 10ug/ml	0.9163
sec 76	
unstimulated vs. THR 1U/ml	<0.0001
unstimulated vs. aPC 10ug/ml	0.8983
sec 77	
unstimulated vs. THR 1U/ml	<0.0001
unstimulated vs. aPC 10ug/ml	0.85
sec 78	
unstimulated vs. THR 1U/ml	<0.0001
unstimulated vs. aPC 10ug/ml	0.9135
sec 79	
unstimulated vs. THR 1U/ml	0.0002
unstimulated vs. aPC 10ug/ml	0.9626
sec 80	
unstimulated vs. THR 1U/ml	<0.0001
unstimulated vs. aPC 10ug/ml	0.7896
sec 81	
unstimulated vs. THR 1U/ml	0.001
unstimulated vs. aPC 10ug/ml	0.9997
sec 82	
unstimulated vs. THR 1U/ml	0.0015
unstimulated vs. aPC 10ug/ml	0.998
sec 83	
unstimulated vs. THR 1U/ml	0.0015
unstimulated vs. aPC 10ug/ml	0.9915
sec 84	
unstimulated vs. THR 1U/ml	0.0022
unstimulated vs. aPC 10ug/ml	0.9929
sec 85	
unstimulated vs. THR 1U/ml	0.0022
unstimulated vs. aPC 10ug/ml	0.8832
sec 86	
unstimulated vs. THR 1U/ml	0.0103
unstimulated vs. aPC 10ug/ml	0.8117
sec 87	
unstimulated vs. THR 1U/ml	0.0224
unstimulated vs. aPC 10ug/ml	0.8127
sec 88	
unstimulated vs. THR 1U/ml	0.0314
unstimulated vs. aPC 10ug/ml	0.9477
sec 89	
unstimulated vs. THR 1U/ml	0.0357
unstimulated vs. aPC 10ug/ml	0.8839

sec 90	
unstimulated vs. THR 1U/ml	0.0147
unstimulated vs. aPC 10ug/ml	0.9327
sec 91	
unstimulated vs. THR 1U/ml	0.031
unstimulated vs. aPC 10ug/ml	0.9726
sec 92	
unstimulated vs. THR 1U/ml	0.0241
unstimulated vs. aPC 10ug/ml	0.9345
sec 93	
unstimulated vs. THR 1U/ml	0.0343
unstimulated vs. aPC 10ug/ml	0.9606
sec 94	
unstimulated vs. THR 1U/ml	0.0425
unstimulated vs. aPC 10ug/ml	0.9406
sec 95	
unstimulated vs. THR 1U/ml	0.037
unstimulated vs. aPC 10ug/ml	0.9868
sec 96	
unstimulated vs. THR 1U/ml	0.0832
unstimulated vs. aPC 10ug/ml	0.8495
sec 97	
unstimulated vs. THR 1U/ml	0.0992
unstimulated vs. aPC 10ug/ml	0.9572
sec 98	
unstimulated vs. THR 1U/ml	0.2159
unstimulated vs. aPC 10ug/ml	0.8195
sec 99	
unstimulated vs. THR 1U/ml	0.3554
unstimulated vs. aPC 10ug/ml	0.6859
sec 100	
unstimulated vs. THR 1U/ml	0.0817
unstimulated vs. aPC 10ug/ml	0.857
sec 101	
unstimulated vs. THR 1U/ml	0.2368
unstimulated vs. aPC 10ug/ml	0.8514
sec 102	
unstimulated vs. THR 1U/ml	0.4193
unstimulated vs. aPC 10ug/ml	0.7894
sec 103	
unstimulated vs. THR 1U/ml	0.3678
unstimulated vs. aPC 10ug/ml	0.9945
sec 104	
unstimulated vs. THR 1U/ml	0.3168
unstimulated vs. aPC 10ug/ml	0.9122

sec 105	
unstimulated vs. THR 1U/ml	0.3487
unstimulated vs. aPC 10ug/ml	0.9488
sec 106	
unstimulated vs. THR 1U/ml	0.3448
unstimulated vs. aPC 10ug/ml	0.8286
sec 107	
unstimulated vs. THR 1U/ml	0.3957
unstimulated vs. aPC 10ug/ml	0.984
sec 108	
unstimulated vs. THR 1U/ml	0.358
unstimulated vs. aPC 10ug/ml	0.9396
sec 109	
unstimulated vs. THR 1U/ml	0.6242
unstimulated vs. aPC 10ug/ml	0.8288
sec 110	
unstimulated vs. THR 1U/ml	0.5647
unstimulated vs. aPC 10ug/ml	0.8752
sec 111	
unstimulated vs. THR 1U/ml	0.4059
unstimulated vs. aPC 10ug/ml	0.8676
sec 112	
unstimulated vs. THR 1U/ml	0.379
unstimulated vs. aPC 10ug/ml	0.7875
sec 113	
unstimulated vs. THR 1U/ml	0.6082
unstimulated vs. aPC 10ug/ml	0.623
sec 114	
unstimulated vs. THR 1U/ml	0.3613
unstimulated vs. aPC 10ug/ml	0.9132
sec 115	
unstimulated vs. THR 1U/ml	0.5073
unstimulated vs. aPC 10ug/ml	0.9875
sec 116	
unstimulated vs. THR 1U/ml	0.4678
unstimulated vs. aPC 10ug/ml	0.961
sec 117	
unstimulated vs. THR 1U/ml	0.8102
unstimulated vs. aPC 10ug/ml	0.8102
sec 118	
unstimulated vs. THR 1U/ml	0.5076
unstimulated vs. aPC 10ug/ml	0.9272
sec 119	
unstimulated vs. THR 1U/ml	0.4773
unstimulated vs. aPC 10ug/ml	0.8164

sec 120	
unstimulated vs. THR 1U/ml	0.4868
unstimulated vs. aPC 10ug/ml	0.9953
sec 121	
unstimulated vs. THR 1U/ml	0.7889
unstimulated vs. aPC 10ug/ml	0.8074
sec 122	
unstimulated vs. THR 1U/ml	0.6892
unstimulated vs. aPC 10ug/ml	0.8385
sec 123	
unstimulated vs. THR 1U/ml	0.5911
unstimulated vs. aPC 10ug/ml	0.9393
sec 124	
unstimulated vs. THR 1U/ml	0.8573
unstimulated vs. aPC 10ug/ml	0.9107
sec 125	
unstimulated vs. THR 1U/ml	0.7597
unstimulated vs. aPC 10ug/ml	0.9903
sec 126	
unstimulated vs. THR 1U/ml	0.439
unstimulated vs. aPC 10ug/ml	0.9227
sec 127	
unstimulated vs. THR 1U/ml	0.7718
unstimulated vs. aPC 10ug/ml	0.9414
sec 128	
unstimulated vs. THR 1U/ml	0.7123
unstimulated vs. aPC 10ug/ml	0.9303
sec 129	
unstimulated vs. THR 1U/ml	0.6784
unstimulated vs. aPC 10ug/ml	0.903
sec 130	
unstimulated vs. THR 1U/ml	0.7723
unstimulated vs. aPC 10ug/ml	0.9568
sec 131	
unstimulated vs. THR 1U/ml	0.9172
unstimulated vs. aPC 10ug/ml	0.9172
sec 132	
unstimulated vs. THR 1U/ml	0.8782
unstimulated vs. aPC 10ug/ml	0.8782
sec 133	
unstimulated vs. THR 1U/ml	0.9925
unstimulated vs. aPC 10ug/ml	0.9925
sec 134	
unstimulated vs. THR 1U/ml	0.8967
unstimulated vs. aPC 10ug/ml	0.9034

sec 135	
unstimulated vs. THR 1U/ml	0.8509
unstimulated vs. aPC 10ug/ml	0.969
sec 136	
unstimulated vs. THR 1U/ml	0.7511
unstimulated vs. aPC 10ug/ml	0.8157
sec 137	
unstimulated vs. THR 1U/ml	0.8681
unstimulated vs. aPC 10ug/ml	0.8681
sec 138	
unstimulated vs. THR 1U/ml	0.7764
unstimulated vs. aPC 10ug/ml	0.8816
sec 139	
unstimulated vs. THR 1U/ml	0.8362
unstimulated vs. aPC 10ug/ml	0.9487
sec 140	
unstimulated vs. THR 1U/ml	0.8755
unstimulated vs. aPC 10ug/ml	0.9828
sec 141	
unstimulated vs. THR 1U/ml	0.8028
unstimulated vs. aPC 10ug/ml	0.9161
sec 142	
unstimulated vs. THR 1U/ml	0.953
unstimulated vs. aPC 10ug/ml	0.953
sec 143	
unstimulated vs. THR 1U/ml	0.9002
unstimulated vs. aPC 10ug/ml	0.9383
sec 144	
unstimulated vs. THR 1U/ml	0.9574
unstimulated vs. aPC 10ug/ml	0.9574
sec 145	
unstimulated vs. THR 1U/ml	0.8351
unstimulated vs. aPC 10ug/ml	0.893
sec 146	
unstimulated vs. THR 1U/ml	0.8789
unstimulated vs. aPC 10ug/ml	0.9632
sec 147	
unstimulated vs. THR 1U/ml	0.8844
unstimulated vs. aPC 10ug/ml	0.8983
sec 148	
unstimulated vs. THR 1U/ml	0.9237
unstimulated vs. aPC 10ug/ml	0.9237
sec 149	
unstimulated vs. THR 1U/ml	0.8748
unstimulated vs. aPC 10ug/ml	0.9591

sec 150	
unstimulated vs. THR 1U/ml	0.8436
unstimulated vs. aPC 10ug/ml	0.9129
sec 151	
unstimulated vs. THR 1U/ml	0.7989
unstimulated vs. aPC 10ug/ml	0.7989
sec 152	
unstimulated vs. THR 1U/ml	0.8498
unstimulated vs. aPC 10ug/ml	0.9114
sec 153	
unstimulated vs. THR 1U/ml	0.7887
unstimulated vs. aPC 10ug/ml	0.9716
sec 154	
unstimulated vs. THR 1U/ml	0.9022
unstimulated vs. aPC 10ug/ml	0.9022
sec 155	
unstimulated vs. THR 1U/ml	0.7491
unstimulated vs. aPC 10ug/ml	0.8952
sec 156	
unstimulated vs. THR 1U/ml	0.9258
unstimulated vs. aPC 10ug/ml	0.9613
sec 157	
unstimulated vs. THR 1U/ml	0.9582
unstimulated vs. aPC 10ug/ml	0.9582
sec 158	
unstimulated vs. THR 1U/ml	0.7918
unstimulated vs. aPC 10ug/ml	0.9754
sec 159	
unstimulated vs. THR 1U/ml	0.882
unstimulated vs. aPC 10ug/ml	0.882
sec 160	
unstimulated vs. THR 1U/ml	0.9312
unstimulated vs. aPC 10ug/ml	0.9999
sec 161	
unstimulated vs. THR 1U/ml	0.8286
unstimulated vs. aPC 10ug/ml	0.9336
sec 162	
unstimulated vs. THR 1U/ml	0.9115
unstimulated vs. aPC 10ug/ml	0.9115
sec 163	
unstimulated vs. THR 1U/ml	0.8774
unstimulated vs. aPC 10ug/ml	0.8774
sec 164	
unstimulated vs. THR 1U/ml	0.9369
unstimulated vs. aPC 10ug/ml	0.9909

sec 165	
unstimulated vs. THR 1U/ml	0.7204
unstimulated vs. aPC 10ug/ml	0.7823
sec 166	
unstimulated vs. THR 1U/ml	0.822
unstimulated vs. aPC 10ug/ml	0.8272
sec 167	
unstimulated vs. THR 1U/ml	0.9101
unstimulated vs. aPC 10ug/ml	0.9849
sec 168	
unstimulated vs. THR 1U/ml	0.8821
unstimulated vs. aPC 10ug/ml	0.9266
sec 169	
unstimulated vs. THR 1U/ml	0.8778
unstimulated vs. aPC 10ug/ml	0.8778
sec 170	
unstimulated vs. THR 1U/ml	0.8795
unstimulated vs. aPC 10ug/ml	0.9835
sec 171	
unstimulated vs. THR 1U/ml	0.8397
unstimulated vs. aPC 10ug/ml	0.9931
sec 172	
unstimulated vs. THR 1U/ml	0.8754
unstimulated vs. aPC 10ug/ml	0.8747
sec 173	
unstimulated vs. THR 1U/ml	0.8528
unstimulated vs. aPC 10ug/ml	0.8528
sec 174	
unstimulated vs. THR 1U/ml	0.899
unstimulated vs. aPC 10ug/ml	0.975
sec 175	
unstimulated vs. THR 1U/ml	0.7701
unstimulated vs. aPC 10ug/ml	0.7939
sec 176	
unstimulated vs. THR 1U/ml	0.8386
unstimulated vs. aPC 10ug/ml	0.8897
sec 177	
unstimulated vs. THR 1U/ml	0.8419
unstimulated vs. aPC 10ug/ml	0.9872
sec 178	
unstimulated vs. THR 1U/ml	0.8568
unstimulated vs. aPC 10ug/ml	0.9036
sec 179	
unstimulated vs. THR 1U/ml	0.7185
unstimulated vs. aPC 10ug/ml	0.7185

sec 180	
unstimulated vs. THR 1U/ml	0.7464
unstimulated vs. aPC 10ug/ml	0.8359

Fig. 2A bar chart	
Tukey's multiple comparisons test	Adjusted P Value
THR vs. control	<0.0001
THR vs. aPC	<0.0001
control vs. aPC	0.9987

Fig. 2B	
Unpaired t test	P value
EPCR- vs EPCR+	0.3246

Fig. 2C kinetics	
Holm-Šídák's multiple comparisons test	Adjusted P Value
sec 1	
DMSO vs. DMSO THR	0.8354
DMSO vs. PM2	0.8354
DMSO vs. PM2 10uM THR 1U/ml	0.8354
sec 2	
DMSO vs. DMSO THR	0.7916
DMSO vs. PM2	0.9068
DMSO vs. PM2 10uM THR 1U/ml	0.9068
sec 3	
DMSO vs. DMSO THR	0.9717
DMSO vs. PM2	0.9639
DMSO vs. PM2 10uM THR 1U/ml	0.9717
sec 4	
DMSO vs. DMSO THR	0.9963
DMSO vs. PM2	0.9705
DMSO vs. PM2 10uM THR 1U/ml	0.9963
sec 5	
DMSO vs. DMSO THR	0.9987
DMSO vs. PM2	0.9987
DMSO vs. PM2 10uM THR 1U/ml	0.9987
sec 6	
DMSO vs. DMSO THR	0.9897
DMSO vs. PM2	0.9897
DMSO vs. PM2 10uM THR 1U/ml	0.9897
sec 7	
DMSO vs. DMSO THR	0.7916
DMSO vs. PM2	0.7993
DMSO vs. PM2 10uM THR 1U/ml	0.7385
sec 8	
DMSO vs. DMSO THR	0.5805
DMSO vs. PM2	0.7152
DMSO vs. PM2 10uM THR 1U/ml	0.7152
sec 9	
DMSO vs. DMSO THR	0.8695

DMSO vs. PM2	0.9971
DMSO vs. PM2 10uM THR 1U/ml	0.8695
sec 10	
DMSO vs. DMSO THR	0.7627
DMSO vs. PM2	0.9045
DMSO vs. PM2 10uM THR 1U/ml	0.7627
sec 11	
DMSO vs. DMSO THR	0.9261
DMSO vs. PM2	0.9261
DMSO vs. PM2 10uM THR 1U/ml	0.9261
sec 12	
DMSO vs. DMSO THR	0.9365
DMSO vs. PM2	0.943
DMSO vs. PM2 10uM THR 1U/ml	0.924
sec 13	
DMSO vs. DMSO THR	0.9067
DMSO vs. PM2	0.9067
DMSO vs. PM2 10uM THR 1U/ml	0.9067
sec 14	
DMSO vs. DMSO THR	0.9476
DMSO vs. PM2	0.9476
DMSO vs. PM2 10uM THR 1U/ml	0.9476
sec 15	
DMSO vs. DMSO THR	0.9466
DMSO vs. PM2	0.9964
DMSO vs. PM2 10uM THR 1U/ml	0.7839
sec 16	
DMSO vs. DMSO THR	0.9361
DMSO vs. PM2	0.97
DMSO vs. PM2 10uM THR 1U/ml	0.9361
sec 17	
DMSO vs. DMSO THR	0.9531
DMSO vs. PM2	0.9531
DMSO vs. PM2 10uM THR 1U/ml	0.9531
sec 18	
DMSO vs. DMSO THR	0.9922
DMSO vs. PM2	0.9922
DMSO vs. PM2 10uM THR 1U/ml	0.879
sec 19	
DMSO vs. DMSO THR	0.9372
DMSO vs. PM2	0.9372
DMSO vs. PM2 10uM THR 1U/ml	0.9372
sec 20	
DMSO vs. DMSO THR	0.9557
DMSO vs. PM2	0.9557
DMSO vs. PM2 10uM THR 1U/ml	0.9557
sec 21	
DMSO vs. DMSO THR	0.9645

DMSO vs. PM2	0.9645
DMSO vs. PM2 10uM THR 1U/ml	0.9645
sec 22	
DMSO vs. DMSO THR	0.9162
DMSO vs. PM2	0.9162
DMSO vs. PM2 10uM THR 1U/ml	0.9162
sec 23	
DMSO vs. DMSO THR	0.9345
DMSO vs. PM2	0.9857
DMSO vs. PM2 10uM THR 1U/ml	0.9345
sec 24	
DMSO vs. DMSO THR	0.8467
DMSO vs. PM2	0.9921
DMSO vs. PM2 10uM THR 1U/ml	0.8467
sec 25	
DMSO vs. DMSO THR	0.8719
DMSO vs. PM2	0.9425
DMSO vs. PM2 10uM THR 1U/ml	0.837
sec 26	
DMSO vs. DMSO THR	0.7599
DMSO vs. PM2	0.7599
DMSO vs. PM2 10uM THR 1U/ml	0.6744
sec 27	
DMSO vs. DMSO THR	0.716
DMSO vs. PM2	0.9709
DMSO vs. PM2 10uM THR 1U/ml	0.7418
sec 28	
DMSO vs. DMSO THR	0.965
DMSO vs. PM2	0.9886
DMSO vs. PM2 10uM THR 1U/ml	0.965
sec 29	
DMSO vs. DMSO THR	0.7459
DMSO vs. PM2	0.8898
DMSO vs. PM2 10uM THR 1U/ml	0.7459
sec 30	
DMSO vs. DMSO THR	0.8409
DMSO vs. PM2	0.9873
DMSO vs. PM2 10uM THR 1U/ml	0.8134
sec 31	
DMSO vs. DMSO THR	0.8653
DMSO vs. PM2	0.8653
DMSO vs. PM2 10uM THR 1U/ml	0.8653
sec 32	
DMSO vs. DMSO THR	0.8779
DMSO vs. PM2	0.886
DMSO vs. PM2 10uM THR 1U/ml	0.8779
sec 33	
DMSO vs. DMSO THR	0.95

DMSO vs. PM2	0.95
DMSO vs. PM2 10uM THR 1U/ml	0.95
sec 34	
DMSO vs. DMSO THR	0.6861
DMSO vs. PM2	0.8905
DMSO vs. PM2 10uM THR 1U/ml	0.6861
sec 35	
DMSO vs. DMSO THR	0.8329
DMSO vs. PM2	0.8695
DMSO vs. PM2 10uM THR 1U/ml	0.8307
sec 36	
DMSO vs. DMSO THR	0.9276
DMSO vs. PM2	0.9276
DMSO vs. PM2 10uM THR 1U/ml	0.9276
sec 37	
DMSO vs. DMSO THR	0.7
DMSO vs. PM2	0.8888
DMSO vs. PM2 10uM THR 1U/ml	0.7261
sec 38	
DMSO vs. DMSO THR	0.3945
DMSO vs. PM2	0.8671
DMSO vs. PM2 10uM THR 1U/ml	0.816
sec 39	
DMSO vs. DMSO THR	0.6464
DMSO vs. PM2	0.7133
DMSO vs. PM2 10uM THR 1U/ml	0.7133
sec 40	
DMSO vs. DMSO THR	0.0832
DMSO vs. PM2	0.8842
DMSO vs. PM2 10uM THR 1U/ml	0.5218
sec 41	
DMSO vs. DMSO THR	0.0349
DMSO vs. PM2	0.9348
DMSO vs. PM2 10uM THR 1U/ml	0.7025
sec 42	
DMSO vs. DMSO THR	0.0005
DMSO vs. PM2	0.8837
DMSO vs. PM2 10uM THR 1U/ml	0.6638
sec 43	
DMSO vs. DMSO THR	<0.0001
DMSO vs. PM2	0.9381
DMSO vs. PM2 10uM THR 1U/ml	0.5236
sec 44	
DMSO vs. DMSO THR	<0.0001
DMSO vs. PM2	0.9186
DMSO vs. PM2 10uM THR 1U/ml	0.5921
sec 45	
DMSO vs. DMSO THR	<0.0001

DMSO vs. PM2	0.9779
DMSO vs. PM2 10uM THR 1U/ml	0.7312
sec 46	
DMSO vs. DMSO THR	<0.0001
DMSO vs. PM2	0.787
DMSO vs. PM2 10uM THR 1U/ml	0.6725
sec 47	
DMSO vs. DMSO THR	<0.0001
DMSO vs. PM2	0.9387
DMSO vs. PM2 10uM THR 1U/ml	0.8298
sec 48	
DMSO vs. DMSO THR	<0.0001
DMSO vs. PM2	0.949
DMSO vs. PM2 10uM THR 1U/ml	0.802
sec 49	
DMSO vs. DMSO THR	<0.0001
DMSO vs. PM2	0.9743
DMSO vs. PM2 10uM THR 1U/ml	0.6242
sec 50	
DMSO vs. DMSO THR	<0.0001
DMSO vs. PM2	0.9479
DMSO vs. PM2 10uM THR 1U/ml	0.2561
sec 51	
DMSO vs. DMSO THR	<0.0001
DMSO vs. PM2	0.9568
DMSO vs. PM2 10uM THR 1U/ml	0.7023
sec 52	
DMSO vs. DMSO THR	<0.0001
DMSO vs. PM2	0.9175
DMSO vs. PM2 10uM THR 1U/ml	0.6971
sec 53	
DMSO vs. DMSO THR	<0.0001
DMSO vs. PM2	0.9615
DMSO vs. PM2 10uM THR 1U/ml	0.3083
sec 54	
DMSO vs. DMSO THR	<0.0001
DMSO vs. PM2	0.8207
DMSO vs. PM2 10uM THR 1U/ml	0.4719
sec 55	
DMSO vs. DMSO THR	<0.0001
DMSO vs. PM2	0.8921
DMSO vs. PM2 10uM THR 1U/ml	0.7327
sec 56	
DMSO vs. DMSO THR	<0.0001
DMSO vs. PM2	0.9584
DMSO vs. PM2 10uM THR 1U/ml	0.5163
sec 57	
DMSO vs. DMSO THR	<0.0001

DMSO vs. PM2	0.9721
DMSO vs. PM2 10uM THR 1U/ml	0.4338
sec 58	
DMSO vs. DMSO THR	<0.0001
DMSO vs. PM2	0.784
DMSO vs. PM2 10uM THR 1U/ml	0.6422
sec 59	
DMSO vs. DMSO THR	<0.0001
DMSO vs. PM2	0.8495
DMSO vs. PM2 10uM THR 1U/ml	0.4113
sec 60	
DMSO vs. DMSO THR	<0.0001
DMSO vs. PM2	0.9218
DMSO vs. PM2 10uM THR 1U/ml	0.8069
sec 61	
DMSO vs. DMSO THR	<0.0001
DMSO vs. PM2	0.9119
DMSO vs. PM2 10uM THR 1U/ml	0.849
sec 62	
DMSO vs. DMSO THR	<0.0001
DMSO vs. PM2	0.8446
DMSO vs. PM2 10uM THR 1U/ml	0.8446
sec 63	
DMSO vs. DMSO THR	<0.0001
DMSO vs. PM2	0.8106
DMSO vs. PM2 10uM THR 1U/ml	0.6405
sec 64	
DMSO vs. DMSO THR	<0.0001
DMSO vs. PM2	0.8226
DMSO vs. PM2 10uM THR 1U/ml	0.5491
sec 65	
DMSO vs. DMSO THR	<0.0001
DMSO vs. PM2	0.9145
DMSO vs. PM2 10uM THR 1U/ml	0.8411
sec 66	
DMSO vs. DMSO THR	<0.0001
DMSO vs. PM2	0.9019
DMSO vs. PM2 10uM THR 1U/ml	0.8465
sec 67	
DMSO vs. DMSO THR	<0.0001
DMSO vs. PM2	0.9944
DMSO vs. PM2 10uM THR 1U/ml	0.853
sec 68	
DMSO vs. DMSO THR	<0.0001
DMSO vs. PM2	0.9308
DMSO vs. PM2 10uM THR 1U/ml	0.8222
sec 69	
DMSO vs. DMSO THR	<0.0001

DMSO vs. PM2	0.874
DMSO vs. PM2 10uM THR 1U/ml	0.6162
sec 70	
DMSO vs. DMSO THR	<0.0001
DMSO vs. PM2	0.7878
DMSO vs. PM2 10uM THR 1U/ml	0.7406
sec 71	
DMSO vs. DMSO THR	<0.0001
DMSO vs. PM2	0.9868
DMSO vs. PM2 10uM THR 1U/ml	0.6347
sec 72	
DMSO vs. DMSO THR	<0.0001
DMSO vs. PM2	0.9749
DMSO vs. PM2 10uM THR 1U/ml	0.777
sec 73	
DMSO vs. DMSO THR	<0.0001
DMSO vs. PM2	0.8296
DMSO vs. PM2 10uM THR 1U/ml	0.5254
sec 74	
DMSO vs. DMSO THR	<0.0001
DMSO vs. PM2	0.8236
DMSO vs. PM2 10uM THR 1U/ml	0.5113
sec 75	
DMSO vs. DMSO THR	<0.0001
DMSO vs. PM2	0.949
DMSO vs. PM2 10uM THR 1U/ml	0.9474
sec 76	
DMSO vs. DMSO THR	<0.0001
DMSO vs. PM2	0.8506
DMSO vs. PM2 10uM THR 1U/ml	0.6759
sec 77	
DMSO vs. DMSO THR	<0.0001
DMSO vs. PM2	0.9465
DMSO vs. PM2 10uM THR 1U/ml	0.9043
sec 78	
DMSO vs. DMSO THR	<0.0001
DMSO vs. PM2	0.8323
DMSO vs. PM2 10uM THR 1U/ml	0.6885
sec 79	
DMSO vs. DMSO THR	<0.0001
DMSO vs. PM2	0.9807
DMSO vs. PM2 10uM THR 1U/ml	0.8674
sec 80	
DMSO vs. DMSO THR	<0.0001
DMSO vs. PM2	0.9325
DMSO vs. PM2 10uM THR 1U/ml	0.9325
sec 81	
DMSO vs. DMSO THR	<0.0001

DMSO vs. PM2	0.9977
DMSO vs. PM2 10uM THR 1U/ml	0.8597
sec 82	
DMSO vs. DMSO THR	0.0003
DMSO vs. PM2	0.8797
DMSO vs. PM2 10uM THR 1U/ml	0.8797
sec 83	
DMSO vs. DMSO THR	<0.0001
DMSO vs. PM2	0.8884
DMSO vs. PM2 10uM THR 1U/ml	0.8492
sec 84	
DMSO vs. DMSO THR	<0.0001
DMSO vs. PM2	0.9832
DMSO vs. PM2 10uM THR 1U/ml	0.8749
sec 85	
DMSO vs. DMSO THR	0.0008
DMSO vs. PM2	0.9957
DMSO vs. PM2 10uM THR 1U/ml	0.8894
sec 86	
DMSO vs. DMSO THR	0.0013
DMSO vs. PM2	0.8948
DMSO vs. PM2 10uM THR 1U/ml	0.6857
sec 87	
DMSO vs. DMSO THR	0.0028
DMSO vs. PM2	0.9403
DMSO vs. PM2 10uM THR 1U/ml	0.7612
sec 88	
DMSO vs. DMSO THR	0.0014
DMSO vs. PM2	0.983
DMSO vs. PM2 10uM THR 1U/ml	0.847
sec 89	
DMSO vs. DMSO THR	0.0045
DMSO vs. PM2	0.9304
DMSO vs. PM2 10uM THR 1U/ml	0.7304
sec 90	
DMSO vs. DMSO THR	0.0203
DMSO vs. PM2	0.9752
DMSO vs. PM2 10uM THR 1U/ml	0.9752
sec 91	
DMSO vs. DMSO THR	0.0152
DMSO vs. PM2	0.8828
DMSO vs. PM2 10uM THR 1U/ml	0.8828
sec 92	
DMSO vs. DMSO THR	0.0159
DMSO vs. PM2	0.9571
DMSO vs. PM2 10uM THR 1U/ml	0.9314
sec 93	
DMSO vs. DMSO THR	0.0172

DMSO vs. PM2	0.879
DMSO vs. PM2 10uM THR 1U/ml	0.8682
sec 94	
DMSO vs. DMSO THR	0.0147
DMSO vs. PM2	0.9527
DMSO vs. PM2 10uM THR 1U/ml	0.9527
sec 95	
DMSO vs. DMSO THR	0.0453
DMSO vs. PM2	0.9797
DMSO vs. PM2 10uM THR 1U/ml	0.7469
sec 96	
DMSO vs. DMSO THR	0.0556
DMSO vs. PM2	0.9163
DMSO vs. PM2 10uM THR 1U/ml	0.9163
sec 97	
DMSO vs. DMSO THR	0.1354
DMSO vs. PM2	0.9753
DMSO vs. PM2 10uM THR 1U/ml	0.9753
sec 98	
DMSO vs. DMSO THR	0.3313
DMSO vs. PM2	0.9391
DMSO vs. PM2 10uM THR 1U/ml	0.9391
sec 99	
DMSO vs. DMSO THR	0.1044
DMSO vs. PM2	0.9761
DMSO vs. PM2 10uM THR 1U/ml	0.8421
sec 100	
DMSO vs. DMSO THR	0.1373
DMSO vs. PM2	0.9401
DMSO vs. PM2 10uM THR 1U/ml	0.86
sec 101	
DMSO vs. DMSO THR	0.3116
DMSO vs. PM2	0.8624
DMSO vs. PM2 10uM THR 1U/ml	0.8029
sec 102	
DMSO vs. DMSO THR	0.2433
DMSO vs. PM2	0.8268
DMSO vs. PM2 10uM THR 1U/ml	0.5711
sec 103	
DMSO vs. DMSO THR	0.2701
DMSO vs. PM2	0.9775
DMSO vs. PM2 10uM THR 1U/ml	0.8308
sec 104	
DMSO vs. DMSO THR	0.2798
DMSO vs. PM2	0.9749
DMSO vs. PM2 10uM THR 1U/ml	0.837
sec 105	
DMSO vs. DMSO THR	0.2658

DMSO vs. PM2	0.9031
DMSO vs. PM2 10uM THR 1U/ml	0.8187
sec 106	
DMSO vs. DMSO THR	0.471
DMSO vs. PM2	0.8248
DMSO vs. PM2 10uM THR 1U/ml	0.8189
sec 107	
DMSO vs. DMSO THR	0.51
DMSO vs. PM2	0.9734
DMSO vs. PM2 10uM THR 1U/ml	0.9085
sec 108	
DMSO vs. DMSO THR	0.4047
DMSO vs. PM2	0.9285
DMSO vs. PM2 10uM THR 1U/ml	0.7545
sec 109	
DMSO vs. DMSO THR	0.3094
DMSO vs. PM2	0.9701
DMSO vs. PM2 10uM THR 1U/ml	0.9256
sec 110	
DMSO vs. DMSO THR	0.369
DMSO vs. PM2	0.8861
DMSO vs. PM2 10uM THR 1U/ml	0.8861
sec 111	
DMSO vs. DMSO THR	0.5902
DMSO vs. PM2	0.8779
DMSO vs. PM2 10uM THR 1U/ml	0.8779
sec 112	
DMSO vs. DMSO THR	0.5488
DMSO vs. PM2	0.9153
DMSO vs. PM2 10uM THR 1U/ml	0.8835
sec 113	
DMSO vs. DMSO THR	0.7843
DMSO vs. PM2	0.7843
DMSO vs. PM2 10uM THR 1U/ml	0.7843
sec 114	
DMSO vs. DMSO THR	0.3611
DMSO vs. PM2	0.8346
DMSO vs. PM2 10uM THR 1U/ml	0.7308
sec 115	
DMSO vs. DMSO THR	0.6454
DMSO vs. PM2	0.9799
DMSO vs. PM2 10uM THR 1U/ml	0.9093
sec 116	
DMSO vs. DMSO THR	0.6775
DMSO vs. PM2	0.9924
DMSO vs. PM2 10uM THR 1U/ml	0.9241
sec 117	
DMSO vs. DMSO THR	0.7673

DMSO vs. PM2	0.9206
DMSO vs. PM2 10uM THR 1U/ml	0.9206
sec 118	
DMSO vs. DMSO THR	0.5977
DMSO vs. PM2	0.9402
DMSO vs. PM2 10uM THR 1U/ml	0.7555
sec 119	
DMSO vs. DMSO THR	0.7574
DMSO vs. PM2	0.9321
DMSO vs. PM2 10uM THR 1U/ml	0.9321
sec 120	
DMSO vs. DMSO THR	0.6694
DMSO vs. PM2	0.883
DMSO vs. PM2 10uM THR 1U/ml	0.8738
sec 121	
DMSO vs. DMSO THR	0.7276
DMSO vs. PM2	0.9713
DMSO vs. PM2 10uM THR 1U/ml	0.8252
sec 122	
DMSO vs. DMSO THR	0.9243
DMSO vs. PM2	0.9243
DMSO vs. PM2 10uM THR 1U/ml	0.9243
sec 123	
DMSO vs. DMSO THR	0.918
DMSO vs. PM2	0.918
DMSO vs. PM2 10uM THR 1U/ml	0.918
sec 124	
DMSO vs. DMSO THR	0.9086
DMSO vs. PM2	0.9495
DMSO vs. PM2 10uM THR 1U/ml	0.9165
sec 125	
DMSO vs. DMSO THR	0.6555
DMSO vs. PM2	0.8015
DMSO vs. PM2 10uM THR 1U/ml	0.8015
sec 126	
DMSO vs. DMSO THR	0.8556
DMSO vs. PM2	0.9119
DMSO vs. PM2 10uM THR 1U/ml	0.9119
sec 127	
DMSO vs. DMSO THR	0.9857
DMSO vs. PM2	0.9857
DMSO vs. PM2 10uM THR 1U/ml	0.9857
sec 128	
DMSO vs. DMSO THR	0.9672
DMSO vs. PM2	0.9831
DMSO vs. PM2 10uM THR 1U/ml	0.9831
sec 129	
DMSO vs. DMSO THR	0.8408

DMSO vs. PM2	0.9205
DMSO vs. PM2 10uM THR 1U/ml	0.8408
sec 130	
DMSO vs. DMSO THR	0.7751
DMSO vs. PM2	0.9712
DMSO vs. PM2 10uM THR 1U/ml	0.9447
sec 131	
DMSO vs. DMSO THR	0.8422
DMSO vs. PM2	0.967
DMSO vs. PM2 10uM THR 1U/ml	0.967
sec 132	
DMSO vs. DMSO THR	0.9334
DMSO vs. PM2	0.9798
DMSO vs. PM2 10uM THR 1U/ml	0.9798
sec 133	
DMSO vs. DMSO THR	0.9554
DMSO vs. PM2	0.9554
DMSO vs. PM2 10uM THR 1U/ml	0.9554
sec 134	
DMSO vs. DMSO THR	0.9825
DMSO vs. PM2	0.9934
DMSO vs. PM2 10uM THR 1U/ml	0.9825
sec 135	
DMSO vs. DMSO THR	0.974
DMSO vs. PM2	0.9751
DMSO vs. PM2 10uM THR 1U/ml	0.9751
sec 136	
DMSO vs. DMSO THR	0.9308
DMSO vs. PM2	0.9544
DMSO vs. PM2 10uM THR 1U/ml	0.9308
sec 137	
DMSO vs. DMSO THR	0.951
DMSO vs. PM2	0.951
DMSO vs. PM2 10uM THR 1U/ml	0.951
sec 138	
DMSO vs. DMSO THR	0.9581
DMSO vs. PM2	0.9581
DMSO vs. PM2 10uM THR 1U/ml	0.9581
sec 139	
DMSO vs. DMSO THR	0.9562
DMSO vs. PM2	0.9562
DMSO vs. PM2 10uM THR 1U/ml	0.9341
sec 140	
DMSO vs. DMSO THR	0.9871
DMSO vs. PM2	0.9871
DMSO vs. PM2 10uM THR 1U/ml	0.9871
sec 141	
DMSO vs. DMSO THR	0.9611

DMSO vs. PM2	0.9611
DMSO vs. PM2 10uM THR 1U/ml	0.9611
sec 142	
DMSO vs. DMSO THR	0.9875
DMSO vs. PM2	0.9875
DMSO vs. PM2 10uM THR 1U/ml	0.9875
sec 143	
DMSO vs. DMSO THR	0.8912
DMSO vs. PM2	0.8912
DMSO vs. PM2 10uM THR 1U/ml	0.8912
sec 144	
DMSO vs. DMSO THR	0.926
DMSO vs. PM2	0.926
DMSO vs. PM2 10uM THR 1U/ml	0.876
sec 145	
DMSO vs. DMSO THR	0.9636
DMSO vs. PM2	0.9636
DMSO vs. PM2 10uM THR 1U/ml	0.9636
sec 146	
DMSO vs. DMSO THR	0.9635
DMSO vs. PM2	0.9782
DMSO vs. PM2 10uM THR 1U/ml	0.9635
sec 147	
DMSO vs. DMSO THR	0.9923
DMSO vs. PM2	0.9923
DMSO vs. PM2 10uM THR 1U/ml	0.9923
sec 148	
DMSO vs. DMSO THR	0.8882
DMSO vs. PM2	0.9307
DMSO vs. PM2 10uM THR 1U/ml	0.9123
sec 149	
DMSO vs. DMSO THR	0.9138
DMSO vs. PM2	0.9879
DMSO vs. PM2 10uM THR 1U/ml	0.9796
sec 150	
DMSO vs. DMSO THR	0.9233
DMSO vs. PM2	0.984
DMSO vs. PM2 10uM THR 1U/ml	0.9173
sec 151	
DMSO vs. DMSO THR	0.9237
DMSO vs. PM2	0.9629
DMSO vs. PM2 10uM THR 1U/ml	0.9237
sec 152	
DMSO vs. DMSO THR	0.9058
DMSO vs. PM2	0.9377
DMSO vs. PM2 10uM THR 1U/ml	0.843
sec 153	
DMSO vs. DMSO THR	0.9155

DMSO vs. PM2	0.9155
DMSO vs. PM2 10uM THR 1U/ml	0.9155
sec 154	
DMSO vs. DMSO THR	0.9685
DMSO vs. PM2	0.9685
DMSO vs. PM2 10uM THR 1U/ml	0.9685
sec 155	
DMSO vs. DMSO THR	0.9802
DMSO vs. PM2	0.9942
DMSO vs. PM2 10uM THR 1U/ml	0.9942
sec 156	
DMSO vs. DMSO THR	0.9756
DMSO vs. PM2	0.9756
DMSO vs. PM2 10uM THR 1U/ml	0.9756
sec 157	
DMSO vs. DMSO THR	0.9956
DMSO vs. PM2	0.9956
DMSO vs. PM2 10uM THR 1U/ml	0.9956
sec 158	
DMSO vs. DMSO THR	0.9396
DMSO vs. PM2	0.9572
DMSO vs. PM2 10uM THR 1U/ml	0.9396
sec 159	
DMSO vs. DMSO THR	0.9797
DMSO vs. PM2	0.9797
DMSO vs. PM2 10uM THR 1U/ml	0.9797
sec 160	
DMSO vs. DMSO THR	0.9903
DMSO vs. PM2	0.9903
DMSO vs. PM2 10uM THR 1U/ml	0.9903
sec 161	
DMSO vs. DMSO THR	0.9397
DMSO vs. PM2	0.9721
DMSO vs. PM2 10uM THR 1U/ml	0.9721
sec 162	
DMSO vs. DMSO THR	0.9416
DMSO vs. PM2	0.9416
DMSO vs. PM2 10uM THR 1U/ml	0.9416
sec 163	
DMSO vs. DMSO THR	0.9412
DMSO vs. PM2	0.9412
DMSO vs. PM2 10uM THR 1U/ml	0.9412
sec 164	
DMSO vs. DMSO THR	0.9897
DMSO vs. PM2	0.9897
DMSO vs. PM2 10uM THR 1U/ml	0.9897
sec 165	
DMSO vs. DMSO THR	0.9698

DMSO vs. PM2	0.9698
DMSO vs. PM2 10uM THR 1U/ml	0.8866
sec 166	
DMSO vs. DMSO THR	0.9812
DMSO vs. PM2	0.9812
DMSO vs. PM2 10uM THR 1U/ml	0.9812
sec 167	
DMSO vs. DMSO THR	0.9967
DMSO vs. PM2	0.9967
DMSO vs. PM2 10uM THR 1U/ml	0.9984
sec 168	
DMSO vs. DMSO THR	0.9717
DMSO vs. PM2	0.9717
DMSO vs. PM2 10uM THR 1U/ml	0.8913
sec 169	
DMSO vs. DMSO THR	0.9556
DMSO vs. PM2	0.9556
DMSO vs. PM2 10uM THR 1U/ml	0.9516
sec 170	
DMSO vs. DMSO THR	0.8679
DMSO vs. PM2	0.8679
DMSO vs. PM2 10uM THR 1U/ml	0.867
sec 171	
DMSO vs. DMSO THR	0.8993
DMSO vs. PM2	0.8993
DMSO vs. PM2 10uM THR 1U/ml	0.8993
sec 172	
DMSO vs. DMSO THR	0.9385
DMSO vs. PM2	0.9385
DMSO vs. PM2 10uM THR 1U/ml	0.9385
sec 173	
DMSO vs. DMSO THR	0.937
DMSO vs. PM2	0.937
DMSO vs. PM2 10uM THR 1U/ml	0.937
sec 174	
DMSO vs. DMSO THR	0.8424
DMSO vs. PM2	0.901
DMSO vs. PM2 10uM THR 1U/ml	0.8651
sec 175	
DMSO vs. DMSO THR	0.9554
DMSO vs. PM2	0.9554
DMSO vs. PM2 10uM THR 1U/ml	0.9554
sec 176	
DMSO vs. DMSO THR	0.9565
DMSO vs. PM2	0.9565
DMSO vs. PM2 10uM THR 1U/ml	0.9565
sec 177	
DMSO vs. DMSO THR	0.9966

DMSO vs. PM2	0.9978
DMSO vs. PM2 10uM THR 1U/ml	0.9978
sec 178	
DMSO vs. DMSO THR	0.9447
DMSO vs. PM2	0.9447
DMSO vs. PM2 10uM THR 1U/ml	0.9447
sec 179	
DMSO vs. DMSO THR	0.8612
DMSO vs. PM2	0.8612
DMSO vs. PM2 10uM THR 1U/ml	0.8612
sec 180	
DMSO vs. DMSO THR	0.8876
DMSO vs. PM2	0.9364
DMSO vs. PM2 10uM THR 1U/ml	0.9364

Fig. 2C bar chart

Tukey's multiple comparisons test	Adjusted P Value
THR vs. DMSO	<0.0001
THR vs. PM2+THR	<0.0001
THR vs. PM2	<0.0001
DMSO vs. PM2+THR	0.9691
DMSO vs. PM2	>0.9999
PM2+THR vs. PM2	0.9722

Fig. 2D

Unpaired t test	P value
CD90+ vs. CD90-	0.0299

Fig. 2E

Unpaired t test	P value
EPCR+ vs. EPCR-	0.9785

Fig. 2F

Holm-Šídák's multiple comparisons test	Adjusted P Value
THR 1U/ml	
control vs. 10min	<0.0001
control vs. 30min	<0.0001
control vs. 60min	0.1958
control vs. 120min	0.1958
aPC 10µg/ml	
control vs. 10min	<0.0001
control vs. 30min	0.0345
control vs. 60min	0.0345
control vs. 120min	0.2935
PM2 10µM + THR 1U/ml	
control vs. 10min	0.0185
control vs. 30min	0.0286
control vs. 60min	0.1015
control vs. 120min	0.4142

Fig. 2G

Holm-Šídák's multiple comparisons test	Adjusted P Value

THR 1U/ml	
control vs. 10min	0.0692
control vs. 30min	0.0451
control vs. 60min	0.3082
control vs. 120min	0.3651
aPC 10µg/ml	
control vs. 10min	0.0366
control vs. 30min	<0.0001
control vs. 60min	<0.0001
control vs. 120min	0.4748
PM2 10µM + THR 1U/ml	
control vs. 10min	0.7622
control vs. 30min	0.7622
control vs. 60min	0.2347
control vs. 120min	0.5891

Fig. 2H

Holm-Šídák's multiple comparisons test	Adjusted P Value
THR 1U/ml	
control vs. 10min	0.295
control vs. 30min	0.0891
control vs. 60min	<0.0001
control vs. 120min	0.0002
aPC 10µg/ml	
control vs. 10min	0.995
control vs. 30min	0.7334
control vs. 60min	0.776
control vs. 120min	0.995
PM2 10µM + THR 1U/ml	
control vs. 10min	0.9059
control vs. 30min	0.4061
control vs. 60min	0.7083
control vs. 120min	0.9059

Fig. 2I

Holm-Šídák's multiple comparisons test	Adjusted P Value
THR 1U/ml	
control vs. 10min	0.1359
control vs. 30min	0.1359
control vs. 60min	0.2377
control vs. 120min	0.2249
aPC 10µg/ml	
control vs. 10min	0.1054
control vs. 30min	0.1033
control vs. 60min	0.2624
control vs. 120min	0.2624
PM2 10µM + THR 1U/ml	
control vs. 10min	0.0023
control vs. 30min	0.0023
control vs. 60min	0.0498
control vs. 120min	0.0498

Appendix Table S5: P values reported in Figure 4

Fig. 4B OCR	
Tukey's multiple comparisons test	Adjusted P Value
control vs. PM2	0.8442
control vs. THR	0.5194
control vs. PM2 + THR	0.9773
control vs. aPC	0.9955
PM2 vs. THR	0.9787
PM2 vs. PM2 + THR	0.5136
PM2 vs. aPC	0.637
THR vs. PM2 + THR	0.2263
THR vs. aPC	0.3132
PM2 + THR vs. aPC	0.9996

Fig. 4B ECAR	
Tukey's multiple comparisons test	Adjusted P Value
control vs. PM2	>0.9999
control vs. THR	0.0011
control vs. PM2 + THR	0.3476
control vs. aPC	0.9988
PM2 vs. THR	0.0013
PM2 vs. PM2 + THR	0.3947
PM2 vs. aPC	0.9963
THR vs. PM2 + THR	0.0921
THR vs. aPC	0.0005
PM2 + THR vs. aPC	0.2276

Fig. 4C	
Tukey's multiple comparisons test	Adjusted P Value
control vs. PM2	>0.9999
control vs. THR	0.7665
control vs. PM2 + THR	0.8009
control vs. aPC	0.2948
PM2 vs. THR	0.7119
PM2 vs. PM2 + THR	0.8478
PM2 vs. aPC	0.3413
THR vs. PM2 + THR	0.1846
THR vs. aPC	0.0296
PM2 + THR vs. aPC	0.8969

Fig 4D OCR	
Tukey's multiple comparisons test	Adjusted P Value
control vs. PM2	0.9949
control vs. THR	0.8251
control vs. PM2 + THR	0.7947
control vs. aPC	0.3715
PM2 vs. THR	0.6048
PM2 vs. PM2 + THR	0.9468
PM2 vs. aPC	0.5977
THR vs. PM2 + THR	0.2202
THR vs. aPC	0.0536
PM2 + THR vs. aPC	0.9485

Fig. 4D %	
Tukey's multiple comparisons test	Adjusted P Value
control vs. PM2	>0.9999
control vs. THR	0.2671
control vs. PM2 + THR	>0.9999

control vs. aPC	0.5508
PM2 vs. THR	0.3206
PM2 vs. PM2 + THR	>0.9999
PM2 vs. aPC	0.4805
THR vs. PM2 + THR	0.3225
THR vs. aPC	0.011
PM2 + THR vs. aPC	0.4781

Fig. 4F

Tukey's multiple comparisons test	Adjusted P Value
control vs. PM2	0.8837
control vs. THR	0.2968
control vs. PM2 + THR	0.0437
control vs. aPC	>0.9999
PM2 vs. THR	0.7977
PM2 vs. PM2 + THR	0.2189
PM2 vs. aPC	0.8353
THR vs. PM2 + THR	0.7911
THR vs. aPC	0.2514
PM2 + THR vs. aPC	0.0353

Fig. 4G

Tukey's multiple comparisons test	Adjusted P Value
control vs. PM2	0.8816
control vs. THR	0.8497
control vs. PM2 + THR	0.3688
control vs. aPC	0.9956
PM2 vs. THR	>0.9999
PM2 vs. PM2 + THR	0.8724
PM2 vs. aPC	0.697
THR vs. PM2 + THR	0.9016
THR vs. aPC	0.653
PM2 + THR vs. aPC	0.2154

Fig. 4H

Holm-Šídák's multiple comparisons test	Adjusted P Value
THR	
0 vs. 10	0.0435
0 vs. 30	0.9676
0 vs. 60	0.9253
aPC	
0 vs. 10	0.9652
0 vs. 30	0.6893
0 vs. 60	0.9233

Fig. 4I

Tukey's multiple comparisons test	Adjusted P Value
unstim vs. aPC	0.454
unstim vs. THR	0.0016
aPC vs. THR	0.0184

Appendix Table S6: P values reported in Figure 5

Fig. 5A CD34+ all colonies	
Tukey's multiple comparisons test	Adjusted P Value
DMSO vs. aPC	0.0002
DMSO vs. THR	0.1592
DMSO vs. PM2+THR	0.7819
aPC vs. THR	<0.0001
aPC vs. PM2+THR	0.0069
THR vs. PM2+THR	0.0138

Fig. 5A CD34- all colonies	
Tukey's multiple comparisons test	Adjusted P Value
DMSO vs. aPC	0.0359
DMSO vs. THR	0.6906
DMSO vs. PM2+THR	0.9416
aPC vs. THR	0.001
aPC vs. PM2+THR	0.0063
THR vs. PM2+THR	0.9505

Fig. 5B	
Tukey's multiple comparisons test	Adjusted P Value
DMSO vs. aPC	0.0008
DMSO vs. THR	0.9657
DMSO vs. PM2+THR	0.3526
aPC vs. THR	0.0001
aPC vs. PM2+THR	0.1188
THR vs. PM2+THR	0.1547

Fig. 5C 1st plating all colonies	
Tukey's multiple comparisons test	Adjusted P Value
unstimulated vs. THR 1U/ml	0.3178
unstimulated vs. aPC 10ug/ml	0.01
THR 1U/ml vs. aPC 10ug/ml	0.0022

Fig 5C 1st plating CFU-GM	
Tukey's multiple comparisons test	Adjusted P Value
unstimulated vs. aPC 10µg/ml	0.4084
unstimulated vs. THR	0.9858
aPC 10µg/ml vs. THR	0.3393

Fig 5C 1st plating CFU-E	
Tukey's multiple comparisons test	Adjusted P Value
unstimulated vs. aPC 10µg/ml	0.3217
unstimulated vs. THR	0.636
aPC 10µg/ml vs. THR	0.0986

Fig 5C 1st plating CFU-GEMM	
Tukey's multiple comparisons test	Adjusted P Value
unstimulated vs. aPC 10µg/ml	0.1603
unstimulated vs. THR	0.0515
aPC 10µg/ml vs. THR	0.0049

Fig 5C 2nd plating all colonies	
Tukey's multiple comparisons test	Adjusted P Value
unstimulated vs. THR 1U/ml	0.4623
unstimulated vs. aPC 10ug/ml	0.0297
THR 1U/ml vs. aPC 10ug/ml	0.0074

Fig. 5C 2nd plating CFU-GM	
Tukey's multiple comparisons test	Adjusted P Value
unstimulated vs. aPC 10µg/ml	0.1353
unstimulated vs. THR	0.2245
aPC 10µg/ml vs. THR	0.014

Fig 5C 2nd plating CFU-E	
Tukey's multiple comparisons test	Adjusted P Value
unstimulated vs. aPC 10µg/ml	0.5913
unstimulated vs. THR	0.7895
aPC 10µg/ml vs. THR	0.9341

Fig 5D	
Tukey's multiple comparisons test	Adjusted P Value
aPC vs. unstim	0.0004
aPC vs. THR	0.0029
unstim vs. THR	0.5928

Fig. 5E	
Tukey's multiple comparisons test	Adjusted P Value
THR vs. aPC	0.0051
THR vs. unstim	0.7963
aPC vs. unstim	0.0186

Fig. 5F	
Tukey's multiple comparisons test	Adjusted P Value
THR vs. aPC	0.9741
THR vs. unstim	0.505
aPC vs. unstim	0.635

Appendix Table S7: P values reported in Figure 6

Fig. 6A	
Tukey's multiple comparisons test	Adjusted P Value
IL-1α vs. THR	0.3585
IL-1α vs. aPC + IL-1α	0.8973
IL-1α vs. aPC	0.0007
IL-1α vs. unstim	0.4288
THR vs. aPC + IL-1α	0.8561
THR vs. aPC	0.0463
THR vs. unstim	>0.9999
aPC + IL-1α vs. aPC	0.0054
aPC + IL-1α vs. unstim	0.9085
aPC vs. unstim	0.0351

Fig. 6B	
Holm-Šídák's multiple comparisons test	Adjusted P Value
G0	
unstim vs. aPC	0.3846
unstim vs. THR	0.9088
unstim vs. IL-1α	0.8295
unstim vs. aPC + IL-1α	0.9088
aPC vs. THR	0.6679
aPC vs. IL-1α	0.0413
aPC vs. aPC + IL-1α	0.1724
THR vs. IL-1α	0.5897
THR vs. aPC + IL-1α	0.8295
IL-1α vs. aPC + IL-1α	0.9088
G1	
unstim vs. aPC	0.6798
unstim vs. THR	0.9135
unstim vs. IL-1α	0.8357
unstim vs. aPC + IL-1α	0.9135
aPC vs. THR	0.8357
aPC vs. IL-1α	0.1126
aPC vs. aPC + IL-1α	0.3395
THR vs. IL-1α	0.6059
THR vs. aPC + IL-1α	0.8357
IL-1α vs. aPC + IL-1α	0.9135
SG2M	
unstim vs. aPC	>0.9999
unstim vs. THR	>0.9999
unstim vs. IL-1α	>0.9999
unstim vs. aPC + IL-1α	>0.9999
aPC vs. THR	>0.9999
aPC vs. IL-1α	>0.9999
aPC vs. aPC + IL-1α	>0.9999
THR vs. IL-1α	>0.9999
THR vs. aPC + IL-1α	>0.9999
IL-1α vs. aPC + IL-1α	>0.9999

Fig. 6C	
Holm-Šídák's multiple comparisons test	Adjusted P Value
0	
unstimulated vs. aPC	0.9943

unstimulated vs. THR	0.9948
unstimulated vs. IL1	0.9936
unstimulated vs. aPC IL1	0.9943
aPC vs. THR	0.9943
aPC vs. IL1	0.8816
aPC vs. aPC IL1	0.9936
THR vs. IL1	0.9936
THR vs. aPC IL1	0.9943
IL1 vs. aPC IL1	0.9943
1	
unstimulated vs. aPC	0.0093
unstimulated vs. THR	0.4523
unstimulated vs. IL1	0.0036
unstimulated vs. aPC IL1	0.6934
aPC vs. THR	0.1272
aPC vs. IL1	<0.0001
aPC vs. aPC IL1	0.0036
THR vs. IL1	<0.0001
THR vs. aPC IL1	0.3424
IL1 vs. aPC IL1	0.0093
2	
unstimulated vs. aPC	0.0252
unstimulated vs. THR	0.544
unstimulated vs. IL1	0.3494
unstimulated vs. aPC IL1	0.9852
aPC vs. THR	0.3189
aPC vs. IL1	<0.0001
aPC vs. aPC IL1	0.0252
THR vs. IL1	0.0304
THR vs. aPC IL1	0.544
IL1 vs. aPC IL1	0.3494
3+	
unstimulated vs. aPC	0.9391
unstimulated vs. THR	0.9489
unstimulated vs. IL1	0.0725
unstimulated vs. aPC IL1	0.9391
aPC vs. THR	0.9391
aPC vs. IL1	0.0099
aPC vs. aPC IL1	0.6464
THR vs. IL1	0.0765
THR vs. aPC IL1	0.9391
IL1 vs. aPC IL1	0.282

Fig. 6D	
Tukey's multiple comparisons test	Adjusted P Value
IL-1 vs. THR	0.0032
IL-1 vs. aPC + IL-1	0.0218
IL-1 vs. aPC	0.0002
IL-1 vs. unstim	0.0062
THR vs. aPC + IL-1	0.8518
THR vs. aPC	0.5146
THR vs. unstim	0.9965
aPC + IL-1 vs. aPC	0.1189
aPC + IL-1 vs. unstim	0.9639
aPC vs. unstim	0.3317

Fig. 6E<br>Holm-Šídák's multiple comparisons test	Adjusted P Value
0	
$EPCR^+$ unstim vs. $EPCR^-$ unstim	0.6026
$EPCR^+$ unstim vs. $EPCR^+$ IL-1α	0.7639
$EPCR^+$ unstim vs. $EPCR^-$ IL-1α	0.3017
$EPCR^-$ unstim vs. $EPCR^+$ IL-1α	0.8315
$EPCR^-$ unstim vs. $EPCR^-$ IL-1α	0.8315
$EPCR^+$ IL-1α vs. $EPCR^-$ IL-1α	0.7639
1	
$EPCR^+$ unstim vs. $EPCR^-$ unstim	0.0102
$EPCR^+$ unstim vs. $EPCR^+$ IL-1α	0.0291
$EPCR^+$ unstim vs. $EPCR^-$ IL-1α	<0.0001
$EPCR^-$ unstim vs. $EPCR^+$ IL-1α	0.6461
$EPCR^-$ unstim vs. $EPCR^-$ IL-1α	0.1583
$EPCR^+$ IL-1α vs. $EPCR^-$ IL-1α	0.0876
2	
$EPCR^+$ unstim vs. $EPCR^-$ unstim	0.0017
$EPCR^+$ unstim vs. $EPCR^+$ IL-1α	0.0568
$EPCR^+$ unstim vs. $EPCR^-$ IL-1α	0.0017
$EPCR^-$ unstim vs. $EPCR^+$ IL-1α	0.4324
$EPCR^-$ unstim vs. $EPCR^-$ IL-1α	0.9769
$EPCR^+$ IL-1α vs. $EPCR^-$ IL-1α	0.4324
3+	
$EPCR^+$ unstim vs. $EPCR^-$ unstim	0.7334
$EPCR^+$ unstim vs. $EPCR^+$ IL-1α	0.6629
$EPCR^+$ unstim vs. $EPCR^-$ IL-1α	0.0276
$EPCR^-$ unstim vs. $EPCR^+$ IL-1α	0.7404
$EPCR^-$ unstim vs. $EPCR^-$ IL-1α	0.1332
$EPCR^+$ IL-1α vs. $EPCR^-$ IL-1α	0.2177

Fig. 6F<br>Holm-Šídák's multiple comparisons test	Adjusted P Value
$EPCR^-$:unstim vs. $EPCR^+$:unstim	0.0284
$EPCR^-$:IL-1α vs. $EPCR^+$:IL-1α	0.0118
$EPCR^-$:unstim vs. $EPCR^-$:IL-1α	0.0387
$EPCR^+$:unstim vs. $EPCR^+$:IL-1α	0.062

Appendix Table S8: P values reported in Figure 7

Fig. 7C %	
Dunn's multiple comparisons test	Adjusted P Value
control vs. aPC	0.36
control vs. THR	>0.99
control vs. PM2+THR	>0.99
aPC vs. THR	0.03
aPC vs. PM2+THR	>0.99
THR vs. PM2+THR	0.48

Fig.7C count	
Dunn's multiple comparisons test	Adjusted P Value
control vs. aPC	0.51
control vs. THR	>0.99
control vs. PM2+THR	>0.99
aPC vs. THR	0.04
aPC vs. PM2+THR	>0.99
THR vs. PM2+THR	0.33

Fig. 7E %	
Dunn's multiple comparisons test	Adjusted P Value
control vs. aPC	0.08
control vs. THR	>0.99
control vs. PM2+THR	>0.99
aPC vs. THR	0.003
aPC vs. PM2+THR	>0.99
THR vs. PM2+THR	0.1

Fig. 7E count	
Dunn's multiple comparisons test	Adjusted P Value
control vs. aPC	0.21
control vs. THR	>0.99
control vs. PM2+THR	0.92
aPC vs. THR	0.01
aPC vs. PM2+THR	>0.99
THR vs. PM2+THR	0.09

Fig. 7F %PAR1+	
Dunn's multiple comparisons test	Adjusted P Value
control vs. aPC	>0.99
control vs. THR	>0.99
control vs. PM2+THR	>0.99
aPC vs. THR	>0.99
aPC vs. PM2+THR	>0.99
THR vs. PM2+THR	>0.99

Fig. 7F %EPCR+	
Dunn's multiple comparisons test	Adjusted P Value
control vs. aPC	0.05
control vs. THR	>0.99
control vs. PM2+THR	>0.99
aPC vs. THR	0.03
aPC vs. PM2+THR	0.92
THR vs. PM2+THR	0.92

Fig. 7G %	
Dunn's multiple comparisons test	Adjusted P Value
control vs. aPC	0.06

control vs. THR	>0.99
control vs. PM2+THR	>0.99
aPC vs. THR	0.008
aPC vs. PM2+THR	0.85
THR vs. PM2+THR	0.5

Fig. 7G count	
Dunn's multiple comparisons test	Adjusted P Value
control vs. aPC	0.21
control vs. THR	>0.99
control vs. PM2+THR	>0.99
aPC vs. THR	0.05
aPC vs. PM2+THR	>0.99
THR vs. PM2+THR	0.97

Fig. 7I %	
Dunn's multiple comparisons test	Adjusted P Value
control vs. aPC	0.003
control vs. THR	>0.99
control vs. PM2+THR	0.56
aPC vs. THR	<0.001
aPC vs. PM2+THR	0.42
THR vs. PM2+THR	0.16

Fig. 7I count	
Dunn's multiple comparisons test	Adjusted P Value
control vs. aPC	0.003
control vs. THR	>0.99
control vs. PM2+THR	0.84
aPC vs. THR	<0.001
aPC vs. PM2+THR	0.29
THR vs. PM2+THR	0.18

Fig. 7J myeloid cells	
Dunn's multiple comparisons test	Adjusted P Value
control vs. aPC	0.01
control vs. THR	>0.99
control vs. PM2+THR	>0.99
aPC vs. THR	<0.001
aPC vs. PM2+THR	0.11
THR vs. PM2+THR	0.75

Fig. 7J B cells	
Dunn's multiple comparisons test	Adjusted P Value
control vs. aPC	0.02
control vs. THR	>0.99
control vs. PM2+THR	0.91
aPC vs. THR	0.003
aPC vs. PM2+THR	0.71
THR vs. PM2+THR	0.35

Fig. 7J T cells	
Dunn's multiple comparisons test	Adjusted P Value
control vs. aPC	0.006
control vs. THR	>0.99
control vs. PM2+THR	>0.99
aPC vs. THR	<0.001
aPC vs. PM2+THR	0.05
THR vs. PM2+THR	>0.99

Appendix Table S9: P values reported in Figure EV1

Fig. EV1B	
Holm-Šídák's multiple comparisons test	Adjusted P Value
CD34-	
0 vs. 2	0.067
0 vs. 5	0.0044
0 vs. 7	0.0222
CD34+	
0 vs. 2	0.0001
0 vs. 5	0.1173
0 vs. 7	0.0018
CD34+CD45RA-CD90+	
0 vs. 2	0.9757
0 vs. 5	0.9757
0 vs. 7	0.9757
CD34+CD45RA-CD90-	
0 vs. 2	0.1378
0 vs. 5	0.0032
0 vs. 7	0.1481

Fig. EV1C	
Holm-Šídák's multiple comparisons test	Adjusted P Value
CD34+	
0 vs. 2	0.0146
0 vs. 5	0.0078
0 vs. 7	0.0146
CD34+CD45RA-CD90+	
0 vs. 2	<0.0001
0 vs. 5	<0.0001
0 vs. 7	<0.0001
CD34+CD45RA-CD90-	
0 vs. 2	0.3354
0 vs. 5	0.2639
0 vs. 7	0.424
CD34-	
0 vs. 2	0.0009
0 vs. 5	0.3548
0 vs. 7	0.1083

Fig. EC1D	
Tukey's multiple comparisons test	Adjusted P Value
$CD34^-$ vs. $CD34^+$	0.041
$CD34^-$ vs. $CD90^-$	0.0997
$CD34^-$ vs. $CD90^+$	<0.0001
$CD34^-$ vs. $EPCR^-$	0.0001
$CD34^-$ vs. $EPCR^+$	<0.0001
$CD34^+$ vs. $CD90^-$	0.9934
$CD34^+$ vs. $CD90^+$	0.0065
$CD34^+$ vs. $EPCR^-$	0.0265
$CD34^+$ vs. $EPCR^+$	<0.0001

CD90$^-$ vs. CD90$^+$	0.0027
CD90$^-$ vs. EPCR$^-$	0.0107
CD90$^-$ vs. EPCR$^+$	<0.0001
CD90$^+$ vs. EPCR$^-$	0.9544
CD90$^+$ vs. EPCR$^+$	0.1246
EPCR$^-$ vs. EPCR$^+$	0.0312

Fig. EV1E	
Holm-Šídák's multiple comparisons test	Adjusted P Value
cell surface:unstim vs. cell surface:THR	0.0467
intracellular:unstim vs. intracellular:THR	0.2654
cell surface:unstim vs. intracellular:unstim	0.011
cell surface:THR vs. intracellular:THR	0.0012

Fig. EV1F RCR-401	
Unpaired t test	P value
aPC vs. unstim	0.0032

Fig. EV1F RCR-252	
Unpaired t test	P value
aPC vs. unstim	0.0041

Fig. EV1F REA337	
Unpaired t test	P value
aPC vs. unstim	0.0071

Fig. EV1G	
Tukey's multiple comparisons test	Adjusted P Value
unstim vs. aPC	0.0009
unstim vs. EPCR-Ab+aPC	0.0005
aPC vs. EPCR-Ab+aPC	0.903

Appendix Table S10: P values reported in Figure EV2

Fig. EV2C bar chart	
Tukey's multiple comparisons test	Adjusted P Value
THR vs. control	0.0039
THR vs. aPC	0.0128
control vs. aPC	0.5097

Fig. EV2D	
Tukey's multiple comparisons test	Adjusted P Value
control vs. 5 min	0.9953
control vs. 10 min	0.9553
5 min vs. 10 min	0.9789

Fig. EV2E	
Tukey's multiple comparisons test	Adjusted P Value
control vs. 5 min	0.9949
control vs. 10 min	0.9766
5 min vs. 10 min	0.9932

Fig. EV2G Bar chart	
Unpaired t test	P value
EPCR+ vs. EPCR-	0.7345

Fig. EV2I	
Unpaired t test	P value
CD90- vs CD90+	0.0065

Fig. EV2J	
Unpaired t test	P value
EPCR- vs. EPCR+	0.0245

Appendix Table S11: P values reported in Figure EV3

Fig. EV3A CREB	
Dunnett's multiple comparisons test	Adjusted P Value
0 vs. 10	0.0103
0 vs. 30	0.8076
0 vs. 60	0.9966
0 vs. 120	0.6347

Fig. EV3A AKT	
Dunnett's multiple comparisons test	Adjusted P Value
0 vs. 10	0.0906
0 vs. 30	0.8923
0 vs. 60	0.9971
0 vs. 120	0.7812

Fig. EV3A AKT	
Dunnett's multiple comparisons test	Adjusted P Value
0 vs. 10	0.531
0 vs. 30	0.9472
0 vs. 60	0.4397
0 vs. 120	0.2099

Fig. EV3A NfKB	
Dunnett's multiple comparisons test	Adjusted P Value
0 vs. 10	0.8162
0 vs. 30	0.9998
0 vs. 60	>0.9999
0 vs. 120	0.8712

Fig. EV3C	
Holm-Šídák's multiple comparisons test	Adjusted P Value
0	
bulk + aPC vs. EPCR AB + aPC	>0.9999
bulk + aPC vs. sorted EPCR-	>0.9999
10	
bulk + aPC vs. EPCR AB + aPC	0.0074
bulk + aPC vs. sorted EPCR-	0.0185
30	
bulk + aPC vs. EPCR AB + aPC	0.9992
bulk + aPC vs. sorted EPCR-	>0.9999
60	
bulk + aPC vs. EPCR AB + aPC	>0.9999
bulk + aPC vs. sorted EPCR-	>0.9999
120	
bulk + aPC vs. EPCR AB + aPC	0.9998
bulk + aPC vs. sorted EPCR-	>0.9999

Fig. EV3D	
Holm-Šídák's multiple comparisons test	Adjusted P Value
0	
bulk + aPC vs. EPCR AB + aPC	>0.9999
bulk + aPC vs. EPCR- sorted + aPC	>0.9999

10	
bulk + aPC vs. EPCR AB + aPC	0.8907
bulk + aPC vs. EPCR- sorted + aPC	0.0934
30	
bulk + aPC vs. EPCR AB + aPC	0.8068
bulk + aPC vs. EPCR- sorted + aPC	>0.9999
60	
bulk + aPC vs. EPCR AB + aPC	>0.9999
bulk + aPC vs. EPCR- sorted + aPC	0.4985
120	
bulk + aPC vs. EPCR AB + aPC	>0.9999
bulk + aPC vs. EPCR- sorted + aPC	0.5952

Appendix Table S12: P values reported in Figure EV5

Fig. EV5B OCR	
Tukey's multiple comparisons test	Adjusted P Value
control vs. PM2	0.8442
control vs. THR	0.5194
control vs. PM2 + THR	0.9773
control vs. aPC	0.9955
PM2 vs. THR	0.9787
PM2 vs. PM2 + THR	0.5136
PM2 vs. aPC	0.637
THR vs. PM2 + THR	0.2263
THR vs. aPC	0.3132
PM2 + THR vs. aPC	0.9996

Fig. EV5B ECAR	
Tukey's multiple comparisons test	Adjusted P Value
control vs. PM2	>0.9999
control vs. THR	0.0011
control vs. PM2 + THR	0.3476
control vs. aPC	0.9988
PM2 vs. THR	0.0013
PM2 vs. PM2 + THR	0.3947
PM2 vs. aPC	0.9963
THR vs. PM2 + THR	0.0921
THR vs. aPC	0.0005
PM2 + THR vs. aPC	0.2276

Fig. EV5C ATP-production coupled resp.	
Tukey's multiple comparisons test	Adjusted P Value
control vs. PM2	0.9194
control vs. THR	0.5393
control vs. PM2 + THR	0.5351
control vs. aPC	0.8428
PM2 vs. THR	0.9485
PM2 vs. PM2 + THR	0.9468
PM2 vs. aPC	0.9997
THR vs. PM2 + THR	>0.9999
THR vs. aPC	0.9832
PM2 + THR vs. aPC	0.9824

Fig. EV5C Coupling eff.	
Tukey's multiple comparisons test	Adjusted P Value
control vs. PM2	0.9987
control vs. THR	0.574
control vs. PM2 + THR	0.8107
control vs. aPC	0.9975
PM2 vs. THR	0.7367
PM2 vs. PM2 + THR	0.6582
PM2 vs. aPC	0.9748
THR vs. PM2 + THR	0.1039
THR vs. aPC	0.3853
PM2 + THR vs. aPC	0.938

Fig. EV5C Proton leak	
Tukey's multiple comparisons test	Adjusted P Value
control vs. PM2	0.9951
control vs. THR	>0.9999
control vs. PM2 + THR	0.3762

control vs. aPC	0.8245
PM2 vs. THR	0.9929
PM2 vs. PM2 + THR	0.6006
PM2 vs. aPC	0.9595
THR vs. PM2 + THR	0.3556
THR vs. aPC	0.8049
PM2 + THR vs. aPC	0.9352

Appendix Table S13: P values reported in Figure EV6

Fig. EV6B	
Tukey's multiple comparisons test	Adjusted P Value
DMSO vs. aPC	0.9196
DMSO vs. THR	0.9587
DMSO vs. PM2+THR	0.7543
aPC vs. THR	0.6664
aPC vs. PM2+THR	0.9855
THR vs. PM2+THR	0.4419

Fig. EV6C	
Tukey's multiple comparisons test	Adjusted P Value
DMSO vs. aPC	<0.0001
DMSO vs. THR	0.0049
DMSO vs. PM2+THR	0.9955
aPC vs. THR	<0.0001
aPC vs. PM2+THR	<0.0001
THR vs. PM2+THR	0.0101

Fig EV6D 1st plating CFU-GM	
Tukey's multiple comparisons test	Adjusted P Value
unstimulated vs. aPC 10µg/ml	0.9272
unstimulated vs. THR	0.9704
aPC 10µg/ml vs. THR	0.9895

Fig. EV6G 1st plating CFU-E	
Tukey's multiple comparisons test	Adjusted P Value
unstimulated vs. aPC 10µg/ml	0.9629
unstimulated vs. THR	0.9917
aPC 10µg/ml vs. THR	0.9893

Fig. EV6G 1st plating CFU-GEMM	
Tukey's multiple comparisons test	Adjusted P Value
unstimulated vs. aPC 10µg/ml	0.1751
unstimulated vs. THR	0.1769
aPC 10µg/ml vs. THR	>0.9999

Fig. EV6D 2nd plating CFU-GM	
Tukey's multiple comparisons test	Adjusted P Value
unstimulated vs. aPC 10µg/ml	0.656
unstimulated vs. THR	0.8364
aPC 10µg/ml vs. THR	0.9426

Fig. EV6D 2nd plating CFU-E	
Tukey's multiple comparisons test	Adjusted P Value
unstimulated vs. aPC 10µg/ml	0.6949
unstimulated vs. THR	0.6466
aPC 10µg/ml vs. THR	0.996

Fig. EV6E	
Tukey's multiple comparisons test	Adjusted P Value
THR vs. aPC	0.1397
THR vs. unstim	0.8921
aPC vs. unstim	0.288

Fig. EV6F unstim, aPC, THR	
Tukey's multiple comparisons test	Adjusted P Value

unstim vs. aPC	0.9986
unstim vs. THR	0.9754
aPC vs. THR	0.9628

Fig. EV6F DMSO, TG	
Unpaired t test	P value
DMSO vs. TG	<0.0001

Fig. EV6G unstim, aPC, THR	
Tukey's multiple comparisons test	Adjusted P Value
unstim vs. aPC	0.9871
unstim vs. THR	0.9919
aPC vs. THR	0.9994

Fig. EV6G DMSO, TG	
Unpaired t test	P value
DMSO vs. TG	0.0009

Fig. EV6H	
Unpaired t test	P value
DMSO vs. TG	0.0001

Fig. EV6I	
Unpaired t test	P value
DMSO vs. TG	0.0012

Fig. EV6J	
Tukey's multiple comparisons test	Adjusted P Value
IL1 vs. THR	0.9344
IL1 vs. aPC + IL1	0.9826
IL1 vs. aPC	0.0929
IL1 vs. unstim	0.7984
THR vs. aPC + IL1	0.9992
THR vs. aPC	0.3472
THR vs. unstim	0.997
aPC + IL1 vs. aPC	0.2401
aPC + IL1 vs. unstim	0.9769
aPC vs. unstim	0.5328

Fig. EV6K	
Tukey's multiple comparisons test	Adjusted P Value
IL-1 vs. THR	0.0018
IL-1 vs. aPC + IL-1	0.0174
IL-1 vs. aPC	<0.0001
IL-1 vs. unstim	0.008
THR vs. aPC + IL-1	0.7711
THR vs. aPC	0.4808
THR vs. unstim	0.9378
aPC + IL-1 vs. aPC	0.0801
aPC + IL-1 vs. unstim	0.9939
aPC vs. unstim	0.1609

Fig. EV6L	
Holm-Šídák's multiple comparisons test	Adjusted P Value
$EPCR^+$ vs. $EPCR^-$	0.0744
$EPCR^+$ vs. $EPCR^-$	0.0744
$EPCR^+$ vs. $EPCR^+$	0.0921
$EPCR^-$ vs. $EPCR^-$	0.0921

Appendix Table S14: P values reported in Figure EV7

Fig. EV7A %	
Dunn's multiple comparisons test	Adjusted P Value
control vs. aPC	0.03
control vs. THR	>0.99
control vs. PM2+THR	>0.99
aPC vs. THR	<0.001
aPC vs. PM2+THR	0.2
THR vs. PM2+THR	0.38

Fig. EV7A count/ul	
Dunn's multiple comparisons test	Adjusted P Value
control vs. aPC	0.009
control vs. THR	>0.99
control vs. PM2+THR	0.63
aPC vs. THR	<0.001
aPC vs. PM2+THR	0.71
THR vs. PM2+THR	0.08

Fig. EV7B %	
Dunn's multiple comparisons test	Adjusted P Value
control vs. aPC	0.01
control vs. THR	>0.99
control vs. PM2+THR	>0.99
aPC vs. THR	<0.001
aPC vs. PM2+THR	0.36
THR vs. PM2+THR	0.3

Fig. EV7B count/ul	
Dunn's multiple comparisons test	Adjusted P Value
control vs. aPC	0.27
control vs. THR	>0.99
control vs. PM2+THR	>0.99
aPC vs. THR	0.008
aPC vs. PM2+THR	>0.99
THR vs. PM2+THR	0.1

Fig. EV7C myeloid cells	
Dunn's multiple comparisons test	Adjusted P Value
control vs. aPC	>0.99
control vs. THR	>0.99
control vs. PM2+THR	>0.99
aPC vs. THR	0.19
aPC vs. PM2+THR	>0.99
THR vs. PM2+THR	>0.99

Fig. EV7C B cells	
Dunn's multiple comparisons test	Adjusted P Value
control vs. aPC	0.86
control vs. THR	>0.99
control vs. PM2+THR	>0.99
aPC vs. THR	0.07
aPC vs. PM2+THR	>0.99
THR vs. PM2+THR	0.67

Fig. EV7C T cells	
Dunn's multiple comparisons test	Adjusted P Value
control vs. aPC	0.03

control vs. THR	>0.99
control vs. PM2+THR	>0.99
aPC vs. THR	<0.001
aPC vs. PM2+THR	0.21
THR vs. PM2+THR	0.41

Fig. EV7D

Holm-Šídák's multiple comparisons test	Adjusted P Value
LYM (B + T)	
control vs. aPC	0.008
control vs. THR	0.36
control vs. PM2+THR	0.3
aPC vs. THR	0.2
aPC vs. PM2+THR	0.3
THR vs. PM2+THR	0.64
MYE	
control vs. aPC	0.99
control vs. THR	0.52
control vs. PM2+THR	0.99
aPC vs. THR	0.58
aPC vs. PM2+THR	0.99
THR vs. PM2+THR	0.52
CD34+	
control vs. aPC	0.01
control vs. THR	0.74
control vs. PM2+THR	0.21
aPC vs. THR	0.005
aPC vs. PM2+THR	0.33
THR vs. PM2+THR	0.14

Fig. EV7E

Holm-Šídák's multiple comparisons test	Adjusted P Value
LYM (B + T)	
control vs. aPC	<0.001
control vs. THR	0.18
control vs. PM2+THR	0.18
aPC vs. THR	<0.001
aPC vs. PM2+THR	0.004
THR vs. PM2+THR	0.008
MYE	
control vs. aPC	0.96
control vs. THR	0.97
control vs. PM2+THR	0.97
aPC vs. THR	0.88
aPC vs. PM2+THR	0.96
THR vs. PM2+THR	0.97
CD34+	
control vs. aPC	<0.001
control vs. THR	0.25
control vs. PM2+THR	0.2
aPC vs. THR	<0.001
aPC vs. PM2+THR	0.03
THR vs. PM2+THR	0.03

Flow Cytometry Panels

Flow Cytometry Panel (1) – PAR1 expression on human CD34+ cells

Antibody	***Conjugate***	***Concentration***	***Source / Cat#***
Anti-human CD34	Vioblue	1:100	Miltenyi #130-113-182
Anti-human CD38	BV605	1:100	BD bioscience #562665
Anti-human CD45RA	BV786	1:100	BD bioscience. #563870
Anti-human CD90	AF700	1:100	BioLegend #328120
Anti-human EPCR	APC	1:50	Biolegend, #351906
Anti-human PAR1	PE	1:50	BeckmanCoulter #IM2584
Dead/Alive	7-AAD	1:100	Biolegend #420404

Flow Cytometry Panel (2) – Single cell sort and expansion analysis

Antibody	***Conjugate***	***Concentration***	***Source / Cat#***
Anti-human CD34	APC-Vio700	1:100	Miltenyi #130-124-457
Anti-human CD90	BV785	1:100	BioLegend #328142
Dead/Alive	PI	1:10000	MP Biologicals #02195458-

Flow Cytometry Panel (3) – Peripheral whole blood

Antibody	***Conjugate***	***Concentration***	***Source / Cat#***
Anti-mouse CD45	SparkUV 387	1:200	BioLegend #103188
Anti-mouse Ter119	PE-Cy5	1:200	BioLegend #116210
Anti-mouse CD61	Alexa Fluor 647	1:100	BioLegend #133934
Anti-human CD45	PE Dazzle 594	1:100	BioLegend #304052
Anti-human CD41	BV421	1:100	BioLegend #303730

Flow Cytometry Panel (4) – RBC lysed blood

Antibody	***Conjugate***	***Concentration***	***Source / Cat#***
Anti-mouse CD45	SparkUV 387	1:200	BioLegend #103188
Anti-mouse Ter119	PE-Cy5	1:200	BioLegend #116210
Anti-human CD45	APC-Cy7	1:100	BioLegend #304014
Dead/Alive	PI	1:10000	MP Bio #02195458-CF

Flow Cytometry Panel (5) – Bone marrow cells

Antibody	***Conjugate***	***Concentration***	***Source / Cat#***
Anti-mouse CD45	SparkUV 387	1:200	BioLegend #103188
Anti-mouse Ter119	PE-Cy5	1:200	BioLegend #116210
Anti-human CD19	FITC	1:100	BioLegend #392507
Anti-human CD20	FITC	1:100	BioLegend #302303
Anti-human CD33	APC	1:100	BioLegend #303407
Anti-human CD34	PE-Cy7	1:100	BD Bioscience #560710
Anti-human CD45	APC-Cy7	1:100	BioLegend #304014
Anti-human CD3	BV510	1:100	BioLegend #317332
Anti-human CD38	BV605	1:100	BioLegend #356641

scRNAseq Panel

Antibody	***Conjugate***	***Concentration***	***Source / Cat#***
Anti-human CD38	oligonucleotide	1:50	BD Bioscience #940466
Anti-human CD45RA	oligonucleotide	1:50	BD Bioscience # 940011
Anti-human CD44	oligonucleotide	1:50	BD Bioscience # 940039
Anti-human CD10	oligonucleotide	1:50	BD Bioscience # 940045
Anti-human CD123	oligonucleotide	1:50	BD Bioscience # 940020
Anti-human CD127	oligonucleotide	1:50	BD Bioscience # 940012
Anti-human CD41a	oligonucleotide	1:50	BD Bioscience # 940219
Anti-human CD110	oligonucleotide	1:50	BD Bioscience # 940302
Anti-human CD49f	oligonucleotide	1:50	BD Bioscience # 940160
Anti-human CD90	oligonucleotide	1:50	BD Bioscience # 940032
Anti-human CD34	oligonucleotide	1:50	BD Bioscience # 940021
