# Supplementary material for: Activated protein C promotes hematopoietic stem and progenitor cell quiescence and engraftment
Source: EMBO Mol Med. 2026 Oct 9;18(11):4433–67. doi: 10.1038/s44321-026-00529-y (PMC13748566; doi:10.1038/s44321-026-00529-y)
Supplement: Supplementary file 2 [file 44321_2026_529_MOESM2_ESM.pdf]

EMBO Molecular Medicine

Activated Protein C promotes Hematopoietic Stem and Progenitor Cell Quiescence and Engraftment

Simon Pöllmann, M Rommel, Leal Oburoglu, Tanja Hirch, Fabiola Valdivia- Francia, Kah Mun Siow, Timon Menzi, Julia Ackva, Franziska Schenk, Matthias Neeracher, Ataman Sendoel, Janine Reichenbach, Camilla Forsberg, and Ute Modlich

Corresponding author: Ute Modlich (ute.modlich@irem.uzh.ch)

Review Timeline:

Submission Date:	10th Feb 26
Editorial Decision:	13th Mar 26
Revision Received:	30th Jun 26
Editorial Decision:	22nd Jul 26
Revision Received:	19th Aug 26
Accepted:	14th Sep 26

Editor: Lise Roth

Transaction Report:

(Note: With the exception of the correction of typographical or spelling errors that could be a source of ambiguity, letters and reports are not edited. Depending on transfer agreements, referee reports obtained elsewhere may or may not be included in this compilation. Referee reports are anonymous unless the Referee chooses to sign their reports.)

13th Mar 2026

Dear Prof. Modlich,

Thank you for submitting your manuscript to EMBO Molecular Medicine, and please accept my apologies for the delay in getting back to you as I was waiting for a referee report. Unfortunately, referee #1 has not yet gotten back to us, but as both referees #2 and #3 make similar recommendations, we would like to make a decision now to avoid further delay to the process. If referee #1 provides a report, we will send it to you on the understanding that we will not ask you to carry out any extensive experiments beyond those requested in the attached reports. As you will see below, the referees recognize the interest of the study and generally support the publication of your work, provided appropriate revisions.

Addressing the reviewers' concerns in full will be necessary for further considering the manuscript in our journal, and acceptance of the manuscript will entail a second round of review. EMBO Molecular Medicine encourages a single round of revision only and therefore, acceptance or rejection of the manuscript will depend on the completeness of your responses included in the next, final version of the manuscript. For this reason, and to save you frustration at the end, I would strongly discourage you from returning an incomplete revision.

If you would like to discuss further the points raised by the referees, I am available to do so via email or video. Let me know if you are interested in this option.

We are expecting your revised manuscript within three to four months, if you anticipate any delay, please contact us.

When preparing your revised manuscript, please refer to our guidelines: https://link.springer.com/journal/44321/submission-guidelines#cms-Revised-submissions. We perform an initial quality control of all revised manuscripts before re-review; failure to include requested items will delay the evaluation of your revision.

We require:

1) A .docx formatted version of the manuscript text (including legends for main figures, EV figures and tables). Please make sure that the changes are highlighted to be clearly visible.

2) Individual production quality figure files as .eps, .tif, .jpg (one file per figure). For guidance, download the 'Figure Guide PDF': https://media.springernature.com/original/springer-cms/rest/v1/content/27825798/data/v1.

3) A .docx formatted letter INCLUDING the reviewers' reports and your detailed point-by-point responses to their comments. As part of the EMBO Press transparent editorial process, the point-by-point response is part of the Review Process File (RPF), which will be published alongside your paper.

4) A complete author checklist, which you can download from our author guidelines. Please insert information in the checklist that is also reflected in the manuscript. The completed author checklist will also be part of the RPF.

5) Please note that all corresponding authors are required to supply an ORCID ID for their name upon submission of a revised manuscript.

6) It is mandatory to include a 'Data Availability' section after the Materials and Methods. Before submitting your revision, primary datasets produced in this study need to be deposited in an appropriate public database, and the accession numbers and database listed under 'Data Availability'. Please remember to provide a reviewer password if the datasets are not yet public.

In case you have no data that requires deposition in a public database, please state so in this section. Note that the Data Availability Section is restricted to new primary data that are part of this study.

7) For data quantification: please specify the name of the statistical test used to generate error bars and P values, the number (n) of independent experiments (specify technical or biological replicates) underlying each data point and the test used to calculate p-values in each figure legend. The figure legends should contain a basic description of n, P and the test applied. Graphs must include a description of the bars and the error bars (s.d., s.e.m.).

8) At EMBO Press we ask authors to provide source data for the main manuscript figures. You will receive a separate email with instructions for providing source data with your revised manuscript, including how to upload and organize the files.

9) Our journal encourages inclusion of *data citations in the reference list* to directly cite datasets that were re-used and obtained from public databases. Data citations in the article text are distinct from normal bibliographical citations and should

directly link to the database records from which the data can be accessed. In the main text, data citations are formatted as follows: "Data ref: Smith et al, 2001" or "Data ref: NCBI Sequence Read Archive PRJNA342805, 2017". In the Reference list, data citations must be labeled with "[DATASET]". A data reference must provide the database name, accession number/identifiers and a resolvable link to the landing page from which the data can be accessed at the end of the reference.

10) We replaced Supplementary Information with Expanded View (EV) Figures and Tables that are collapsible/expandable online. A maximum of 5 EV Figures can be typeset. EV Figures should be cited as 'Figure EV1, Figure EV2" etc... in the text and their respective legends should be included in the main text after the legends of regular figures.

- For the figures that you do NOT wish to display as Expanded View figures, they should be bundled together with their legends in a single PDF file called *Appendix*, which should start with a short Table of Content. Appendix figures should be referred to in the main text as: "Appendix Figure S1, Appendix Figure S2" etc.

- Additional Tables/Datasets should be labeled and referred to as Table EV1, Dataset EV1, etc. Legends have to be provided in a separate tab in case of .xls files. Alternatively, the legend can be supplied as a separate text file (README) and zipped together with the Table/Dataset file.

11) The paper explained: EMBO Molecular Medicine articles are accompanied by a summary of the articles to emphasize the major findings in the paper and their medical implications for the non-specialist reader. Please provide a draft summary of your article highlighting
- the medical issue you are addressing,
- the results obtained and
- their clinical impact.

This may be edited to ensure that readers understand the significance and context of the research. Please refer to any of our published articles for an example.

12) Author contributions: You will be asked to provide CRediT (Contributor Role Taxonomy) terms in the submission system. These replace a narrative author contribution section in the manuscript.

13) A Conflict of Interest statement should be provided in the main text.

14) Every published paper includes a 'Synopsis' to further enhance discoverability. Synopses are displayed on the journal webpage and are freely accessible to all readers. They include a short stand first (maximum of 300 characters, including space) as well as 2-5 one-sentences bullet points that summarizes the paper. Please write the bullet points to summarize the key NEW findings. They should be designed to be complementary to the abstract - i.e. not repeat the same text. We encourage inclusion of key acronyms and quantitative information (maximum of 30 words / bullet point). Please use the passive voice. Please attach these in a separate file or send them by email, we will incorporate them accordingly.

Please also suggest a striking image or visual abstract to illustrate your article as a PNG file 550 px wide x 300-600 px high.

15) Include a Reagents and Tools Table as part of the Methods section, which can be downloaded from our author guidelines.

16) As part of the EMBO Publications transparent editorial process initiative (see our Editorial at http://embomolmed.embopress.org/content/2/9/329), EMBO Molecular Medicine will publish online a Review Process File (RPF) to accompany accepted manuscripts.
In the event of acceptance, this file will be published in conjunction with your paper and will include the anonymous referee reports, your point-by-point response and all pertinent correspondence relating to the manuscript. Let us know whether you agree with the publication of the RPF and as here, if you want to remove or not any figures from it prior to publication.
Please note that the Authors checklist will be published at the end of the RPF.

EMBO Molecular Medicine has a "scooping protection" policy, whereby similar findings that are published by others during review or revision are not a criterion for rejection. Should you decide to submit a revised version, I do ask that you get in touch after three months if you have not completed it, to update us on the status.

I look forward to receiving your revised manuscript.

Yours sincerely,

Lise Roth

Lise Roth, PhD

Senior Editor
EMBO Molecular Medicine

***** Reviewer's comments *****

Referee #2 (Comments on Novelty/Model System for Author):

They demonstrate that EPCR/PAR1 on human hematopoietic stem and progenitor (HSPC) can be activated by both thrombin (THR) and activated protein C (aPC), inducing distinct signaling.
They should discuss more about the in vivo function of the both signaling in the hematopoiesis.

Referee #2 (Remarks for Author):

The manuscript submitted by Pöllmann et al. investigated the role of EPCR/PAR1 signaling in human hematopoietic stem cells (HSCs), with a focus on how different activators of PAR1-activated protein C (aPC) and thrombin (THR)-exert opposing effects on HSC function. The authors demonstrated that aPC and THR, while acting through the same receptor PAR1, activate distinct signaling pathways: aPC preferentially activates AKT and CREB, conferring cytoprotective effects, whereas THR induces $Ca2+$ flux, ERK1/2 phosphorylation, and glycolytic activation. Using a combination of flow cytometry, single-cell RNA sequencing, metabolic analysis, and in vivo transplantation assays, the authors showed that aPC treatment preserves HSC quiescence, enhances clonogenic potential, and significantly improves engraftment in primary and secondary NBSGW mouse recipients. In contrast, THR promotes HSC differentiation, metabolic stress, and compromises transplant outcomes. Mechanistically, aPC downregulates DNA replication-associated genes and counteracts IL-1α-induced proliferation, while THR drives myeloid differentiation genes and accumulates cytotoxic MGO. The use of the Parmodulin 2 (PM2) further confirmed that blocking THR-mediated signaling can rescue HSC function. Overall, this study provides evidence that biased PAR1 signaling is a key regulator of human HSC fate and identifies aPC treatment as a simple and potential translational strategy to enhance HSC engraftment in clinical transplantation. However, several concerns should be addressed to further strengthen the manuscript:

Major concerns

1. The authors suggest in the Discussion that aPC may act independently of EPCR due to the high-dose approach used in this study. However, direct experimental evidence supporting this conclusion is lacking. Could the authors compare signaling pathway activation (e.g., pAKT, pCREB) between EPCR+ and EPCR- HSCs following aPC treatment? That would help clarify whether EPCR is necessary for aPC-mediated protection.

2. The authors used PM2 to bias THR/PAR1 signaling toward an aPC-like cytoprotective profile. While PM2 effectively blocked THR-induced calcium flux, ERK activation, and engraftment defects, the data showed that PM2+THR treatment did not activate AKT or CREB as strongly as aPC. Could the authors clarify whether PM2 truly emulates aPC signaling or primarily acts as an antagonist of THR-mediated detrimental signaling?

3. The primary transplantation endpoint was 4 weeks post-transplant, which primarily reflects short-term repopulating cells rather than long-term HSCs. Although secondary transplantation data provide evidence for long-term repopulating capacity, could the authors extend the primary observation period to 16 weeks to confirm that aPC truly protects long-term HSCs? If chimerism in aPC-treated groups remains stable or increases over time, this would provide compelling evidence for sustained HSC function.

4. The authors showed that EPCR+ HSCs divide more slowly than EPCR- HSCs even under IL-1α stimulation. Could the authors briefly discuss this resistance in Discussion?

5.Parts of the Discussion restate results rather than interpreting them. Could the authors streamline the Discussion, focusing on study limitations, comparison with murine studies and future directions.
For example, how aPC and thrombin is functioning in vivo steady state hematopoiesis or stress hematopoiesis such as bleeding.

Minor concerns
The manuscript uses "HSC" and "HSPC" interchangeably. Could the authors clearly define in the Introduction when each term is appropriate?

Referee #3 (Remarks for Author):

In their manuscript entitled "Activated Protein C-PAR1 signaling promotes Hematopoietic Stem Cell Quiescence and Engraftment", Pöllmann et al. conduct a methodical analysis of aPC/PAR1 signalling in primary hematopoietic stem cells. Building on many studies which identify EPCR as a key molecule expressed on HSCs (especially in culture) and relatively limited data that goes after the mechanism of the pathway, this study represents a novel take on EPCR/aPC/PAR1 biology that should have strong relevance for the community.

The strength of the study is in its careful ste-by-step description of each aspect - first determining expression levels and rates of molecule recycling, second expanding the suite of assays (compared to published studies) exploring the nature of functional effects caused by the aPC/PAR1 axis, and third by careful in vitro and in vivo functional studies. Overall I think the manuscript is really nicely performed and backed by strong scientific thinking.

I have some minor considerations for the authors which I would hope could strengthen the manuscript if they are not too difficult to incorporate:

1) Orthogonal assays to measure calcium flux - the Ca2+ field has many assays and contradicting results. Have the authors tried any other assays (e.g., Fura molecules, Rhod2, etc)? If so, do they (broadly speaking) show a similar trend as Fig2a? Alternatively, have they considered patch-clamping for measuring rates of change in combination with the dyes? (please note that I wouldn't consider any of this essential to believe the finding, but if it were available or relatively easy to generate, I think it would add strength to the manuscript).

2) Behaviour in mutated cells - several mutations asssociated with clonal hematopoiesis or leukemia have been indicated to have changes in Ca2+ regulation (in particular TET2-mutated cells). If there is the ability to show that these cells behave differently in HSC-expansion settings, it would give a mechanistic reason for why TET2 mutated cells expand better than WT. (again, I would consider this type of "extra" beyond the scope of the current manuscript, but if it were available, I think it would expand the readership and interest for the manuscript).

3) With the conslusion on page 6 about quiescent v cycling HSCs, could the authors potentially assess fetal liver HSCs (actively cycling, EPCR+) in mouse to see how thapsigargin hits them? Maybe it would drive them into quiescence?

4) The authors are very careful with their language (which is great) regarding the conclusion: "aPC promoted clonogenic potential and maintained undifferentiated HSPCs in vitro." That said, the HSC field will be interested in whether short term treatment leads to expanded functional HSCs and secondary colony assays do not demonstrate this (although they indicate that it would be possible). Ideally a transplantation accompanies this experiment. Where they do conduct a transplantation, it is only after a very brief exposure and culture of human HSPCs (1h) and a very brief transplantation window (4 weeks primary, 4 weeks secondary). I would therefore suggest that the terminology and language around the experiments and conclusions on page 10 are tempered. The data show nicely that there is an effect on the stem/progenitor pool, but the assay does not demonstrate "the fundamental role of PAR1 signaling in HSC" or show that "a single dose of aPC maintained HSC activity even after serial transplantation."

Point-by-Point Response

Referee #2 (Comments on Novelty/Model System for Author):

They demonstrate that EPCR/PAR1 on human hematopoietic stem and progenitor (HSPC) can be activated by both thrombin (THR) and activated protein C (aPC), inducing distinct signaling.

They should discuss more about the in vivo function of the both signaling in the hematopoiesis.

Answer: We thank the reviewer for the constructive and positive feedback on our manuscript. In our revised manuscript, we address potential *in vivo* signaling of aPC and THR on HSCs in more detail, see also Answer 5.

Major concerns

Questions 1) The authors suggest in the Discussion that aPC may act independently of EPCR due to the high-dose approach used in this study. However, direct experimental evidence supporting this conclusion is lacking. Could the authors compare signaling pathway activation (e.g., pAKT, pCREB) between EPCR+ and EPCR- HSCs following aPC treatment? That would help clarify whether EPCR is necessary for aPC-mediated protection.

Answer 1) We conducted experiments to investigate whether EPCR expression was necessary to lead to PAR1 activation and whether there were differences in the signaling if EPCR was present on the cell surface. aPC stimulation led to reduced detection of EPCR on CD34+ cells when using two independent antibody clones (Fig. EV1F), due to competition of the antibody and aPC for the same binding sites in EPCR. This created a major technical hurdle to conduct the simultaneous detection of EPCR expression and signaling in phosphoflow analysis. We therefore used the EPCR-antibody to block aPC binding to EPCR (Fig EV1F) to answer the question whether EPCR was crucial for signalling pathway activation. The application of the anti-EPCR antibody did not change PAR1 activation after aPC stimulation as measured by PAR1 internalisation (Fig. EV1G). In addition, we conducted phosphoflow experiments using sorted EPCR-negative CD34+ cells and EPCR-blocked CD34+ cells (Fig. EV2G, H). In these experiments, we show that aPC-induced AKT phosphorylation was the same in the absence of EPCR or in case EPCR was blocked for aPC binding. However, there was a significantly reduced peak phosphoCREB signal comparing the not sorted, not blocked sample to either the blocked or the EPCR negative sorted samples after activation with aPC.

The new data are included in the results section on page 5 and in the Figures EV1F and EV2G/H.

Question 2) The authors used PM2 to bias THR/PAR1 signaling toward an aPC-like cytoprotective profile. While PM2 effectively blocked THR-induced calcium flux, ERK activation, and engraftment defects, the data showed that PM2+THR treatment did not activate AKT or CREB as strongly as aPC. Could the authors clarify whether PM2 truly emulates aPC signaling or primarily acts as an antagonist of THR-mediated detrimental signaling?

Answer 2) To clarify whether PM2 can act on PAR1 independently or requires the presence of THR, we conducted phosphoflow experiments in CD34+ cells after application of PM2 without THR. We observed CREB activation after 10 minutes, and a trend towards AKT activation, although this was not significant (Fig. EV2F). Our data are in contrast to a published study, showing significant AKT activation in HUVECs after stimulation with PM2 (Ceunynck et al. 2018).

We included the new data in the results section on page 6 and 7 and Fig. EV2F and added to the discussion page 14 that there are probably downstream events of THR/PAR1 that are not changed when PM2 is present: "However, we extended of the aPC-like signaling of PM2 remains questionable, since we did not observe AKT activation as reported in endothelial cells (Ceunynck et al. 2018). There might be further signaling events downstream of PAR1 that remain unaltered by PM2 in CD34+ cells, such as Gα12/13 mediated RhoA activation by THR (Aisiku et al. 2015; Künze and Isermann 2023), which we did not investigate here."

Question 3) The primary transplantation endpoint was 4 weeks post-transplant, which primarily reflects short-term repopulating cells rather than long-term HSCs. Although secondary transplantation data provide evidence for long-term repopulating capacity, could the authors extend the primary observation period to 16 weeks to confirm that aPC truly protects long-term HSCs? If chimerism in aPC-treated groups remains stable or increases over time, this would provide compelling evidence for sustained HSC function.

Answer 3) We thank the referee for this thoughtful suggestion. We agree that secondary transplantation provides evidence for long-term repopulation capacity. We respectfully argue that serial transplantation represents a more stringent and functionally relevant assessment of self-renewing HSC activity and frequencies, since it requires the cells to re-engraft, self-renew, and regenerate multilineage hematopoiesis in a new host. In NBSGW mice in particular, human chimerism reaches a plateau within 6-8 weeks (McIntosh et al. 2015; Cosgun et al. 2014), limiting the ability to resolve long-term and sustained HSC function. Long-term engraftment of human HSCs in immunodeficient mice is also less clearly defined compared to murine transplantation system (Guezguez and Bhatia 2008).

We therefore decided to focus on early primary engraftment (4 weeks) to see faster human contribution and assess the serial repopulation capacity with secondary transplantation. We propose that these early timepoint findings have important clinical implications, suggesting that patients undergoing HSC transplantation could potentially experience accelerated hematopoietic recovery and less complications. Furthermore, prior studies have indicated that most human MPPs and committed progenitors do not persist beyond early timepoints and fail to contribute to secondary engraftment in xenografts (Notta et al. 2011; Majeti et al. 2007). Therefore, secondary transplantation selectively measures HSCs with durable self-renewal and multilineage reconstitution capacity (Park et al. 2008; Wiekmeijer et al. 2014). Consistent with this, we demonstrate the presence of human CD34+CD38- cells in primary recipients (Fig. 7E) which were three times more abundant in mice that received aPC treated cells. BM cells of these aPC-recipients were also able to establish a CD34+CD38- population in secondary recipients. In contrast, this capacity was reduced in the control group and thrombin stimulated cells, where only in one out of seven mice human CD34+CD38- cells were detectable in the bone marrow . Although we do not show long term engraftment, we can demonstrate the serial engraftment of human HSCs with Lin-CD34+CD38- phenotype that are considered repopulating HSC (Bhatia et al. 1997).

We have complemented our analysis with new data showing that aPC stimulation increases EPCR-expressing CD34+CD38- phenotypic HSCs in the BM of primary recipients (Fig. 7F), substantiating our observed enhanced serial engraftment potential, while PAR1 expression remains equal between all groups. Notably, multilineage engraftment (myeloid, B-, and T-cell lineages) is improved in mice transplanted with aPC-stimulated HSPCs, with a particularly pronounced increase in T-cell output in primary recipients and broadly enhanced mature lineage reconstitution in secondary recipients (Fig. 7J, EV5C, D, E), compared to control- or thrombin-treated CD34+ cells.

In our revised manuscript, we re-emphasized that we do not claim or allude to improved long-term engraftment with aPC treatment of HSPCs, but we rather point out that we see an increase in serial engraftment potential of aPC-stimulated cells which is considerably decreased upon THR stimulation. We further state in the discussion that the lack of long-term engraftment studies is a limitation of our study on page 11 and 12.

The new data are included in the results part on page 10 and 11 and in the Figure 7F and J and EV5C, D, E.

Question 4) The authors showed that EPCR+ HSCs divide more slowly than EPCR- HSCs even under IL-1α stimulation. Could the authors briefly discuss this resistance in Discussion?

Answer 4) We now included a paragraph in the discussion about the resistance of EPCR+ HSCs to IL-1 induced proliferation. The fact that human EPCR+ HSCs divide slower in culture has been observed in the past, however this was performed with sorted EPCR+ HSCs and the phenotype was not followed up during cultivation (Anjos-Afonso et al. 2022). In murine models, the resistance of EPCR towards inflammation was observed in several studies (Rommel et al. 2022; Rabe et al. 2020). This is now part of the discussion on page 13.

In our manuscript presented here, we now confirm these findings in the context of human HSCs *in vitro*. Mechanistically, we identified the downregulation of cell cycle genes in HSPCs after activation with aPC for its ability to block IL-1 induced proliferation.

Question 5) Parts of the Discussion restate results rather than interpreting them. Could the authors streamline the Discussion, focusing on study limitations, comparison with murine studies and future directions. For example, how aPC and thrombin is functioning in vivo steady state hematopoiesis or stress hematopoiesis such as bleeding.

Answer 5) We have streamlined the discussion to focus more on the *in vivo* aspects of aPC/THR signaling on HSCs and deleted parts that repeat our results, as the Reviewer suggested.

In the discussion (see page 13/14 of the manuscript), we now state that *in vivo*, aPC levels in the mouse blood are below 1 ng/ml (Pepler et al. 2015; Zheng et al. 2007), but Protein C levels in contrast are much higher, and its thrombin-induced activation is controlled by EPCR and thrombomodulin. It is therefore likely, that aPC will be generated locally in the BM.

Reduced levels of Protein C may result in elevated inflammation, which has been observed in men (Shaw et al. 2011) and mice (Lay et al. 2005). It can therefore counteract HSC activation in the context of pathologic situation such as increase inflammatory cytokine levels in the BM (see answer 4).

In contrast to the *in vivo* situation, in our experiments, we applied high aPC doses *ex vivo*. Because EPCR's role is mainly PC binding to support its activation, the *ex vivo* PAR1 activation by aPC does not solely rely on EPCR expression on HSPCs (see answer 1).

Similarly, in haemostasis, thrombin is virtually undetectable in the blood, since it is bound to antithrombin or is present in its inactive form, prothrombin (Mann et al. 2003). However, during local coagulation events in the BM, elevated THR levels could also act on HSCs directly or cause inflammation, which in turn activates HSCs. However, studies

investigating this potential axis of HSC activation *in vivo* are missing to our knowledge. This is also now part of our discussion on page 14 of the manuscript.

Minor concerns

The manuscript uses "HSC" and "HSPC" interchangeably. Could the authors clearly define in the Introduction when each term is appropriate?

Answer: The terms HSC and multipotent progenitor (MPP) were defined in the results section of Figure 1 as CD34+CD38-CD45RA-CD90+ and CD34+CD38-CD45RA-CD90-, respectively. We changed the use of HSC and HSPC in multiple instances in the manuscript. In general, we use the terms HSC and HSPCs mostly in reference to other publications using the terms. We rather state the exact marker combination that was used during analysis (e.g. CD34+CD45RA-CD90+ or CD34+CD38-) wherever possible to be precise.

Referee #3 (Remarks for Author):

In their manuscript entitled "Activated Protein C-PAR1 signaling promotes Hematopoietic Stem Cell Quiescence and Engraftment", Pöllmann et al. conduct a methodical analysis of aPC/PAR1 signalling in primary hematopoietic stem cells. Building on many studies which identify EPCR as a key molecule expressed on HSCs (especially in culture) and relatively limited data that goes after the mechanism of the pathway, this study represents a novel take on EPCR/aPC/PAR1 biology that should have strong relevance for the community.

The strength of the study is in its careful step-by-step description of each aspect - first determining expression levels and rates of molecule recycling, second expanding the suite of assays (compared to published studies) exploring the nature of functional effects caused by the aPC/PAR1 axis, and third by careful in vitro and in vivo functional studies. Overall I think the manuscript is really nicely performed and backed by strong scientific thinking.

Answer: We thank the Reviewer for the positive and constructive feedback to improve our manuscript.

I have some minor considerations for the authors which I would hope could strengthen the manuscript if they are not too difficult to incorporate:

Question 1) Orthogonal assays to measure calcium flux - the Ca2+ field has many assays and contradicting results. Have the authors tried any other assays (e.g., Fura molecules, Rhod2, etc)? If so, do they (broadly speaking) show a similar trend as Fig2a? Alternatively, have they considered patch-clamping for measuring rates of change in combination with the dyes? (please note that I wouldn't consider any of this essential to believe the finding, but if it were available or relatively easy to generate, I think it would add strength to the manuscript).

Answer 1) We agree with the Referee, that the results from Ca2+ measurement experiments need to be carefully interpreted, due to the variety of different Ca2+ probes with different cellular compartmentalization, dynamic range and mode of action (fluorometric vs. ratiometric). As the Referee suggested, we repeated our Ca2+ flux in expanded CD34+ cells with the Ca2+ indicator Rhod-2. The results from this experiment are now depicted in the Figure EV2A, E. Like with Fluo-4, Ca2+ flux could only be detected with THR stimulation when using Rhod-2. <u>The revised results section recapitulates this now (page 5/6)</u>. Patch-clamping of HSCs has been described sporadically in literature (Ludwig et al. 2013; Park et al. 2011), but the electrophysiology of HSCs remains largely under-investigated. Since we studied the mobilization of Ca2+ from the ER into the

cytosol, this would require patch clamping the ER of HSCs and not only the whole cell. ER-patch clamping is technically very challenging and is only possible on isolated ER or nuclei. We therefore concluded that it would not be feasible in the timeframe of the revision for the manuscript.

Question 2) Behaviour in mutated cells - several mutations associated with clonal hematopoiesis or leukemia have been indicated to have changes in Ca2+ regulation (in particular TET2-mutated cells). If there is the ability to show that these cells behave differently in HSC-expansion settings, it would give a mechanistic reason for why TET2 mutated cells expand better than WT. (again, I would consider this type of "extra" beyond the scope of the current manuscript, but if it were available, I think it would expand the readership and interest for the manuscript).

Answer 2) We rely in our research work on commercially available CD34+ cells and therefore unfortunately have no information about their TET2 mutation state. We also do not have access to clinical samples of TET2-mutated CD34+ cells to test, whether their Ca2+ regulation is altered and whether this contributes to their expansion. However, we recognize the value of exploring this mechanistic pathway and conducted experiments in our model to gage, if THR or aPC altered protein levels of TET2 and influences global 5-hydroxymethylcytosine (5-hmC), since it is the product of TET2 activity and marks demethylation. A Ca2+-Calpain-TET axis was described in HSCs by Luchsinger et al. (Luchsinger et al. 2019), where high Ca2+ concentration during HSC cultivation causes calpain activation, degrading TET and thereby preventing demethylation, ultimately causing HSC exhaustion. aPC has also been described to reduce global 5-metyhl cytosine (5-mC) in T cells (Gupta et al. 2023). We could not detect any changes in TET2 protein nor in 5-hmC levels after continuous aPC or THR stimulation over 1 week (Fig. EV4F,G). However, altered Ca2+ levels due to Thapsigargin treatment decreased TET2 and 5hmC levels (Fig. EV4F, G) and increased differentiation of CD34+ cells (Fig. EV4H). We also investigated whether Thapsigargin causes changes in cell proliferation, see answer to the next question. <u>The data were included in the results part pages 9, and in the Figure EV4.</u>

Question 3) With the conclusion on page 6 about quiescent v cycling HSCs, could the authors potentially assess fetal liver HSCs (actively cycling, EPCR+) in mouse to see how thapsigargin hits them? Maybe it would drive them into quiescence?

Answer 3) The Referee raises an interesting point: in the murine fetal liver, EPCR+HSCs are indeed predominantly cycling (Iwasaki et al. 2010), unlike during the adult hematopoiesis in the BM. In this project we aim to elucidate the signaling of aPC and THR on adult human HSPCs, therefore introducing fetal HSCs would take away from the focus of our manuscript. We did however investigate the effects of Thapsigargin on adult human

CD34+ cells in culture, which are also cycling and to some degree EPCR+. As pointed out in our answer to question 2, Thapsigargin severely restricted proliferation over 1 week (Fig. EV4H), but had detrimental effects on CD34+ cells, like reduction of percentage CD34+ cells, indicating differentiation (Fig. EV4I), see also the answer to the previous question. The data are included in the results part page 9 and Fig. EV4H.

Question 4) The authors are very careful with their language (which is great) regarding the conclusion: "aPC promoted clonogenic potential and maintained undifferentiated HSPCs in vitro." That said, the HSC field will be interested in whether short term treatment leads to expanded functional HSCs and secondary colony assays do not demonstrate this (although they indicate that it would be possible). Ideally a transplantation accompanies this experiment. Where they do conduct a transplantation, it is only after a very brief exposure and culture of human HSPCs (1h) and a very brief transplantation window (4 weeks primary, 4 weeks secondary). I would therefore suggest that the terminology and language around the experiments and conclusions on page 10 are tempered. The data show nicely that there is an effect on the stem/progenitor pool, but the assay does not demonstrate "the fundamental role of PAR1 signaling in HSC" or show that "a single dose of aPC maintained HSC activity even after serial transplantation."

Answer 4) As pointed out in our answer to Referee 2, Question 3, we did not conduct long-term engraftment experiments, we did however now include more data about multilineage engraftment in the BM of 1° and 2° recipient mice, proofing that aPC also enables multilineage engraftment in all 2° mice. We also now state in the discussion that the lack of long-term engraftment studies is a key limitation of our study on page 11. In accordance with the Referee, we tempered the language in the discussion and results and changed the sentence on to: "Taken together, the opposing effects of aPC and thrombin on HSCs influence their serial engraftment potential, where a single dose of aPC maintained HSCs and enabled serial multilineage differentiation capacity." (see page 11 in the results part).

Miscellaneous changes to the originally submitted manuscript:

We corrected typos and grammatical errors and changed the wording in several instanced to better reflect our findings. New text is colored red, strikethrough text indicates deleted passages.

The formatting guidelines of EMBO Molecular Medicine were followed, leading to creation of extended view figures (EVs) and Appendix figures.

During revision, we noticed that in Figure 4B and Supplemental figure 4B we made a minor error when transferring data, leading to incorrect calculation of the acute response

of CD34+ cells in the Mitostress test. All other metabolic parameters were correctly calculated from the beginning. We have corrected this error and updated the Figure 4B and Appendix Figure S3B. The correct values did lead to changes in significance levels; however the overall finding remains unchanged; therefore, there are no changes in the text.

Publication bibliography

Aisiku, Omozuanvbo; Peters, Christian G.; Ceunynck, Karen de; Ghosh, Chandra C.; Dilks, James R.; Fustolo-Gunnink, Susanna F. et al. (2015): Parmodulins inhibit thrombus formation without inducing endothelial injury caused by vorapaxar. In *Blood* 125 (12), pp. 1976–1985. DOI: 10.1182/blood-2014-09-599910.

Anjos-Afonso, Fernando; Buettner, Florian; Mian, Syed A.; Rhys, Hefin; Perez-Lloret, Jimena; Garcia-Albornoz, Manuel et al. (2022): Single cell analyses identify a highly regenerative and homogenous human CD34+ hematopoietic stem cell population. In *Nature communications* 13 (1), p. 2048. DOI: 10.1038/s41467-022-29675-w.

Bhatia, M.; Wang, J. C.; Kapp, U.; Bonnet, D.; Dick, J. E. (1997): Purification of primitive human hematopoietic cells capable of repopulating immune-deficient mice. In *Proceedings of the National Academy of Sciences of the United States of America* 94 (10), pp. 5320–5325. DOI: 10.1073/pnas.94.10.5320.

Ceunynck, Karen de; Peters, Christian G.; Jain, Abhishek; Higgins, Sarah J.; Aisiku, Omozuanvbo; Fitch-Tewfik, Jennifer L. et al. (2018): PAR1 agonists stimulate APC-like endothelial cytoprotection and confer resistance to thromboinflammatory injury. In *Proceedings of the National Academy of Sciences of the United States of America* 115 (5), E982-E991. DOI: 10.1073/pnas.1718600115.

Cosgun, Kadriye Nehir; Rahmig, Susann; Mende, Nicole; Reinke, Sören; Hauber, Ilona; Schäfer, Carola et al. (2014): Kit regulates HSC engraftment across the human-mouse species barrier. In *Cell stem cell* 15 (2), pp. 227–238. DOI: 10.1016/j.stem.2014.06.001.

Guezguez, Borhane; Bhatia, Mickie (2008): Transplantation of human hematopoietic repopulating cells: mechanisms of regeneration and differentiation using human-mouse xenografts. In *Current opinion in organ transplantation* 13 (1), pp. 44–52. DOI: 10.1097/MOT.0b013e3282f42486.

Gupta, Dheerendra; Elwakiel, Ahmed; Ranjan, Satish; Pandey, Manish Kumar; Krishnan, Shruthi; Ambreen, Saira et al. (2023): Activated protein C modulates T-cell metabolism and epigenetic FOXP3 induction via α-ketoglutarate. In *Blood advances* 7 (17), pp. 5055–5068. DOI: 10.1182/bloodadvances.2023010083.

Iwasaki, Hiroko; Arai, Fumio; Kubota, Yoshiaki; Dahl, Maria; Suda, Toshio (2010): Endothelial protein C receptor-expressing hematopoietic stem cells reside in the perisinusoidal niche in fetal liver. In *Blood* 116 (4), pp. 544–553. DOI: 10.1182/blood-2009-08-240903.

Künze, Georg; Isermann, Berend (2023): Targeting biased signaling by PAR1: function and molecular mechanism of parmodulins. In *Blood* 141 (22), pp. 2675–2684. DOI: 10.1182/blood.2023019775.

Lay, Angelina J.; Liang, Zhong; Rosen, Elliot D.; Castellino, Francis J. (2005): Mice with a severe deficiency in protein C display prothrombotic and proinflammatory phenotypes and compromised maternal reproductive capabilities. In *The Journal of clinical investigation* 115 (6), pp. 1552–1561. DOI: 10.1172/JCI24030.

Luchsinger, Larry L.; Strikoudis, Alexandros; Danzl, Nichole M.; Bush, Erin C.; Finlayson, Michael O.; Satwani, Prakash et al. (2019): Harnessing Hematopoietic Stem Cell Low Intracellular Calcium Improves Their Maintenance In Vitro. In *Cell stem cell* 25 (2), 225-240.e7. DOI: 10.1016/j.stem.2019.05.002.

Ludwig, M.; Skorska, A.; Tölk, A.; Hopp, H-H; Patejdl, R.; Li, J. et al. (2013): Characterization of ion currents of murine CD117(pos) stem cells in vitro and their modulation under AT2 R stimulation. In *Acta physiologica (Oxford, England)* 208 (3), pp. 274–287. DOI: 10.1111/apha.12115.

Majeti, Ravindra; Park, Christopher Y.; Weissman, Irving L. (2007): Identification of a hierarchy of multipotent hematopoietic progenitors in human cord blood. In *Cell stem cell* 1 (6), pp. 635–645. DOI: 10.1016/j.stem.2007.10.001.

Mann, K. G.; Brummel, K.; Butenas, S. (2003): What is all that thrombin for? In *Journal of thrombosis and haemostasis : JTH* 1 (7), pp. 1504–1514. DOI: 10.1046/j.1538-7836.2003.00298.x.

McIntosh, Brian E.; Brown, Matthew E.; Duffin, Bret M.; Maufort, John P.; Vereide, David T.; Slukvin, Igor I.; Thomson, James A. (2015): Nonirradiated NOD,B6.SCID Il2rγ-/- Kit(W41/W41) (NBSGW) mice support multilineage engraftment of human hematopoietic cells. In *Stem cell reports* 4 (2), pp. 171–180. DOI: 10.1016/j.stemcr.2014.12.005.

Notta, Faiyaz; Doulatov, Sergei; Laurenti, Elisa; Poeppl, Armando; Jurisica, Igor; Dick, John E. (2011): Isolation of single human hematopoietic stem cells capable of long-term multilineage engraftment. In *Science (New York, N.Y.)* 333 (6039), pp. 218–221. DOI: 10.1126/science.1201219.

Park, Christopher Y.; Majeti, Ravindra; Weissman, Irving L. (2008): In vivo evaluation of human hematopoiesis through xenotransplantation of purified hematopoietic stem cells from umbilical cord blood. In *Nature protocols* 3 (12), pp. 1932–1940. DOI: 10.1038/nprot.2008.194.

Park, Kyoung Sun; Pang, Bo; Park, Su Jung; Lee, Yun-Gyoo; Bae, Ji-Yeon; Park, Seonyang et al. (2011): Identification and functional characterization of ion channels in CD34(+) hematopoietic stem cells from human peripheral blood. In *Molecules and cells* 32 (2), pp. 181–188. DOI: 10.1007/s10059-011-0068-9.

Pepler, Laura; Yu, Pei; Dwivedi, Dhruva J.; Trigatti, Bernardo L.; Liaw, Patricia C. (2015): Characterization of mice harboring a variant of EPCR with impaired ability to bind protein C: novel role of EPCR in hematopoiesis. In *Blood* 126 (5), pp. 673–682. DOI: 10.1182/blood-2014-02-558940.

Rabe, Jennifer L.; Hernandez, Giovanny; Chavez, James S.; Mills, Taylor S.; Nerlov, Claus; Pietras, Eric M. (2020): CD34 and EPCR coordinately enrich functional murine hematopoietic stem cells under normal and inflammatory conditions. In *Experimental hematology* 81, 1-15.e6. DOI: 10.1016/j.exphem.2019.12.003.

Rommel, Marcel G. E.; Walz, Lisa; Fotopoulou, Foteini; Kohlscheen, Saskia; Schenk, Franziska; Miskey, Csaba et al. (2022): Influenza A virus infection instructs hematopoiesis to megakaryocyte-lineage output. In *Cell reports* 41 (1), p. 111447. DOI: 10.1016/j.celrep.2022.111447.

Shaw, Andrew D.; Vail, George M.; Haney, Douglas J.; Xie, Jin; Williams, Mark D. (2011): Severe protein C deficiency is associated with organ dysfunction in patients with severe sepsis. In *Journal of critical care* 26 (6), pp. 539–545. DOI: 10.1016/j.jcrc.2011.05.006.

Wiekmeijer, Anna-Sophia; Pike-Overzet, Karin; Brugman, Martijn H.; Salvatori, Daniela C. F.; Egeler, R. Maarten; Bredius, Robbert G. M. et al. (2014): Sustained Engraftment of Cryopreserved Human Bone Marrow CD34(+) Cells in Young Adult NSG Mice. In *BioResearch open access* 3 (3), pp. 110–116. DOI: 10.1089/biores.2014.0008.

Zheng, X.; Li, W.; Song, Y.; Hu, Y.; Ferrell, G. L.; Esmon, N. L.; Esmon, C. T. (2007): Non-hematopoietic EPCR regulates the coagulation and inflammatory responses during endotoxemia. In *Journal of thrombosis and haemostasis : JTH* 5 (7), pp. 1394–1400. DOI: 10.1111/j.1538-7836.2007.02592.x.

22nd Jul 2026

Dear Prof. Modlich,

Thank you for submitting your revised manuscript, which has been reviewed by referees #2 and #3. As you will see from their reports below, they are overall satisfied with the revisions, and I will therefore be able to accept your manuscript pending the following minor revisions:

1/ Referees' concerns:
Please address the remaining mechanistic concerns either experimentally, where feasible, or by appropriately discussing the limitations, toning down the mechanistic conclusions, and revising statements regarding the proposed signaling mechanism to ensure they are fully supported by the data (including in the abstract). Please also address the additional minor referees' concerns.

2/ Manuscript text:
- Please remove the red font text, and only keep in track changes any new modification.
- Please define corresp. author on title page and add email address.
- Please update the order and the headings of the manuscript sections to: Abstract / The Paper Explained / Introduction / Results / Discussion / Methods / Data Availability / Acknowledgements / Disclosure and Competing Interests Statement / References / Figure Legends / Expanded View Figure Legends
- Please change the heading from "Materials and Methods" to "Methods":
o Please include the methods currently in the Appendix in the main manuscript text file.
o Animals: State details of authority granting ethics approval (IRB or equivalent committee(s), provide reference number for approval. Include a statement of compliance with ethical regulations.
o Human materials: clarify if the source is commercial, or include a statement confirming that the experiments conformed to the principles set out in the WMA Declaration of Helsinki and the Department of Health and Human Services Belmont Report and state details of authority granting ethics approval (IRB or equivalent committee(s), provide reference number for approval.
o Statistics: Please provide a statement on randomization and inclusion/exclusion criteria
- Data Availability Section: remove "For original data, please contact Ute Modlich (ute.modlich@irem.uzh.ch)". Please note that the deposited datasets must be public before acceptance of the manuscript.
- Acknowledgements: the information provided in the submission system should match the information provided in the manuscript. Currently, 1S10OD030423-01 and S10OD036313 are not listed in the list of funders in our system.
- Remove the author contribution from the manuscript, and provide CRediT (Contributor Role Taxonomy) terms in the submission system. These replace a narrative author contribution section in the manuscript.
- Please change "Conflict of interest disclosure" to "Disclosure and competing interests statement".

3/ Figures:
- Appendix: Please correct to "Appendix Table S1" and "Appendix Table S2"; move the appendix methods to the main manuscript. You are welcome to make your figures S2, S3 and S4 into EV figures.
- Please address the queries from our data editors in the figure legends:
1. Please note that the exact p values are not provided in the legends of figures 1B, C, D, E, F, G; 2A, C, D, F; 4B, C, D, F, H, I; 5A-E; 6A, C, D, E, F; 7C, E, F, G, I, J; EV1 B, C, D, E, F, G; EV2 E, F, G; 2C, D; 3B, 4B
2. Please indicate the statistical test used for data analysis in the legends of figures EV3 C, D
3. Please note that the box plots need to be defined in terms of minima, maxima, centre, bounds of box and whiskers, and percentile in the legends of figures EV3 A
4. Please note that information related to n is missing in the legends of figures EV3 A, C, D

4/ In the checklist, Experimental study design and statistics, please fill in all subsections.

5/ Source Data: We would encourage you to provide the raw facs data alongside the excel files.

6/ Visual abstract: please provide as a jpg or png format, with the dimensions 550 pixels wide x 300 pixels high. Please ensure the text remains legible.

7/ As part of the EMBO Publications transparent editorial process initiative (see our Editorial at http://embomolmed.embopress.org/content/2/9/329), EMBO Molecular Medicine will publish online a Review Process File (RPF) to accompany accepted manuscripts.
This file will be published in conjunction with your paper and will include the anonymous referee reports, your point-by-point response and all pertinent correspondence relating to the manuscript. Let us know whether you agree with the publication of the RPF.
Please note that the Authors checklist will be published at the end of the RPF.

I look forward to receiving your revised manuscript.

Yours sincerely,

Lise Roth

Lise Roth, PhD
Senior Editor
EMBO Molecular Medicine

***** Reviewer's comments *****

Referee #2 (Remarks for Author):

This manuscript presents an interesting and technically rigorous study demonstrating that distinct PAR1 signaling programs differentially regulate human HSPC function. The transplantation studies, together with the signaling and metabolic analyses, provide a solid body of evidence supporting the biological importance of biased PAR1 signaling. The revised manuscript has clearly improved following revision. The remaining concerns are primarily related to mechanistic interpretation rather than experimental quality. In particular, the conclusions regarding AKT/CREB-mediated protection and EPCR-independent signaling should better reflect the level of evidence currently provided. Addressing these issues would further strengthen the manuscript and improve the accuracy of its mechanistic conclusions.

Major concerns

1. The role of AKT/CREB signaling in mediating the protective effects of aPC remains unproven. The central mechanistic model proposed by the authors is that biased activation of PAR1 by aPC promotes HSC maintenance through preferential activation of AKT and CREB signaling. However, the current data remain largely correlative. Although increased phosphorylation of AKT and CREB is clearly demonstrated, no experiments establish whether these signaling pathways are required for the beneficial effects of aPC on HSC maintenance, quiescence, or engraftment. Consequently, the manuscript demonstrates that AKT/CREB activation is associated with improved HSC function, but it does not establish causality.

2. The revised manuscript appropriately discusses the possibility that the relatively high concentration of aPC used in this study may activate PAR1 independently of EPCR. However, this interpretation is still not directly supported experimentally. Since EPCR remains a central component of the proposed signaling model, it would be valuable to determine whether aPC-induced downstream signaling differs between EPCR-positive and EPCR-negative HSPCs or whether inhibition of EPCR alters the biological response to aPC.

Minor concerns
1. The manuscript still alternates between the terms "HSC" and "HSPC." More consistent terminology would improve clarity, particularly when conclusions specifically refer to long-term repopulating HSCs rather than heterogeneous CD34+ progenitor populations.

2. Several paragraphs in the Discussion continue to summarize experimental observations rather than interpreting their biological implications. Further streamlining would improve readability and place greater emphasis on the significance and limitations of the study.

Referee #3 (Remarks for Author):

The authors have addressed the majority of my concerns with only one outstanding comment from my side regarding the transplantation experiments:

The authors make arguments that multi-lineage capacity for their short length serial transplants equates to an HSC activity. While they do acknowledge the longterm limitation now, I would prefer the use of the term HSPCs (e.g. stem/progenitor) just to

avoid conflating the population and properties with proper LT-HSCs which have substantially different dynamics in transplant settings - e.g., In Belluschi/Laurenti Nat Comms 2018, the LT-HSCs do not even really start producing until the 20 week time point.

Therefore I would suggest two things:
1) alter wording to talk about effects of aPC/thrombin on HSPCs (as opposed to HSCs)
2) change all instances of serial engraftment or serial engraftment potential to "short term" or "short duration" serial engraftment so it does not get confused with the HSC property of "serial engraftment" which will be assumed to be >12 weeks (and in some cases >20 weeks).

Point-by-Point Response 2nd resubmission

Referee #2 (Remarks for Author):

This manuscript presents an interesting and technically rigorous study demonstrating that distinct PAR1 signaling programs differentially regulate human HSPC function. The transplantation studies, together with the signaling and metabolic analyses, provide a solid body of evidence supporting the biological importance of biased PAR1 signaling. The revised manuscript has clearly improved following revision. The remaining concerns are primarily related to mechanistic interpretation rather than experimental quality. In particular, the conclusions regarding AKT/CREB-mediated protection and EPCR-independent signaling should better reflect the level of evidence currently provided. Addressing these issues would further strengthen the manuscript and improve the accuracy of its mechanistic conclusions.

Answer: We thank the Referee for the constructive feedback.

Major concerns

Question 1) The role of AKT/CREB signaling in mediating the protective effects of aPC remains unproven. The central mechanistic model proposed by the authors is that biased activation of PAR1 by aPC promotes HSC maintenance through preferential activation of AKT and CREB signaling. However, the current data remain largely correlative. Although increased phosphorylation of AKT and CREB is clearly demonstrated, no experiments establish whether these signaling pathways are required for the beneficial effects of aPC on HSC maintenance, quiescence, or engraftment. Consequently, the manuscript demonstrates that AKT/CREB activation is associated with improved HSC function, but it does not establish causality.

Answer 1) In our study we show that the treatment of human HSPC with aPC leads to distinct signaling, improved HSPC maintenance in vitro and enhanced short-term human engraftment in NBSGW mice. We agree with the Referee that we did not experimentally prove whether the aPC mediated and PAR1 induced AKT and CREB phosphorylation was causative for the beneficial effects on HSPC function.

We think that there are several approaches one could follow to test our proposed model that the aPC induces HSC maintaining signals via PAR1 by activating AKT and CREB but none appeared to us to be immediately feasible:

As the aPC activated downstream molecules of PAR1 were AKT and CREB, the inhibition of these molecules may answer some aspects. However, as AKT and CREB have been described downstream of important cytokine receptors presented on HSC, such as MPL or KIT (Wang et al. 2021; Tsutsumi et al. 2023; Yamazaki et al. 2006) their pharmacological inhibition or knock-out/knock-down would also affect other cytokine signaling, necessary for in vitro and in vivo HSPC maintenance. We expect that the effects will be broad and will not allow to specifically address our question. We therefore decided against performing these experiments.

Another option would be to mutate PAR1 in a way that one of the downstream targets cannot be activated. To our knowledge, two PAR1 mutants that render PAR1 to not recruit specific Gα subunit have been described (McCoy et al. 2012; Soto et al. 2015). However of these two, one (PAR1-R205A) prevents Gaq signaling, which is responsible for Ca2+ flux and did not alter ERK1/2 activation (McCoy et al. 2012). The other PAR1 mutant (PAR1 NA ECL2) reduced Ga12/13 and Gaq recruitment, responsible for RhoA activation and Ca2+ flux respectively (Soto et al. 2015). Apart from those, no known specific PAR1 mutants are described that could be used to selectively prevent AKT, ERK1/2 or CREB activation and investigate whether any of these downstream targets play a role in HSPC function. Finding new suitable PAR1 mutants would require extensive mutation studies and introducing these PAR1 mutants in primary

hematopoietic cells, like CD34+ cells and simultaneously knocking out or down the native PAR1 is also technically challenging. Therefore, also this approach did not appear feasible.

Alternatively, we considered to mutate the aPC cleavage site at PAR1. Respective mutants have been described (Schuepbach et al. 2012; Sinha et al. 2018). While this mutation in PAR1 would not abolish one specific downstream signal, one could at least demonstrate that aPC mediates its beneficial effects in HSPC indeed by cleaving and thereby activating PAR1, inducing signaling. While the overexpression of such mutant may be straight forward e.g. by using lentiviral vectors, the co-expression of the wildtype PAR1 will interfere with the readout. As today, mutations can be introduced in a targeted manner more easily using gene editing technologies, new options became available to mutate the endogenous PAR1, but efficacy may be limited considering that we aim to study effects in human CD34+ HSPC from apheresis donors and these cells cannot be selected for the appropriate mutation before assay start.

Lastly, there are small molecules, similar to parmodulin, that may activate only selected pathways of PAR1 signaling. So far, we investigated only parmodulin 2 (PM2). Stimulation of CD34+ cells with PM2 activated CREB similar to THR and aPC, but only minimally AKT and not ERK1/2 (EV3A), indicating a different PAR1 activation potential. On the other hand, PM2 inhibited THR induced ERK1/2 and CREB activation (Fig. 2 F and H) and rescued the negative effects of THR stimulation om human engraftment (Fig.7). Considering this information, one may indeed conclude that the negative impact of hematopoietic engraftment induced by THR was mediated at least in part by ERK1/2 phosphorylation.

We have to consider, that there are also other signaling pathways downstream of PAR1 which we did not investigate. THR activates for example small GTPases, which regulate cytoskeleton organisation and cell migration. The quantification of GTPase activity is technical challenging, especially in primary HSPC. Gur-Cohen and colleagues have reported that in murine HSCs, THR activates CDC42 and causes loss of CDC42-polarization, contributing to HSC mobilization (Gur-Cohen et al. 2015). Our scRNAseq also indicates that small GTPase pathways are indeed activated in CD34+ cells in response to aPC and THR, since GO terms involved in actin and cytoskeleton regulation were upregulated after 4 hours treatment with the activators (Fig. 3E).

Our analysis could be expanded on further parmodulins (Künze and Isermann 2023). Alternatively, pepducins, palmitated peptides, can act as PAR1 activators or inhibitors (Covic et al. 2002; Zhang et al. 2015), these could provide further insights into which signaling pathway may be most responsible for the observed changes in HSPC function.

Taken together, we agree that this is an important question that should be explored in the future, but we also believe that it will be outside to scope of the current paper. We therefore followed the referee's advice to tone down our interpretation throughout the manunscript text, but avoiding phrases like "PAR1 signaling induced effects" (e.g. on page 11) and also comment on it in the discussion on page 14-15, by stating: "*Although we cannot causally link the observed PAR1 downstream signaling events after aPC and THR stimulation to the functional changes in CD34+ HSPCs, our study characterises which PAR1 signaling pathways are activated in human HSPCs in vitro and presents evidence of the supportive role of aPC treatment of CD34+ on their short-term engraftment in mice*." Furthermore, we changed the manuscript title to better reflect this to "Activated Protein C promotes Hematopoietic Stem and Progenitor Cell Quiescence and Engraftment".

Question 2) The revised manuscript appropriately discusses the possibility that the relatively high concentration of aPC used in this study may activate PAR1 independently of EPCR.

However, this interpretation is still not directly supported experimentally. Since EPCR remains a central component of the proposed signaling model, it would be valuable to determine whether aPC-induced downstream signaling differs between EPCR-positive and EPCR-negative HSPCs or whether inhibition of EPCR alters the biological response to aPC.

Answer 2) We thank the referee for reminding on this important issue. In response to the previous review, we already conducted experiments addressing this question. The results of these experiments were included in the Figure EV1F, G and EV3 B, C, D and the corresponding results section on pages 6-8. We realize that we may have not explained the experiments well enough and would like to do this here:

We performed experiments where we incubated CD34+ cells with EPCR-blocking antibodies, stimulated them with aPC and measured the phosphorylation of PAR1 downstream targets by phosphoflow. To ensure the blocking potential of the antibodies, we first validate whether the antibodies and aPC compete for binding on EPCR. In this test, we stimulated CD34+ cells with aPC or left them unstimulated. In the subsequent staining with antibodies (we tested three commercially available clones) we observed that in all cases the samples that were treated with aPC before the staining procedure had a reduced percentage of EPCR+ cells (Fig. EV1F).

As this experiment validated the competition of antibodies and aPC for EPCR binding, it also made it technically difficult to perform aPC stimulation experiments and simultaneous analysis of EPCR expression.

We followed this up by incubating CD34+ cells with one EPCR-blocking antibody (clone REA337) for 1 hour at 37°C, followed by stimulation with aPC. We measured presence of PAR1 on the cell surface and could see, that blocking EPCR did not result in loss of PAR1 internalization after aPC stimulation (Fig. EV1G). This experiment proved that aPC was able to activate PAR1 which leads to PAR1internalisation although aPC could not bind to EPCR. We then measured the phosphorylation of CREB and AKT in CD34+ cells that were incubated with the EPCR-blocking antibody. We detected the same level of AKT phosphorylation in EPCR-blocked CD34+ compared to CD34+ cells not treated with the anti-EPCR antibody (FIG. EV3D), however the level of CREB phosphorylation in EPCR-blocked CD34+ cells was less strong (Fig. EV3C).

In a second approach, we sorted EPCR-pos. and EPCR-neg. CD34+ cells (Fig. EV3B). Because staining of EPCR to be able to sort the cells also block EPCR, we then compared the EPCR-neg CD34+ cells with non-sorted, non-stained CD34+ cells. After stimulation with aPC we again performed phosphoflow analysis. In these experiments we saw equal levels of AKT phosphorylation in EPCR-neg. CD34+ compared to none sorted CD34+ cells, but lower levels of CREB phosphorylation, very similar to our observations in CD34+ cells that were incubated with the EPCR-blocking antibody (Fig. EV3C/D).

We interpret these results the following: EPCR expression is not a prerequisite of PAR1 activation and PAR1 signaling *in vitro* in CD34+ cells with aPC at a high concentration (10 ug/ml), however the binding of aPC to EPCR may lead to more efficient activation of at least one PAR1 downstream target.

Minor concerns

Question 1) The manuscript still alternates between the terms "HSC" and "HSPC." More consistent terminology would improve clarity, particularly when conclusions specifically refer to long-term repopulating HSCs rather than heterogeneous CD34+ progenitor populations.

Answer 1) We have now changed the manuscript to be more consistent in the term HSPC and HSC. HSC is now not mentioned in the context of our transplantation experiments and we added the cell surface phenotype in multiple instances to clarify which cell population is described. We also changed the title of the manuscript to “Activated Protein C promotes Hematopoietic Stem and Progenitor Cell Quiescence and Engraftment”.

Question 2) Several paragraphs in the Discussion continue to summarize experimental observations rather than interpreting their biological implications. Further streamlining would improve readability and place greater emphasis on the significance and limitations of the study.

Answer 2) Following the advice, we restructured the discussion to improve its readability. We also discuss the limitations of our study in further detail on page 14-15, by clearly stating that we cannot causally link observed signaling to observed changes in CD34+ HSPC function, as discussed above.

Referee #3 (Remarks for Author):

The authors have addressed the majority of my concerns with only one outstanding comment from my side regarding the transplantation experiments:

The authors make arguments that multi-lineage capacity for their short length serial transplants equates to an HSC activity. While they do acknowledge the longterm limitation now, I would prefer the use of the term HSPCs (e.g. stem/progenitor) just to avoid conflating the population and properties with proper LT-HSCs which have substantially different dynamics in transplant settings - e.g., In Belluschi/Laurenti Nat Comms 2018, the LT-HSCs do not even really start producing until the 20 week time point.

Therefore I would suggest two things:

1) alter wording to talk about effects of aPC/thrombin on HSPCs (as opposed to HSCs)

2) change all instances of serial engraftment or serial engraftment potential to "short term" or "short duration" serial engraftment so it does not get confused with the HSC property of "serial engraftment" which will be assumed to be >12 weeks (and in some cases >20 weeks).

Answer: We thank the referee for the positive feedback and the suggestions on rewording to address the two important issues. We followed the advice and use HSPC rather than HSC.

We rephrased the text in the Results section on pages 11-12 and Discussion to avoid the term HSC also in the context of our transplantation experiments, instead referring to the observed cell surface phenotypes (e.g. CD34+ or CD34+CD38-). We also clarify in all instances that our experiments in mice had short-term follow-ups.

References

Anjos-Afonso, Fernando; Buettner, Florian; Mian, Syed A.; Rhys, Hefin; Perez-Lloret, Jimena; Garcia-Albornoz, Manuel et al. (2022): Single cell analyses identify a highly regenerative and homogenous human CD34+ hematopoietic stem cell population. In *Nature communications* 13 (1), p. 2048. DOI: 10.1038/s41467-022-29675-w.

Bisht, Kavita; Barbier, Valérie; Shatunova, Svetlana; Winkler, Ingrid G.; Lévesque, Jean-Pierre (2026): Endothelial protein C receptor CD201 is a better marker than stem cell antigen-1 to identify mouse long-term reconstituting hematopoietic stem cells following septic challenge. In *Experimental hematology* 154, p. 105326. DOI: 10.1016/j.exphem.2025.105326.

Chagraoui, Jalila; Lehnertz, Bernhard; Girard, Simon; Spinella, Jean Francois; Fares, Iman; Tomellini, Elisa et al. (2019): UM171 induces a homeostatic inflammatory-detoxification response supporting human HSC self-renewal. In *PloS one* 14 (11), e0224900. DOI: 10.1371/journal.pone.0224900.

Covic, Lidija; Gresser, Amy L.; Talavera, Joyce; Swift, Steven; Kuliopulos, Athan (2002): Activation and inhibition of G protein-coupled receptors by cell-penetrating membrane-tethered peptides. In *Proceedings of the National Academy of Sciences of the United States of America* 99 (2), pp. 643–648. DOI: 10.1073/pnas.022460899.

Gur-Cohen, Shiri; Itkin, Tomer; Chakrabarty, Sagarika; Graf, Claudine; Kollet, Orit; Ludin, Aya et al. (2015): PAR1 signaling regulates the retention and recruitment of EPCR-expressing bone marrow hematopoietic stem cells. In *Nature medicine* 21 (11), pp. 1307–1317. DOI: 10.1038/nm.3960.

Künze, Georg; Isermann, Berend (2023): Targeting biased signaling by PAR1: function and molecular mechanism of parmodulins. In *Blood* 141 (22), pp. 2675–2684. DOI: 10.1182/blood.2023019775.

McCoy, Kelly L.; Gyoneva, Stefka; Vellano, Christopher P.; Smrcka, Alan V.; Traynelis, Stephen F.; Hepler, John R. (2012): Protease-activated receptor 1 (PAR1) coupling to G(q/11) but not to G(i/o) or G(12/13) is mediated by discrete amino acids within the receptor second intracellular loop. In *Cellular signalling* 24 (6), pp. 1351–1360. DOI: 10.1016/j.cellsig.2012.01.011.

Munz, Clara M.; Dressel, Nicole; Chen, Minyi; Grinenko, Tatyana; Roers, Axel; Gerbaulet, Alexander (2023): Regeneration after blood loss and acute inflammation proceeds without contribution of primitive HSCs. In *Blood* 141 (20), pp. 2483–2492. DOI: 10.1182/blood.2022018996.

Rabe, Jennifer L.; Hernandez, Giovanny; Chavez, James S.; Mills, Taylor S.; Nerlov, Claus; Pietras, Eric M. (2020): CD34 and EPCR coordinately enrich functional murine hematopoietic stem cells under normal and inflammatory conditions. In *Experimental hematology* 81, 1-15.e6. DOI: 10.1016/j.exphem.2019.12.003.

Rommel, Marcel G. E.; Walz, Lisa; Fotopoulou, Foteini; Kohlscheen, Saskia; Schenk, Franziska; Miskey, Csaba et al. (2022): Influenza A virus infection instructs hematopoiesis to megakaryocyte-lineage output. In *Cell reports* 41 (1), p. 111447. DOI: 10.1016/j.celrep.2022.111447.

Schuepbach, R. A.; Madon, J.; Ender, M.; Galli, P.; Riewald, M. (2012): Protease-activated receptor-1 cleaved at R46 mediates cytoprotective effects. In *Journal of thrombosis and haemostasis : JTH* 10 (8), pp. 1675–1684. DOI: 10.1111/j.1538-7836.2012.04825.x.

Sinha, Ranjeet K.; Wang, Yaoming; Zhao, Zhen; Xu, Xiao; Burnier, Laurent; Gupta, Naveen et al. (2018): PAR1 biased signaling is required for activated protein C in vivo benefits in sepsis and stroke. In *Blood* 131 (11), pp. 1163–1171. DOI: 10.1182/blood-2017-10-810895.

Soto, Antonio G.; Smith, Thomas H.; Chen, Buxin; Bhattacharya, Supriyo; Cordova, Isabel Canto; Kenakin, Terry et al. (2015): N-linked glycosylation of protease-activated receptor-1 at extracellular loop 2 regulates G-protein signaling bias. In *Proceedings of the National Academy of Sciences of the United States of America* 112 (27), E3600-8. DOI: 10.1073/pnas.1508838112.

Tsutsumi, Naotaka; Masoumi, Zahra; James, Sophie C.; Tucker, Julie A.; Winkelmann, Hauke; Grey, William et al. (2023): Structure of the thrombopoietin-MPL receptor complex is a blueprint for biasing hematopoiesis. In *Cell* 186 (19), 4189-4203.e22. DOI: 10.1016/j.cell.2023.07.037.

Wang, Weijia; Zhang, Yang; Dettinger, Philip; Reimann, Andreas; Kull, Tobias; Loeffler, Dirk et al. (2021): Cytokine combinations for human blood stem cell expansion induce cell-type- and cytokine-specific signaling dynamics. In *Blood* 138 (10), pp. 847–857. DOI: 10.1182/blood.2020008386.

Yamazaki, Satoshi; Iwama, Atsushi; Takayanagi, Shin-ichiro; Morita, Yohei; Eto, Koji; Ema, Hideo; Nakauchi, Hiromitsu (2006): Cytokine signals modulated via lipid rafts mimic niche signals and induce hibernation in hematopoietic stem cells. In *The EMBO journal* 25 (15), pp. 3515–3523. DOI: 10.1038/sj.emboj.7601236.

Zhang, Ping; Leger, Andrew J.; Baleja, James D.; Rana, Rajashree; Corlin, Tiffany; Nguyen, Nga et al. (2015): Allosteric Activation of a G Protein-coupled Receptor with Cell-penetrating Receptor Mimetics. In *The Journal of biological chemistry* 290 (25), pp. 15785–15798. DOI: 10.1074/jbc.M115.636316.

14th Sep 2026

Dear Prof. Modlich,

Thank you for submitting your revised files. I am pleased to inform you that your manuscript is accepted for publication and is now being sent to our publisher to be included in the next available issue of EMBO Molecular Medicine.

Your manuscript will be processed for publication by EMBO Press. It will be copy edited and you will receive page proofs prior to publication. Please note that you will be contacted by Springer Nature Author Services to complete licensing and payment information.

You may qualify for financial assistance for your publication charges - either via a Springer Nature fully open access agreement or an EMBO initiative. Check your eligibility: https://link.springer.com/journal/44321/how-to-publish-with-us

Should you be planning a Press Release on your article, please notify the production team via this form in order to coordinate publication and release dates: https://forms.cloud.microsoft/e/eH4A8JVP6g

If you have any questions, please do not hesitate to contact the Editorial Office. Thank you for your contribution to EMBO Molecular Medicine.

With kind regards,

Lise Roth

Lise Roth, Ph.D
Senior Editor
EMBO Molecular Medicine

>>> Please note that it is EMBO Molecular Medicine policy for the transcript of the editorial process (containing referee reports and your response letter) to be published as an online supplement to each paper. If you do NOT want this, you will need to inform the Editorial Office via email immediately. More information is available here: https://link.springer.com/partners/embo-press/editorial-policies#Peer%20review

EMBO Press Author Checklist

Corresponding Author Name: Ute Modlich	
Journal Submitted to: EMBO Molecular Medicine	
Manuscript Number: EMM-2026-23464	

USEFUL LINKS FOR COMPLETING THIS FORM
The EMBO Journal - Author Guidelines
EMBO Reports - Author Guidelines
Molecular Systems Biology - Author Guidelines
EMBO Molecular Medicine - Author Guidelines

Reporting Checklist for Life Science Articles (updated January
This checklist is adapted from Materials Design Analysis Reporting (MDAR) Checklist for Authors. MDAR establishes a minimum set of requirements in transparent reporting in the life sciences (see Statement of Task: 10.31222/osf.io/9sm4x). Please follow the journal's guidelines in preparing your
Please note that a copy of this checklist will be published alongside your article.

Abridged guidelines for figures
1. Data
The data shown in figures should satisfy the following conditions:
- the data were obtained and processed according to the field's best practice and are presented to reflect the results of the experiments in an accurate and unbiased manner.
- ideally, figure panels should include only measurements that are directly comparable to each other and obtained with the same assay.
- plots include clearly labeled error bars for independent experiments and sample sizes. Unless justified, error bars should not be shown for technical
- if n<5, the individual data points from each experiment should be plotted. Any statistical test employed should be justified.
- Source Data should be included to report the data underlying figures according to the guidelines set out in the authorship guidelines on Data

2. Captions
Each figure caption should contain the following information, for each panel where they are relevant:
- a specification of the experimental system investigated (eg cell line, species name).
- the assay(s) and method(s) used to carry out the reported observations and measurements.
- an explicit mention of the biological and chemical entity(ies) that are being measured.
- an explicit mention of the biological and chemical entity(ies) that are altered/varied/perturbed in a controlled manner.
- the exact sample size (n) for each experimental group/condition, given as a number, not a range;
- a description of the sample collection allowing the reader to understand whether the samples represent technical or biological replicates (including how many animals, litters, cultures, etc.).
- a statement of how many times the experiment shown was independently replicated in the laboratory.
- definitions of statistical methods and measures:
  - common tests, such as t-test (please specify whether paired vs. unpaired), simple χ2 tests, Wilcoxon and Mann-Whitney tests, can be unambiguously identified by name only, but more complex techniques should be described in the methods section;
  - are tests one-sided or two-sided?
  - are there adjustments for multiple comparisons?
  - exact statistical test results, e.g., P values = x but not P values < x;
  - definition of 'center values' as median or average;
  - definition of error bars as s.d. or s.e.m.

Please complete ALL of the questions below.
Select "Not Applicable" only when the requested information is not relevant for your study.

Materials

Newly Created Materials	Information included in the manuscript?	In which section is the information available? (Reagents and Tools Table, Materials and Methods, Figures, Data Availability Section)
New materials and reagents need to be available; do any restrictions apply?	Not Applicable	

Antibodies	Information included in the manuscript?	In which section is the information available? (Reagents and Tools Table, Materials and Methods, Figures, Data Availability Section)
For **antibodies** provide the following information:<br>- Commercial antibodies: RRID (if possible) or supplier name, catalogue number and or/clone number<br>- Non-commercial: RRID or citation	Yes	Methods, Appendix and Reagents and tools table

DNA and RNA sequences	Information included in the manuscript?	In which section is the information available? (Reagents and Tools Table, Materials and Methods, Figures, Data Availability Section)
**Short novel DNA or RNA including primers, probes**: provide the sequences.	Not Applicable	

Cell materials	Information included in the manuscript?	In which section is the information available? (Reagents and Tools Table, Materials and Methods, Figures, Data Availability Section)
**Cell lines:** Provide species information, strain. Provide accession number in repository **OR** supplier name, catalog number, clone number, and/**OR** RRID.	Not Applicable	
**Primary cultures:** Provide species, strain, sex of origin, genetic modification status.	Yes	Methods, Reagents and tools table
Report if the cell lines were recently **authenticated** (e.g., by STR profiling) and tested for mycoplasma contamination.	Not Applicable	

Experimental animals	Information included in the manuscript?	In which section is the information available? (Reagents and Tools Table, Materials and Methods, Figures, Data Availability Section)
**Laboratory animals or Model organisms:** Provide species, strain, sex, age, genetic modification status. Provide accession number in repository **OR** supplier name, catalog number, clone number, **OR** RRID.	Yes	Methods, Reagents and tools table
**Animal observed in or captured from the field:** Provide species, sex, and age where possible.	Not Applicable	
Please detail **housing and husbandry conditions**.	Yes	Methods, Reagents and tools table

Plants and microbes	Information included in the manuscript?	In which section is the information available? (Reagents and Tools Table, Materials and Methods, Figures, Data Availability Section)
**Plants:** provide species and strain, ecotype and cultivar where relevant, unique accession number if available, and source (including location for collected wild specimens).	Not Applicable	
**Microbes:** provide species and strain, unique accession number if available, and source.	Not Applicable	

Human research participants	Information included in the manuscript?	In which section is the information available? (Reagents and Tools Table, Materials and Methods, Figures, Data Availability Section)
If collected and within the bounds of privacy constraints report on age, sex and gender or ethnicity for all study participants.	Not Applicable	

Core facilities	Information included in the manuscript?	In which section is the information available? (Reagents and Tools Table, Materials and Methods, Figures, Data Availability Section)
If your work benefited from core facilities, was their service mentioned in the acknowledgments section?	Yes	Acknowledgement section

Design

Study protocol	Information included in the manuscript?	In which section is the information available? (Reagents and Tools Table, Materials and Methods, Figures, Data Availability Section)
If study protocol has been **pre-registered, provide DOI in the manuscript**. For clinical trials, provide the trial registration number **OR** cite DOI.	Not Applicable	
Report the **clinical trial registration number** (at ClinicalTrials.gov or equivalent), where applicable.	Not Applicable	

Laboratory protocol	Information included in the manuscript?	In which section is the information available? (Reagents and Tools Table, Materials and Methods, Figures, Data Availability Section)
Provide DOI OR other citation details if **external detailed step-by-step protocols** are available.	Not Applicable	

Experimental study design and statistics	Information included in the manuscript?	In which section is the information available? (Reagents and Tools Table, Materials and Methods, Figures, Data Availability Section)
Include a statement about **sample size** estimate even if no statistical methods were used.	Yes	Methods
Were any steps taken to minimize the effects of subjective bias when allocating animals/samples to treatment (e.g. **randomization procedure**)? If yes, have they been described?	Yes	Methods
Include a statement about **blinding** even if no blinding was done.	Yes	Methods
Describe **inclusion/exclusion criteria** if samples or animals were excluded from the analysis. Were the criteria pre-established?<br><br>If sample or data points were omitted from analysis, report if this was due to attrition or intentional exclusion and provide justification.	Yes	Methods
For every figure, are **statistical tests** justified as appropriate? Do the data meet the assumptions of the tests (e.g., normal distribution)? Describe any methods used to assess it. Is there an estimate of variation within each group of data? Is the variance similar between the groups that are being statistically compared?	Yes	Methods

Sample definition and in-laboratory replication	Information included in the manuscript?	In which section is the information available? (Reagents and Tools Table, Materials and Methods, Figures, Data Availability Section)
In the figure legends: state number of times the experiment was **replicated** in laboratory.	Yes	Figure Legends
In the figure legends: define whether data describe **technical or biological replicates**.	Yes	Figure Legends

Ethics

Ethics	Information included in the manuscript?	In which section is the information available? (Reagents and Tools Table, Materials and Methods, Figures, Data Availability Section)
Studies involving **human participants**: State details of **authority granting ethics approval** (IRB or equivalent committee(s), provide reference number for approval.	Not Applicable	
Studies involving **human participants**: Include a statement confirming that **informed consent** was obtained from all subjects and that the experiments conformed to the principles set out in the WMA Declaration of Helsinki and the Department of Health and Human Services Belmont Report.	Not Applicable	
Studies involving **human participants: For publication of patient photos**, include a statement confirming that consent to publish was obtained.	Not Applicable	
Studies involving experimental **animals**: State details of **authority granting ethics approval** (IRB or equivalent committee(s), provide reference number for approval. Include a statement of compliance with ethical regulations.	Yes	Methods
Studies involving **specimen and field samples:** State if relevant **permits** obtained, provide details of authority approving study; if none were required, explain why.	Not Applicable	

Dual Use Research of Concern (DURC)	Information included in the manuscript?	In which section is the information available? (Reagents and Tools Table, Materials and Methods, Figures, Data Availability Section)
Could your study fall under dual use research restrictions? Please check biosecurity documents and list of **select agents and toxins** (CDC): https://www.selectagents.gov/sat/list.htm	Not Applicable	
If you used a select agent, is the security level of the lab appropriate and reported in the manuscript?	Not Applicable	
If a study is subject to dual use research of concern regulations, is the name of the **authority granting approval and reference number** for the regulatory approval provided in the manuscript?	Not Applicable	

Reporting

The MDAR framework recommends adoption of discipline-specific guidelines, established and endorsed through community initiatives. Journals have their own policy about requiring specific guidelines and recommendations to complement MDAR.

Adherence to community standards	Information included in the manuscript?	In which section is the information available? (Reagents and Tools Table, Materials and Methods, Figures, Data Availability Section)
State if relevant guidelines or checklists (e.g., **ICMJE, MIBBI, ARRIVE, PRISMA**) have been followed or provided.	Yes	Methods
For **tumor marker prognostic studies,** we recommend that you follow the **REMARK** reporting guidelines (see link list at top right). See author guidelines, under 'Reporting Guidelines'. Please confirm you have followed these guidelines.	Not Applicable	
For **phase II and III randomized controlled trials**, please refer to the **CONSORT** flow diagram (see link list at top right) and submit the CONSORT checklist (see link list at top right) with your submission. See author guidelines, under 'Reporting Guidelines'. Please confirm you have submitted this list.	Not Applicable	

Data Availability

Data availability	Information included in the manuscript?	In which section is the information available? (Reagents and Tools Table, Materials and Methods, Figures, Data Availability Section)
Have **primary datasets** been deposited according to the journal's guidelines (see 'Data Deposition' section) and the respective accession numbers provided in the Data Availability Section?	Yes	Data Availability Section
Were **human clinical and genomic datasets** deposited in a public access-controlled repository in accordance to ethical obligations to the patients and to the applicable consent agreement?	Not Applicable	
Are **computational models** that are central and integral to a study available without restrictions in a machine-readable form? Were the relevant accession numbers or links provided?	Not Applicable	
If publicly available data were reused, provide the respective **data citations in the reference list**.	Not Applicable	
