# Supplementary material for: Activated protein C promotes hematopoietic stem and progenitor cell quiescence and engraftment
Source: EMBO Mol Med. 2026 Oct 9;18(11):4433–67. doi: 10.1038/s44321-026-00529-y (PMC13748566; doi:10.1038/s44321-026-00529-y)
Supplement: Supplementary file 3 [file 44321_2026_529_MOESM9_ESM.pdf]

Expanded View Figures

Figure EV1. Expression of PAR1 and EPCR of human bone marrow CD34+ subpopulations.

(**A**) Representative FC plots showing EPCR and PAR1 expression on indicated populations of BM CD34+ samples at day 0 and 2 days in culture. (**B**, **C**) PAR1 and EPCR expression during in vitro cultivation are shown in BM samples on the indicated populations, $n = 3$ BM donors. (**D**) MFI of PAR1-PE on indicated populations of BM samples 2 days in culture, $n = 3$ BM donors. (**E**) Cell surface and intracellular (fix perm.) PAR1 expression quantified in 2-day cultivated CD34+ cells with and without THR stimulation for 1 h, PAR1 FMOs are depicted as the dotted thin-lined histograms, $n = 4$ mPB donors. (**F**) Staining of EPCR on CD34+ cells after 2 days in culture, cells were either incubated for 1 h with aPC (10 µg/ml) at 37 °C or left unstimulated in normal medium before cell surface marker staining was conducted, three different commercially available monoclonal anti-EPCR antibodies were used (clones RCR-401 and RCR-252, also known as REA337), $n = 4$ mPB donors. (**G**) Cell surface PAR1 staining of 2-day cultivated CD34+ cells with aPC alone (blue) or in the presence of EPCR-blocking antibody REA337 (red), $n = 4$ mPB donors. Statistical analysis: (**B–G**) all data were presented as mean ± SD. (**B**, **C**, **E**) Two-way ANOVA with Holm–Šídák's multiple comparison test. (**C**) See Appendix Table S1 for two-way ANOVA comparing CD90+ with CD90− cells for each day. (**D**, **G**) One-way ANOVA with Tukey's multiple comparison test. (**F**) Unpaired two-tailed *t*-tests; ns not significant, *$P \leq 0.05$, **$P \leq 0.01$, ***$P \leq 0.001$, ****$P \leq 0.0001$, exact *P* values can be found in Appendix Table S9.

 © The Author(s)

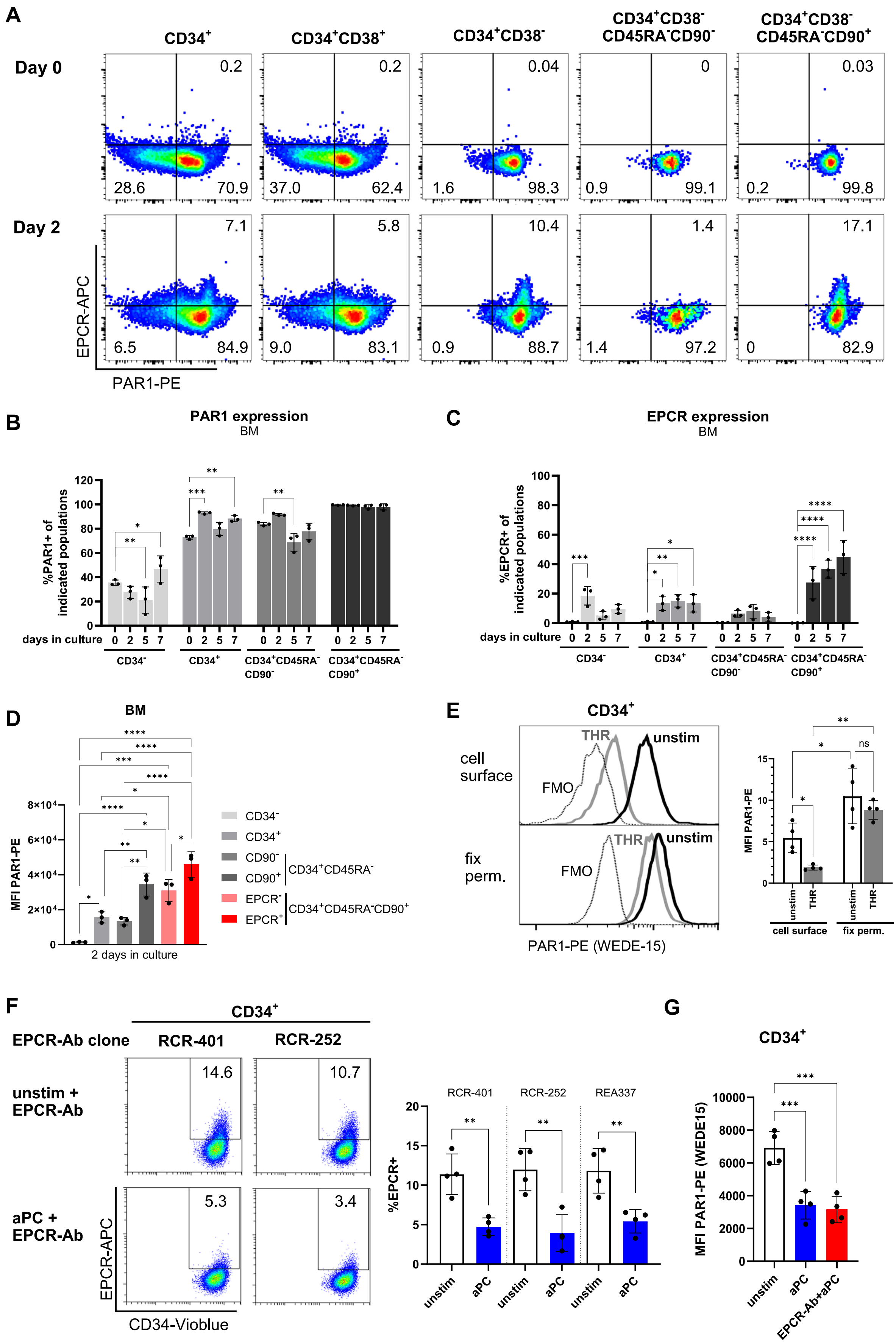
A
CD34+
CD34+CD38+
CD34+CD38-
CD34+CD38- CD45RA-CD90-
CD34+CD38- CD45RA-CD90+
Day 0
Day 2
EPCR-APC
PAR1-PE
B
PAR1 expression
BM
%PAR1+ of indicated populations
days in culture
C
EPCR expression
BM
%EPCR+ of indicated populations
D
BM
MFI PAR1-PE
2 days in culture
CD34-
CD34+
CD90-
CD90+
CD34+CD45RA-
EPCR-
EPCR+
CD34+CD45RA-CD90+
E
CD34+
cell surface
fix perm.
THR
unstim
FMO
PAR1-PE (WEDE-15)
MFI PAR1-PE
F
CD34+
EPCR-Ab clone
RCR-401
RCR-252
unstim + EPCR-Ab
aPC + EPCR-Ab
EPCR-APC
CD34-Vioblue
REA337
%EPCR+
unstim
aPC
G
CD34+
MFI PAR1-PE (WEDE15)
EPCR-Ab+aPC

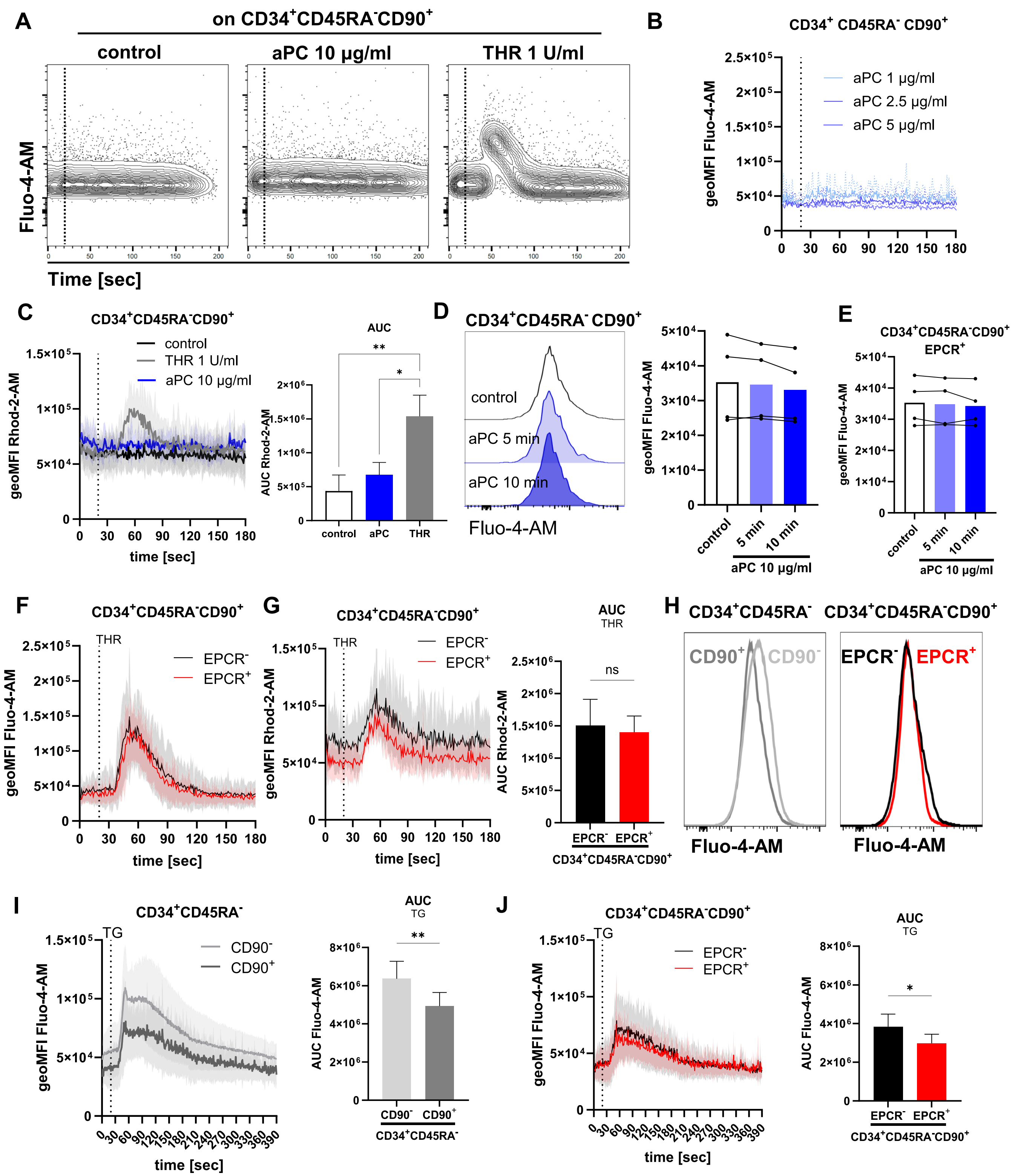
A
on CD34+CD45RA-CD90+
control
aPC 10 µg/ml
THR 1 U/ml
Fluo-4-AM
Time [sec]
B
CD34+ CD45RA- CD90+
aPC 1 µg/ml
aPC 2.5 µg/ml
aPC 5 µg/ml
geoMFI Fluo-4-AM
C
CD34+CD45RA-CD90+
control
THR 1 U/ml
aPC 10 µg/ml
geoMFI Rhod-2-AM
time [sec]
AUC
AUC Rhod-2-AM
control
aPC
THR
D
CD34+CD45RA- CD90+
control
aPC 5 min
aPC 10 min
Fluo-4-AM
geoMFI Fluo-4-AM
control
5 min
10 min
aPC 10 µg/ml
E
CD34+CD45RA-CD90+
EPCR+
geoMFI Fluo-4-AM
control
5 min
10 min
aPC 10 µg/ml
F
CD34+CD45RA-CD90+
THR
EPCR-
EPCR+
geoMFI Fluo-4-AM
time [sec]
G
CD34+CD45RA-CD90+
THR
EPCR-
EPCR+
geoMFI Rhod-2-AM
time [sec]
AUC
THR
ns
AUC Rhod-2-AM
EPCR- EPCR+
CD34+CD45RA-CD90+
H
CD34+CD45RA-
CD34+CD45RA-CD90+
CD90+ CD90-
EPCR- EPCR+
Fluo-4-AM
Fluo-4-AM
I
CD34+CD45RA-
TG
CD90-
CD90+
geoMFI Fluo-4-AM
time [sec]
AUC
TG
AUC Fluo-4-AM
CD90- CD90+
CD34+CD45RA-
J
CD34+CD45RA-CD90+
TG
EPCR-
EPCR+
geoMFI Fluo-4-AM
time [sec]
AUC
TG
AUC Fluo-4-AM
EPCR- EPCR+
CD34+CD45RA-CD90+

 © The Author(s)

Figure EV2. PAR1-Ca2 + -signaling in human CD34+ cells.

(**A**) Representative flow cytometry plots of the Ca2+ flux assay, shown is the fluorescent signal of Fluo-4-AM over time of the sample recording gated on CD34+CD45RA−CD90+ cells. The dotted line marks the 20 s, where indicated stimuli were added to the sample. (**B**) Fluo-4-AM kinetics of cells stimulated with various aPC doses (1, 2.5, 5 µg/ml) after 20 s, marked by the dotted line, $n = 3$ mPB donors. (**C**) Ca2+ kinetics in CD34+CD45RA−CD90+ cells in response to aPC, THR or control, measured with Rhod-2, $n = 3$ mPB donors. (**D**) aPC does not lead to changes in Ca2+ concentration after 5 and 10 min in CD34+CD45RA−CD90+ and (**E**) CD34+CD45RA−CD90+EPCR+ cells, $n = 4$ mPB donors. (**F**) Fluo-4-AM kinetics and (**G**) Rhod-2 kinetics and AUC of CD34+CD45RA−CD90+EPCR+/− cells after THR stimulation (1 U/ml), (**F**) $n = 6$ mPB donors, (**G**) $n = 3$ mPB donors. (**H**) Representative plots of Fluo-4-AM signal at steady state in CD34+CD45RA−CD90+/− and CD34+CD45RA−CD90+EPCR+/−. (**I**) Ca2+ flux caused by Thapsigargin (TG, 1 µM), shown is kinetic curves and areas under the curves (AUC) of CD34+CD45RA−CD90+/−, $n = 6$ mPB and (**J**) CD34+CD45RA−CD90+EPCR+/−, $n = 5$ mPB. Statistical analysis: (**B**, **C**, **F–J**) data presented as mean ± SD. (**D**, **E**) The bar represents the mean, and the dots connected by a line represent the data from each individual donor. (**C–E**) one-way ANOVA with Tukey's multiple comparison test. (**G**, **I**, **J**) Bar chart: unpaired two-tailed *t*-test; ns not significant, *$P \leq 0.05$, **$P \leq 0.01$, ***$P \leq 0.001$, ****$P \leq 0.0001$, exact *P* values can be found in Appendix Table S10.

© The Author(s)

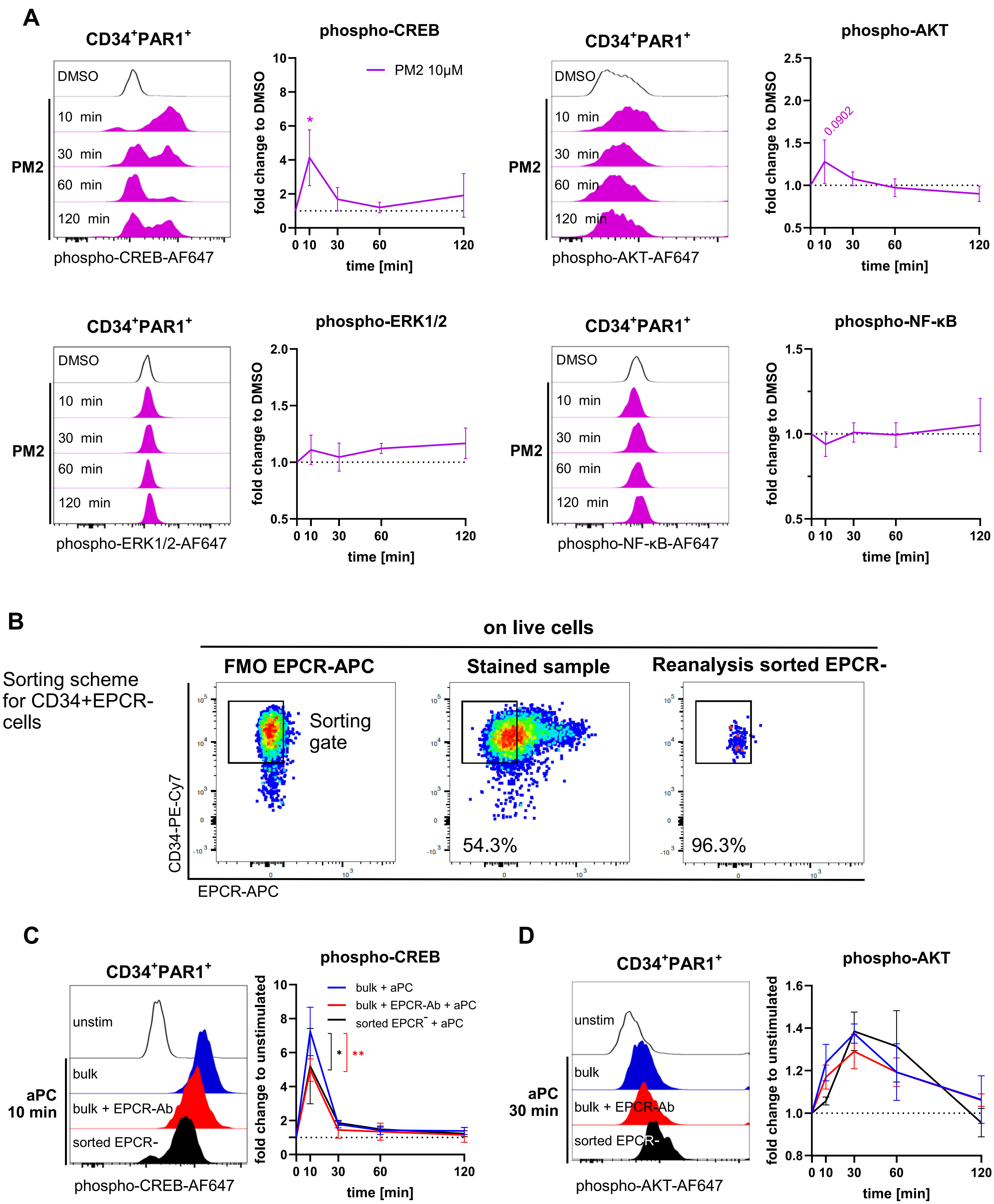
A
CD34+PAR1+
DMSO
10 min
30 min
60 min
120 min
PM2
phospho-CREB-AF647
phospho-CREB
PM2 10µM
*
fold change to DMSO
time [min]
phospho-AKT-AF647
phospho-AKT
0.0902
phospho-ERK1/2-AF647
phospho-ERK1/2
phospho-NF-κB-AF647
phospho-NF-κB
B
Sorting scheme for CD34+EPCR- cells
on live cells
FMO EPCR-APC
Stained sample
Reanalysis sorted EPCR-
Sorting gate
54.3%
96.3%
CD34-PE-Cy7
EPCR-APC
C
CD34+PAR1+
unstim
bulk
bulk + EPCR-Ab
sorted EPCR-
aPC 10 min
phospho-CREB-AF647
phospho-CREB
bulk + aPC
bulk + EPCR-Ab + aPC
sorted EPCR- + aPC
fold change to unstimulated
D
aPC 30 min
phospho-AKT-AF647
phospho-AKT

 © The Author(s)

Figure EV3. EPCR-independent PAR1 signaling in CD34 + PAR1+ cells.

(**A**) phosphoflow analysis of phosphorylated CREB, AKT, ERK1/2 and NfκB of 7-day expanded CD34+ cells, pregated on CD34+PAR1+ cells in response to PM2 (10 μM) for the indicated time period, depicted as fold change to the DMSO control, $n = 3$ mPB donors. (**B**) Representative FACS sorting scheme to sort CD34+EPCR− cells at day 7 for phosphoflow analysis. (**C**) Phosphoflow analysis of CREB and (**D**) AKT in response to aPC, either on bulk expanded cells without (blue) or with EPCR-blocking antibody (clone REA337, red) or in sorted EPCR-negative CD34+ cells (back), $n = 3$ mPB donors. Statistical analysis: (**A**, **C**, **D**) Data presented as mean ± SD. (**A**) One-way ANOVA with Dunnett's multiple comparison test compared to the DMSO control. (**C**, **D**) Two-way Anova with Holm–Šídák's multiple comparison test; ns not significant, *$P \leq 0.05$, **$P \leq 0.01$, ***$P \leq 0.001$, ****$P \leq 0.0001$, exact *P* values can be found in Appendix Table S11.

© The Author(s)

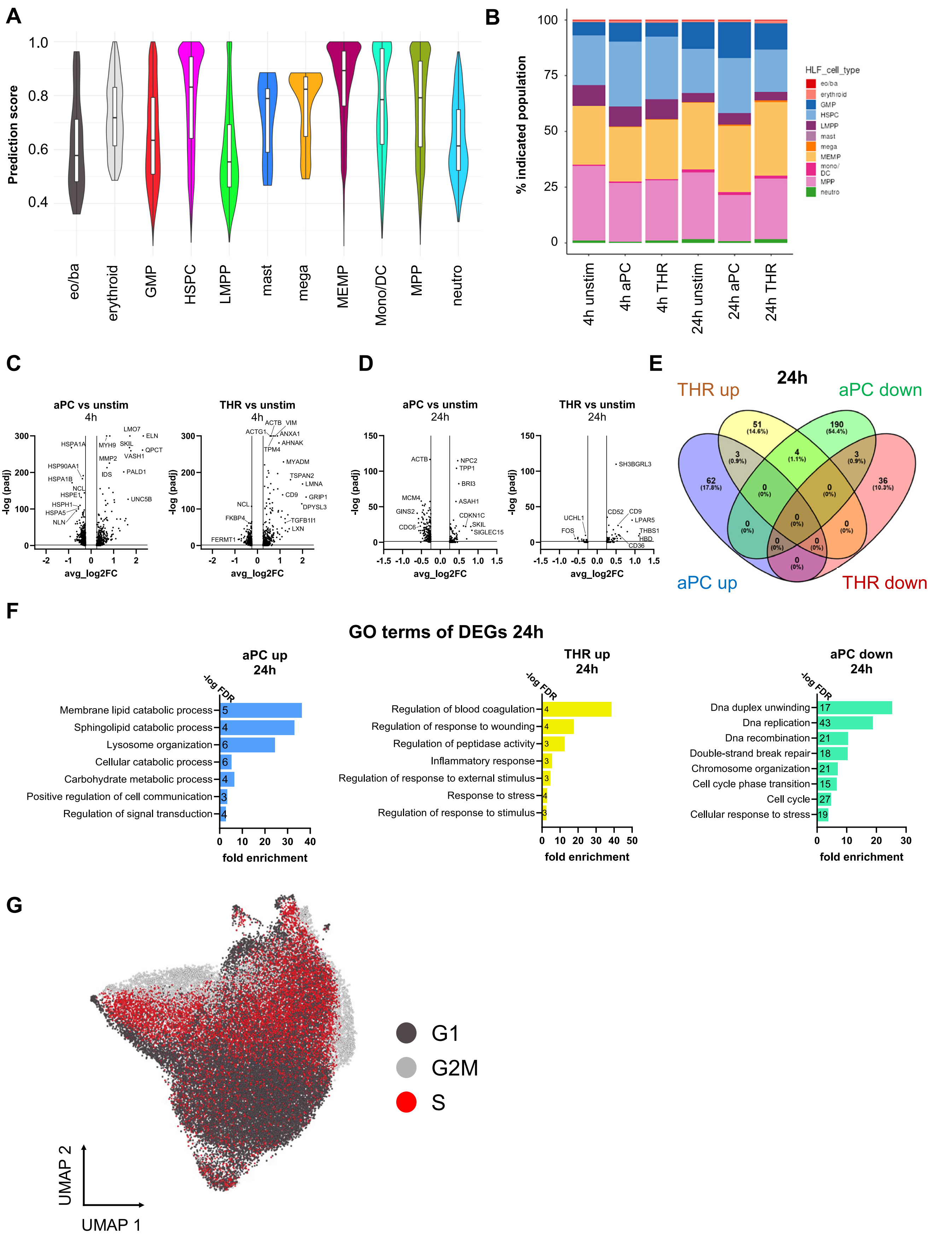
A
Prediction score
eo/ba
erythroid
GMP
HSPC
LMPP
mast
mega
MEMP
Mono/DC
MPP
neutro
B
% indicated population
HLF_cell_type
4h unstim
4h aPC
4h THR
24h unstim
24h aPC
24h THR
C
aPC vs unstim 4h
THR vs unstim 4h
D
aPC vs unstim 24h
THR vs unstim 24h
-log (padj)
avg_log2FC
E
24h
THR up
aPC down
aPC up
THR down
F
GO terms of DEGs 24h
aPC up 24h
Membrane lipid catabolic process
Sphingolipid catabolic process
Lysosome organization
Cellular catabolic process
Carbohydrate metabolic process
Positive regulation of cell communication
Regulation of signal transduction
THR up 24h
Regulation of blood coagulation
Regulation of response to wounding
Regulation of peptidase activity
Inflammatory response
Regulation of response to external stimulus
Response to stress
Regulation of response to stimulus
aPC down 24h
Dna duplex unwinding
Dna replication
Dna recombination
Double-strand break repair
Chromosome organization
Cell cycle phase transition
Cell cycle
Cellular response to stress
-log FDR
fold enrichment
G
G1
G2M
S
UMAP 1
UMAP 2

 © The Author(s)

Figure EV4. scRNAseq of stimulated CD34+ cells.

(**A**) Prediction scores for each cluster are depicted $n = 14{,}171$ (MEMP), $n = 13{,}713$ (MPP), $n = 11{,}989$ (HSPC), $n = 5018$ (GMP), $n = 3422$ (LMPP), $n = 578$ (neutro), $n = 499$ (mono/DC), $n = 490$ (erythroid), $n = 167$ (mega), $n = 107$ (eo/ba), $n = 14$ (mast). (**B**) Proportions of clusters for each sample. (**C**) Volcano plot depicting DEG after 4 h and (**D**) 24 h with aPC or THR stimulation compared to unstimulated, $n = 8602$ cells 4 h unstim, $n = 9024$ cells 4 h aPC, $n = 8170$ cells 4 h THR, $n = 6965$ cells 24 h unstim, $n = 8507$ cells 25 aPC, $n = 8900$ cells 24 h THR from one donor in one experiment. (**E**) Venn diagram showing shared and unique DEG after 24 h. (**F**) GO biological processes terms enriched in up- and downregulated genes after 24 h with aPC and THR, of note: THR downregulated genes at 24 h were not enriched for any GO term, number in bar represents −log (FDR) rounded to nearest integer, all terms are below threshold of FDR <0.05. (**G**) UMAP depicting the cell cycle phase of CD34+ cells. Statistical analysis: (**A**) thick horizontal line indicates median, box indicates interquartile range and whiskers indicate range. (**C**, **D**) Wilcoxon rank-sum test with Bonferroni correction.

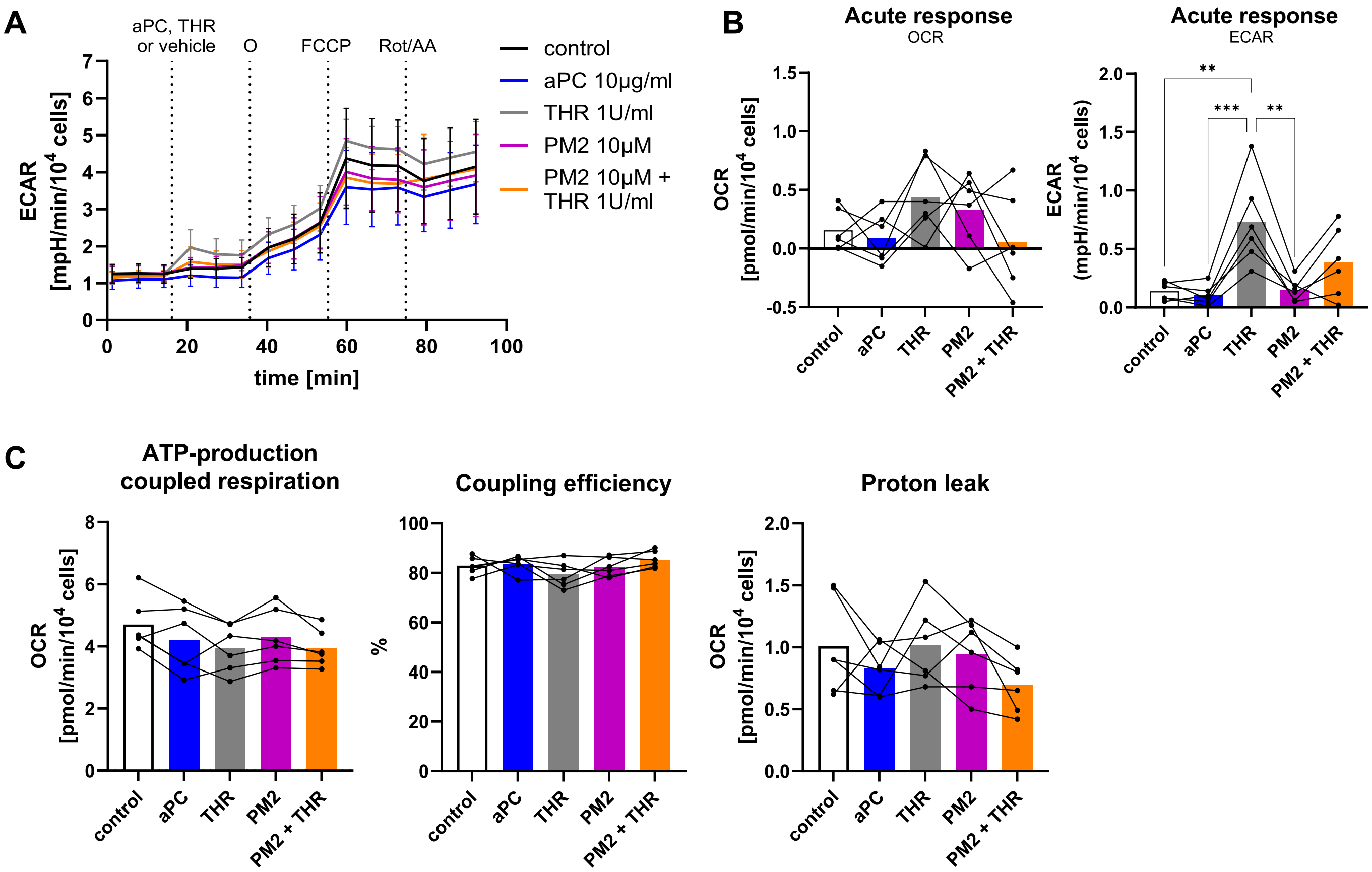

Figure EV5. metabolic parameters of CD34+ cells.

(**A**) Extracellular acidification rate (ECAR) curves over the course of the Seahorse Mitostress test, dotted lines represent injection of indicated stimuli, $n = 6$ mPB donors. (**B**) Acute response of CD34+ cells to indicated stimuli, data shown here is also shown in Fig. 4B, $n = 6$ mPB donors. (**C**) Metabolic parameters derived from the Mitostress test of CD34+ cells, $n = 6$ mPB donors. Statistical analysis: (**A**) data were presented as mean ± SD. (**B**, **C**) Bar height represents the mean, lines connect samples from individual donors, one-way Anova with Tukey's multiple comparison test; $^*P \leq 0.05$, $^{**}P \leq 0.01$, $^{***}P \leq 0.001$, $^{****}P \leq 0.0001$, exact P values can be found in Appendix Table S12.

 © The Author(s)

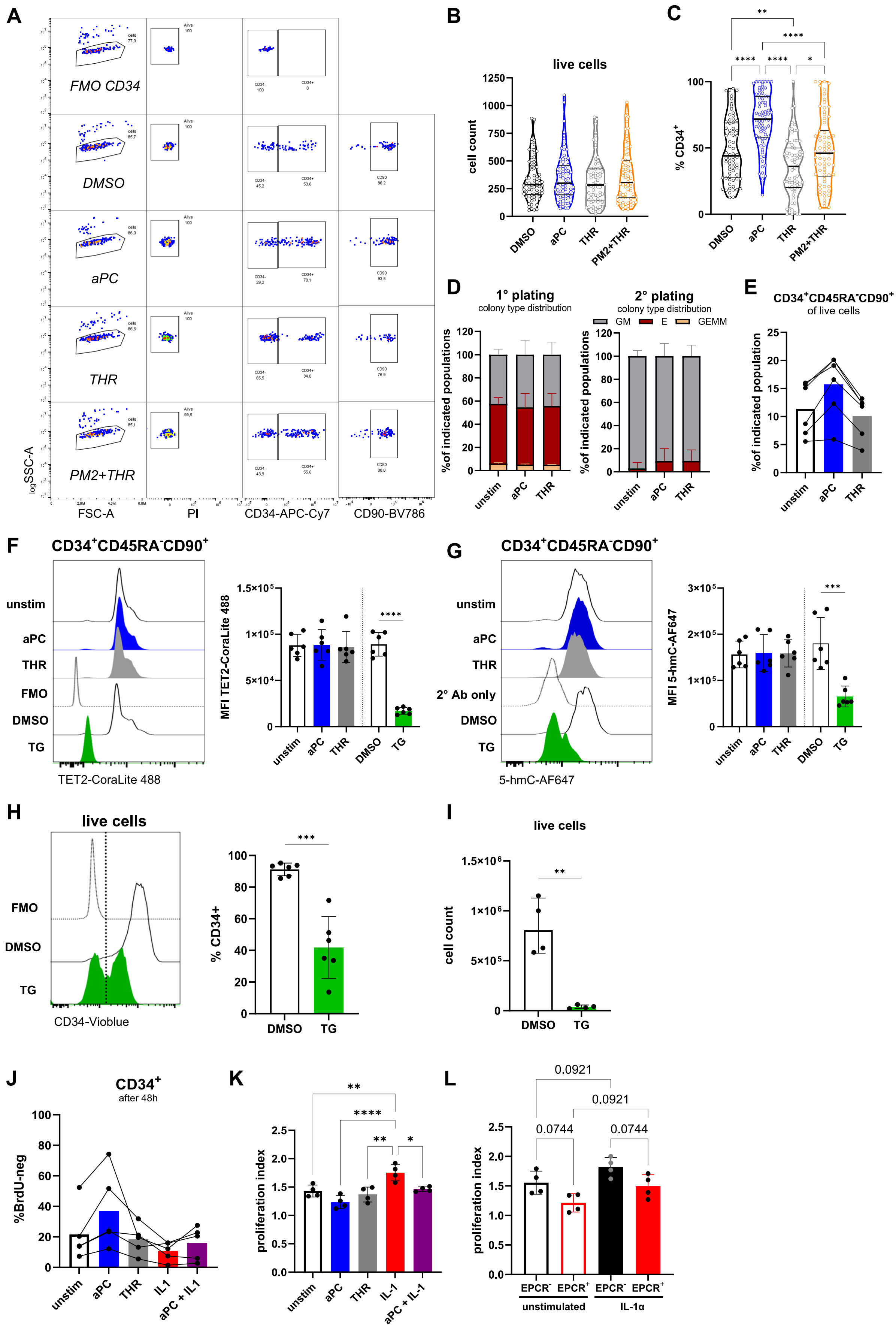
A
FMO CD34
DMSO
aPC
THR
PM2+THR
logSSC-A
FSC-A
PI
CD34-APC-Cy7
CD90-BV786
B
live cells
cell count
DMSO aPC THR PM2+THR
C
% CD34+
D
1° plating
colony type distribution
2° plating
colony type distribution
GM E GEMM
%of indicated populations
unstim aPC THR
E
CD34+CD45RA-CD90+
of live cells
%of indicated population
F
CD34+CD45RA-CD90+
unstim
aPC
THR
FMO
DMSO
TG
TET2-CoraLite 488
MFI TET2-CoraLite 488
G
CD34+CD45RA-CD90+
2° Ab only
5-hmC-AF647
MFI 5-hmC-AF647
H
live cells
CD34-Vioblue
% CD34+
I
live cells
cell count
J
CD34+
after 48h
%BrdU-neg
unstim aPC THR IL1 aPC + IL1
K
proliferation index
unstim aPC THR IL-1 aPC + IL-1
L
0.0921
0.0921
0.0744
0.0744
proliferation index
EPCR- EPCR+ EPCR- EPCR+
unstimulated
IL-1α

Figure EV6. In vitro culture and colony formation after stimulation with aPC or THR.

(**A**) Representative FC gating for analysis of colonies from the single-cell expansion assay. (**B**) Colony size (live cells per colony) and (**C**) frequency of CD34+ cells in colonies after 7 days from single-cell expansion assay, $n = 69$–71 colonies from three independent experiments, 1 mPB donor. (**D**) Colony type distribution of CD34+ cells in primary and secondary plating of colony-forming unit (CFU) assay, CD34+ cells were cultivated for 2 days prior to stimulation with aPC (10 µg/ml) or THR (1 U/ml) for 1 h before primary plating, $n = 3$ mPB donors. (**E**) Frequency of HSC-enriched CD34+CD45RA−CD90+ cells within all live cells after 7 days, $n = 6$ mPB donors. (**F**) TET2 expression and (**G**) 5-hydroxymethylcytosine (5-hmC) levels in CD34+CD45RA−CD90+ cells after 7 days of cultivation with normal HSC medium (unstim), aPC (10 µg/ml), THR (1 U/ml), DMSO (0.1%), or Thapsigargin (TG, 1 µM), $n = 6$ mPB donors. (**H**) Frequency of CD34+ cells after 7 days of cultivation with DMSO (0.1%) or TG (1 µM), $n = 6$ mPB donors. (**I**) number of live cells per well after 7 days of cultivation, starting from 7.5×10^4 cells, $n = 4$ mPB donors. (**J**) Bar chart showing incorporation of BrdU into the genome of CD34+ cells after 2 days in culture with the following stimuli: aPC (10 µg/ml), THR (1 U/ml), Il-1α (10 ng/ml), or aPC (10 µg/ml) and IL-1α (10 ng/ml) or unstimulated, $n = 4$ mPB donors. (**K**) proliferation indices of CD34 + CD45RA-CD90+ after 3 days in culture with the following stimuli: aPC (10 µg/ml), THR (1 U/ml), Il-1α (10 ng/ml) or aPC (10 µg/ml), and IL-1α (10 ng/ml) or unstimulated, $n = 4$ mPB donors. (**L**) proliferation indices of CD34+CD45RA−CD90+EPCR +/− after 3 days in culture with or without Il-1α (10 ng/ml), $n = 4$ mPB donors. Statistical analysis: (**B**, **C**) thick line indicates median, thin line indicates interquartile. (**D**, **F**–**I**, **K**, **L**) Data were mean ± SD. (**E**, **J**) Bar height represents the mean, lines connect samples from individual donors. (**B**–**E**, **J**, **K**) One-way ANOVA with Tukey's multiple comparison test. (**F**, **G**) Unstim vs. aPC vs. THR: one-way Anova with Tukey's multiple comparison test, DMSO vs. TG: unpaired two-tailed *t*-test, (**H**, **I**) unpaired two-tailed *t*-test, (**L**) two-way Anova with Holm–Šídák's multiple comparison test; $^*P \le 0.05$, $^{**}P \le 0.01$, $^{***}P \le 0.001$, $^{****}P \le 0.0001$, exact *P* values can be found in Appendix Table S13.

 © The Author(s)

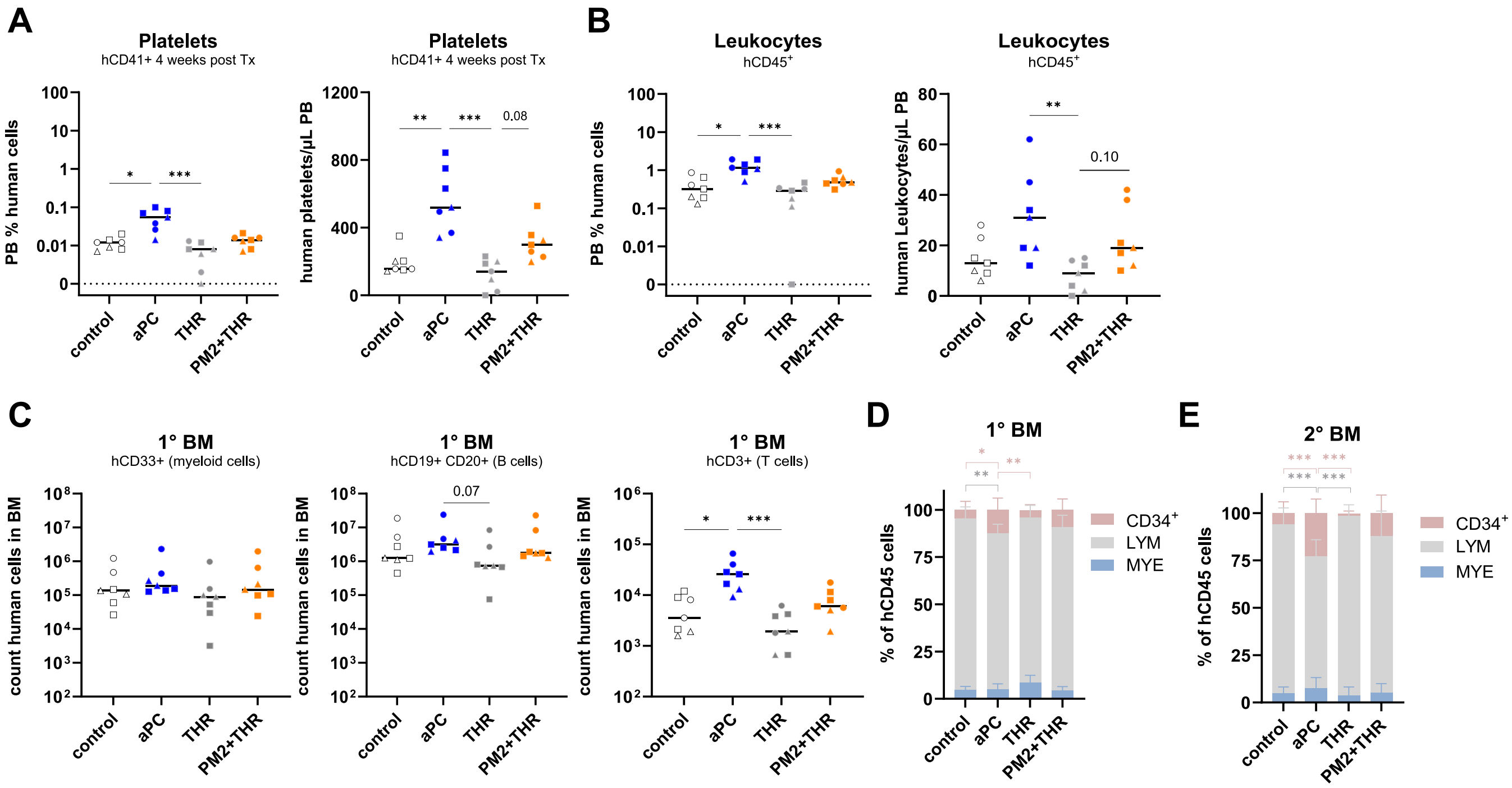

Figure EV7. Peripheral blood and multilineage engraftment of human cells in NBSGW mice.

(**A**) Frequency and cell counts/µl of human platelets (hCD41+) are shown 4 weeks after transplantation of stimulated CD34+ cells into NBSGW mice. aPC ($n = 7$), THR ($n = 7$), PM2 + THR ($n = 7$), and control (DMSO, $n = 7$). Different symbol shapes indicate three independent performed stimulations and transplantations. (**B**) Frequency and cell counts/µl of human leukocytes (hCD45+) are shown 4 weeks after transplantation of stimulated CD34+ cells into NBSGW mice. (**C**) Counts of human CD33+ (myeloid), CD19/CD20+ (B cells), hCD3+ (T cells) in the BM of transplanted NBSGW mice after 4 weeks. (**D**) relative contribution of CD34 +, myeloid (MYE) and lymphoid (LYM, B + T cells) in BM of 1° and (**E**) 2° transplanted NBSGW mice after 4 weeks. Statistical analysis: (**A**–**C**) Data represented as median, non-parametric one-way-ANOVA with Kruskal–Wallis test. (**D**, **E**) Data represented as mean ± SD, two-way Anova with Holm–Šídák's multiple comparison test, only selected comparisons are shown; $*P \leq 0.05$, $**P \leq 0.01$, $***P \leq 0.001$, $****P \leq 0.0001$, exact P values can be found in Appendix Table S14.
